# Supplementary material for: Opioid-free anesthesia compared to opioid anesthesia for lung cancer patients undergoing video-assisted thoracoscopic surgery: A randomized controlled study
Source: PLoS One. 2021 Sep 23;16(9):e0257279. doi: 10.1371/journal.pone.0257279 (PMC8460000; doi:10.1371/journal.pone.0257279)
Supplement: S1 File — (DOCX) [file pone.0257279.s002.docx]

**申明**

我确认我作为补充信息所包含的临床试验方案是在试验开始前提交给伦理委员会并经其批准的版本。

**无阿片类药物的麻醉方法用于胸腔镜肺手术的临床研究**

方案版本：**2018-6-20 版本号2.0**

试验参与者：

| ` | 姓名： | 研究分工 |
| --- | --- | --- |
| 高级职称 | 赵雪莲 | 试验方案设计 |
|  | 付建峰 | 试验的研究背景 |
| 初级职称 | 张译文 | 患者入组，术中麻醉维护和样本采集 |
|  | 张沙沙 | 患者的预后观察和随访 |
| 研究生 | 陈诺娅 | 患者术后观察，数据采集及分析 |

申办者：赵雪莲主任医师，赵雪莲对该研究在试验设计、数据收集、方案管理、数据分析判读、撰写报告和文章发表过程中所承担的任务具有最终决定权。

**一、研究背景：**

完整的全身麻醉由镇静、镇痛和肌松三部分组成。其中，充分的镇痛是贯穿于术中以及术后直接影响到患者生活质量和病情恢复的重要一环。传统的全身麻醉中，阿片类镇痛药物以其强大且确切的镇痛效果占据着全麻镇痛中最重要的地位。但近年来，随着人们越来越多的关注到术后恢复情况和远期预后，以及对术后快速康复理念的认识加深，越来越多的研究关注到了全麻术中及术后所应用的阿片类镇痛药物的副作用上，如呼吸系统并发症、恶心呕吐、对胃肠道蠕动的抑制作用、社会潜在成瘾问题、免疫抑制作用及其对肿瘤转移的影响，延长了患者（尤其是消化道手术患者）术后排气时间和恢复时间，增加了围术期呼吸系统风险，增加了住院时间和住院费用等。除此以外，外科手术微创技术的发展，对镇痛强度的需求也有所下降，这就使低阿片麻醉或无阿片麻醉成为可能。

本研究想要找到一种可以替代术中以及术后阿片类镇痛药物的全麻方法，在保证给予患者充分镇痛的基础上，用其它药物或手段替代阿片类镇痛药。我们知道，镇痛方法主要有药物镇痛和神经阻滞镇痛两大类，而前者包括阿片类镇痛药、a2-受体激动剂、局麻药和非甾体镇痛药（NSAIDs）。右美托咪定（dexmedetomidine，Dex）是一种强效的高选择性a2肾上腺素受体激动剂，作用于脑和脊髓的a2受体。Dex具有镇静、镇痛、顺性遗忘和抗交感神经等作用，其可提高患者围术麻醉期的舒适度，有效缓解患者围术期的应激反应，保持血流动力学稳定，减少术后疼痛反应，在临床麻醉和镇痛中已成为一种安全、有效的辅助用药。NSAIDs是治疗轻、中度疼痛的一线用药。目前提倡的多模式镇痛即强调联合不同作用机制的镇痛药物或不同镇痛措施，通过多种机制产生镇痛作用，以获得更好的镇痛效果，从而使药物副作用减到最少。研究表明，联合NSAIDs术后镇痛获益明确，可减少阿片类的不良反应如恶心、呕吐、嗜睡等。区域神经阻滞被认为是多模式镇痛的“基石”。当前，区域神经阻滞技术随着超声技术在麻醉镇痛领域的推广得到了迅猛发展，区域神经阻滞在超声可视化技术的支持下，变得简便、安全、效果确切。本研究拟选取右美托咪定、NSAIDs、七氟烷 复合胸椎旁阻滞的方法，探讨无阿片类镇痛药的全麻在胸腔镜肺手术中实施的可行性。

1. **研究目标：**

探究无阿片类镇痛药物的全麻和传统全麻对胸腔镜肺手术患者术中、术后及预后的影响及可行性。

**三、试验设计**

1. 研究现场：河北医科大学第四医院手术室，术后随访在河北医科大学第四医院胸外科病房。
2. 样本量估算：使用PASS11.0进行样本量估算，每组需50例，考虑到失访者、操作失败者以及中途退出试验者，故每组55人，样本总量为110例。
3. 募集受试者：河北医科大学第四医院胸外科住院病人。
4. 保密制度：本项目研究的结果可能会在医学杂志上发表，受试者的个人信息将受到严格保密，除非应相关法律要求，患者个人信息不会被泄露。必要时，政府管理部门和医院伦理委员会及其它相关研究人员可以按规定查阅您的资料。
5. 盲法及隐蔽分组：通过随机数表法将患者分为2组，研究人员将按照分组结果对患者实施相应的麻醉处理，对随访者实施单盲。
6. 受试者入组及分组
   - 1. 纳入标准：拟行胸腔镜肺手术的患者，性别不限，年龄18~65岁，美国麻醉医师协会分级Ⅰ/Ⅱ级，并签署知情同意书。
     2. 排除标准：有区域神经阻滞禁忌者；脑血管疾病、消化系统疾病（如消化性溃疡）、肝肾功能异常、糖尿病、血液系统功能障碍患者；慢性疼痛、长期使用阿片药物、激素或非甾体类药物者；局麻药过敏者；孕期及哺乳期妇女；精神疾病患者、无法配合术后疼痛评分者；心肺功能中度及以上异常者；患有青光眼、前列腺肥大等禁用阿托品的疾病的患者；术中术式改为开放手术的患者。

③分组：将按随机数表法分为2组：试验组为无阿片全麻组（*n_1_*=55），对照组为传统全麻组（*n_2_*=55）。

干预措施：患者入室后行常规监测，试验组给予右美托咪定，然后行超声引导下胸椎旁阻滞，10~20分钟后测量并记录阻滞平面，静脉滴注酮咯酸氨丁三醇，给予顺式阿曲库铵、依托咪酯完成麻醉诱导，维持阶段采用七氟醚吸入和右美托咪定持续泵注；对照组直接行超声引导下胸椎旁阻滞，10~20分钟后测量并记录阻滞平面，随后用舒芬太尼、顺式阿曲库铵、依托咪酯完成麻醉诱导，维持阶段采用七氟醚吸入和瑞芬太尼持续泵注。

7.麻醉方法：患者进入手术室后,行心电、脉搏氧饱和度监测，局麻下行左桡动脉穿刺置管,右颈内中心静脉置管，持续监测有创动脉压、中心静脉压和镇静指数等指标。

①麻醉诱导：麻醉前用药为咪达唑仑、阿托品和（或）盐酸戊乙奎醚。诱导用药：试验组先给予右美托咪定，然后行超声引导下单侧胸椎旁阻滞，10~20分钟后测量并记录阻滞平面，静脉滴注酮咯酸氨丁三醇，之后给予依托咪酯、顺式阿曲库铵行麻醉诱导；对照组直接行超声引导下单侧胸椎旁阻滞，10~20分钟后测量并记录阻滞平面，然后给予舒芬太尼、依托咪酯和顺式阿曲库铵行麻醉诱导。两组均使用光棒或可视喉镜行气管插管，行呼末二氧化碳监测和麻醉气体监测。

②麻醉维持：试验组采用七氟醚吸入和右美托咪定持续泵注，对照组采用七氟醚吸入和瑞芬太尼持续泵注。两组患者每隔40min追加顺式阿曲库铵0.05mg/kg。维持镇静指数在40~60之间,呼末二氧化碳在30~40mmHg之间。心率低于50次/分时给予阿托品0.5mg，收缩压低于100mmHg时酌情给予去甲肾上腺素或麻黄碱。手术结束前15min，试验组静脉给予酮咯酸氨丁三醇30mg和盐酸帕洛诺司琼0.25mg，对照组静脉给予地佐辛5mg和盐酸帕洛诺司琼30mg。两组均连接PCA进行术后镇痛（试验组：酮咯酸氨丁三醇3mg/kg+右美托咪定0.6μg/kg，对照组：酮咯酸氨丁三醇3mg/kg +地佐辛0.5mg/kg）。

8.试验流程图

VATS患者

年龄18~65岁

ASAⅠ~Ⅱ级

排除：（*n*=）

1、患者拒绝参加（*n*=）

2、具有排除标准其中任一项（*n*=）

3、其它（*n*=）

随机分组（*n*=）

对照组（*n*=）

1. 给予常规有阿片镇痛药物的全麻+胸椎旁阻滞（*n*=）
2. 未完成试验操作、标本采集或记录（原因）（*n*=）

试验组（*n*=）

1. 给予无阿片类镇痛药的全麻+胸椎旁阻滞（*n*=）
2. 未完成试验操作、标本采集或记录（原因）（*n*=）

随访（*n*=）

随访时剔除（原因）（*n*=）

随访（*n*=）

随访时剔除（原因）（*n*=）

纳入分析（*n*=）

分析时剔除（原因）（*n*=）

纳入分析（*n*=）

分析时剔除（原因）（*n*=）

9.记录指标：

①术中观察指标：在患者入室后给麻醉药前（T1）、给右美托咪定1μg/kg后10min(T2)、插管后(T3)、进胸（T4）、单肺通气30min（T5）、断气管（T6）、单肺通气结束（T7）、拔管后（T8）这几个时间点分别记录其脉搏氧饱和度、心率、平均动脉压及镇静指数等指标;在入室后给药前（t1）、手术开始1小时（t2）、手术开始2小时（t3）、术毕（t4）四个时间点分别抽取桡动脉血0.5ml行血气分析，记录这四个时间点的血糖值和乳酸值。术毕记录两组患者术中血管活性药使用情况、苏醒时间、拔管时间、苏醒评分。记录术中各种麻醉药、镇痛药的用量。记录胸椎旁阻滞后10~20min的阻滞平面。

②术后随访观察指标：术后1天（T1）、2天（T2）、3天（T3）、4天（T4）和5天（T5）的生命体征、安静及咳嗽时的VAS疼痛评分、阻滞平面、PCA的按压次数、镇痛补救措施，以及镇静指数等。记录术后排气时间、拔除引流管时间、以及术后恶心呕吐、皮肤瘙痒、腹痛、尿潴留、低氧血症、肺部并发症等不良反应发生情况、术后住院日。

③远期随访指标：术后1年回访患者，并记录患者的术后并发症情况、有无慢性疼痛、肿瘤复发情况和生存情况。

10.数据收集：

1. 我院胸腔镜肺手术患者一般术后需住院5~10天左右，故绝大多数患者可以完成面对面的术后随访；若患者提前出院则进行电话随访。
2. 数据管理：录入试验中采集的各项指标数据，采用双人录入。
3. 数据质量控制：数据监管人员不参与试验实施，独立于申办者且双方无利益冲突。数据监管人员负责录入数据的核查，稽查随机标号、分组、随访记录、数据收集。
4. 退出试验：患者中途如因任何原因出现以下任何一种或多种情况，均退出研究：不愿意参加或不愿继续参加此研究、拒绝随访、术后出现谵妄、病情加重或术后出血、转入ICU或PCA失败等影响试验开展的情况。
5. 结局指标：首要监测指标为术中镇静指数，血糖值，乳酸值，术后安静及咳嗽时的VAS疼痛评分、阻滞平面、PCA的按压次数、镇痛补救药剂量，以及安静及咳嗽时的镇静指数等。次要监测指标为术后排气时间、拔除引流管时间以及术后恶心呕吐、皮肤瘙痒、腹痛、尿潴留、低氧血症、肺部并发症等不良反应发生情况、术后住院日。

11.统计分析：

统计学处理：采用 SPSS21.0 统计学软件进行数据分析，计量资料以均数±标准差（‾x±SD）表示，采用独立样本t检验，计数资料用M（IQR）表示，采用秩和检验 。 *P* ＜0.05 为差异有统计学意义。

**三、**结果公布：

通过发表文章来报告研究结果。

四、补充内容：

1．受试者管理制度：

术前向受试者或其授权委托人提供知情同意书，告诉其随机分组可能存在的风险，充分考虑后决定是否参加试验。受试者有权在试验的任何阶段退出试验。受试者将会被随机分组，并不能知晓分组结果，在该患者随访试验结束后可告知。

2．不良反应管理制度：

本研究所涉及到的药物、器械、操作均为常规的全身麻醉所需要用到的，因此所涉及到的风险均为常规胸科手术全身麻醉所涉及到的风险。如胸椎旁阻滞可能出现的副作用有术中心率下降、低血压 ，B超引导胸椎旁穿刺能明显降低穿破胸膜、穿破血管的发生率（文献表明上述风险仅为2.6%~5%）；非甾体类镇痛药的应用可能增加消化道溃疡等风险。发生低心率、低血压时及时应用血管活性药；阻滞侧为术侧，术中镜下能观察到穿刺导致的气胸或出血，并及时行修补。

3. 术中常用药物

全身麻醉药：依托咪酯；七氟烷；右美托咪定

镇痛药：舒芬太尼；瑞芬太尼；酮咯酸氨丁三醇

肌松药：顺式阿曲库铵
