# Supplementary material for: Opioid-free anesthesia compared to opioid anesthesia for lung cancer patients undergoing video-assisted thoracoscopic surgery: A randomized controlled study
Source: PLoS One. 2021 Sep 23;16(9):e0257279. doi: 10.1371/journal.pone.0257279 (PMC8460000; doi:10.1371/journal.pone.0257279)
Supplement: S2 File — (DOCX) [file pone.0257279.s003.docx]

Statement

I confirm that the clinical trial protocol I have included as Supplementary Information is the version that was submitted to and approved by our ethics committee before the trial began.

**Opioid-free anesthesia in video-assisted thoracoscopic surgery lobectomy**

Solution version：2018-6-20 Version number 2.0

Participant：

| Professional title ` | Name | Division of labor |
| --- | --- | --- |
| Senior professional title | Xuelian Zhao | Study design |
|  | Jianfeng Fu | Quality control |
| Junior professional title | Yiwen Zhang | Enrolled patients, Anesthesia management and sample collection |
|  | Shasha Zhang | Postoperative follow-up |
| Graduate student | Nuoya Chen | Data collection and analysis |

Study leader： Pro Xuelian Zhao

Xuelian Zhao has final determination on tasks undertaken during study design, data collection, programmer management, data analysis interpretation, writing reports and publication of articles.

**Background:**

Adequate analgesia is an important part of general anesthesia which directly influences patients' postoperative quality of life and recovery from disease. Opioids was the most important analgesia in traditional general anesthesia because of the strong and definite analgesic action. Currently，with increasing attention to postoperative recovery, long term prognosis and enhanced recovery after surgery (ERAS), more and more researches have focused on side effects of opioids applied in general anesthesia and postoperative analgesia. The side effects contain respiratory complications, nausea, vomiting, inhibition of gastrointestinal peristalsis, social potential addiction, immunity inhibition, tumor metastasis, which can increase the perioperative respiratory risk, increase hospitalization expenses and prolong hospitalization time. The demand for analgesics decreased in minimally invasive surgery, so that low-dose opioid anesthesia and opioid free anesthesia could be implemented.

This study aimed to observe the effect of the general anesthesia regimen without opioids which could provide perioperative sufficient analgesia. The traditional multimodal analgesia regimens contains opioids, alpha 2-agonist, local anesthetic, ketamine and nonsteroidal antiinflammatory drugs (NSAIDs). Dexmedetomidine (DEX) is a potent and highly selective agonist of alpha-2 adrenoceptor, produces sedation, analgesia, smooth forgetting and anti-sympathetic nerve. DEX can improve the comfort of patients during the perioperative anesthesia, effectively relieve the stress reaction of patients during the perioperative period, maintain the stability of hemodynamics, and reduce the postoperative pain response. DEX has become a safe and effective adjuvant drug in clinical anesthesia and analgesia. NSAIDs was used for controlling mild and moderate pain. Nowadays, multi-mode analgesia regimens with different analgesic could obtain better analgesic effects and reduce side effects of drugs. Studies showed that postoperative analgesia combined with NSAIDs are effective and reduce adverse effects such as nausea, vomiting, and lethargy. Currently, regional nerve block has developed rapidly guided by ultrasound in anesthesia and analgesia. Our study intends to explore the feasibility of opioid-sparing general anesthesia with DEX, NSAIDs, sevoflurane plus thoracic paravertebral block in thoracoscopic surgery.

**Objection:**

Observation of the effect of opioid-free general anesthesia on patients scheduled for thoracoscopic lobectomy surgery and prognosis.

**Study design:**

1. Research location: operating room and thoracic surgical ward of Fourth Hospital of Hebei Medical University.
2. Sample size estimation: 50 cases per group were required for the study by using PASS11.0. Considering those lost cases of follow up, 110 subjects were needed.
3. Subjects source: inpatients in thoracic surgical department of Fourth Hospital of Hebei Medical University.
4. Secrecy regime: Findings from this project may be published in medical journals and individuals will be kept confidential. Personal information will not be disclosed unless required by relevant legal requirements. If necessary, government administration departments, hospital ethics committees, and other relevant researchers may consult your information as required.
5. Grouping method: Patients will be divided into two groups by Excel. Researchers will carry out the corresponding anesthesia plan according to group results and single blind procedure will be performed for follow-up physicians.
6. **Inclusion criteria, exclusion criteria, grouping and interventions:**

Inclusion criteria:

1. Patients(ASA Ⅰ/Ⅱ) of both sexes.
2. Aged from 18 to 65 years old.
3. Scheduled for VATS lobectomy.
4. Voluntarily to join the study and sign the informed consent form for the study.

Exclusion criteria:

1. Patients with cerebrovascular disease, digestive disease (e. g. peptic ulcer), dysfunction of liver, renal dysfunction, diabetes, hematological system disorders.
2. Patients with history of chronic pain, long time of taking opioids, NSAIDs or hormones.
3. Pregnant and lactating women.
4. Patients with mental disease who is unable to give a VAS score.
5. Patients who are allergic to local anesthetic or with regional nerve block contraindication.
6. Patients with moderate or severe cardiopulmonary dysfunction.
7. Patients with atropine contraindications.
8. The operation changes to open surgery.

Grouping： Patients will be divided into two groups，Group OFA with opioid-free anesthesia (n_1_ = 55) and Group OA with opioid-based anesthesia (n_2_ = 55).

Interventions:

Patients are routinely monitored after they entered operation room. In Group OFA, OFA with infusion dexmedetomidine and injection ketorolac tromethamine will be performed. Then ultrasound guided thoracic paravertebral block will be performed, and the block plane will be measured and recorded 10-20 minutes later. General anesthesia induction will be managed with etomidate and cisatracurium. Sevoflurane inhalation and dexmedetomidine continuous infusion will be used to maintain anesthesia. In Group OA, patients will receive ultrasound guided thoracic paravertebral block and then anesthesia induction will be completed with sufentanil, cisatracurium and etomidate. Inhalation sevoflurane and continuous infusion remifentanil will be used in the anesthesia maintenance stage.

1. **Anesthesia method:**

After the patient entered the operating room, ECG and SpO_2_ were routinely monitored, the arterial pressure was continuously measured by puncturing and catheterizing the left lateral flex artery and central venous pressure was continuously monitored by right internal jugular vein catheterization.

1. **Anesthetic induction：**

*Midazolam, atropine/penehyclidine hydrochloride will be administered before anesthesia (in our trail, midazolam and penehyclidine hydrochloride did not be administered before anesthesia induction, atropine was injected into venous in group OFA before anesthesia induction and HR <50 bpm). In Group OFA, OFA with infusion dexmedetomidine and injection ketorolac tromethamine will be performed. Then ultrasound guided thoracic paravertebral block will be performed, and the block plane will be measured and recorded 10~20 minutes later. General anesthesia induction will be completed by etomidate and cisatracurium. In Group OA, patients will receive ultrasound guided thoracic paravertebral block and then anesthesia induction will be completed with sufentanil, cisatracurium and etomidate. *Tracheal intubation will be performed with light rod or video laryngoscope (in our trail, a single lumen endotracheal tube was inserted into the main airway using a visual laryngoscope). PETCO_2_ and anesthesia gas monitoring will be performed in both groups.

1. **Anesthesia maintenance:**

Sevoflurane inhalation and dexmedetomidine continuous infusion will be used to maintain anesthesia in group OFA, and inhalation sevoflurane and continuous infusion remifentanil will be used in the anesthesia maintenance stage in group OA. *Cisatracurium (0.05mg/kg) will be injected per 40min in both groups (in the trail, muscle relaxation was maintained with boluses of cisatracurium (2-4 mg per 30 min) for individual difference). The sedation index will be maintained range from 40 to 60, PETCO_2_ will be adjusted from 30 mmHg to 40 mmHg. Atropine (0.5mg) will be given when the heart rate was lower than 50 bpm, *and noradrenaline or ephedrine will be given when the systolic pressure was lower than 100mmHg（in our trail, hypotension (MAP < 60 mmHg) was treated with norepinephrine）. 15 minutes before the end of the operation, in Group OFA, Ketorolac tromethamine (30mg) and palonosetron hydrochloride(0.25mg) will injected intravenously, while dezocine (5mg) and palonosetron hydrochloride(0.25mg) will be injected into Group OA. PCA (100ml) will be connected to patients of two groups for postoperative analgesia (Group OFA: ketorolac tromethamine 3mg/kg, dexmedetomidine 0.6μg/kg, Group OA: Ketorolac tromethamine 3mg/kg, dezocine 0.5mg/kg).

1. **Test flow chart**

patients

Age 18~65years

ASAⅠ/Ⅱ

Random allocation *n*=

exclusion：（*n*=）

1、patient refuse to participate（*n*=）

2、conform to exclusion criteria（n=）

3、other conditions（*n*=）

Group OA（*n*=）

1. general anesthesia with opioids analgesia +TPVB（*n*=）
2. Incomplete test operation or recording（reason）（*n*=）

Group OFA（*n*=）

1. general anesthesia without opioids analgesia +TPVB（*n*=）
2. Incomplete test operation or recording（reason）（*n*=）

Follow-up（*n*=）

Exclusion（reason）（*n*=）

Follow-up（*n*=）

Exclusion（reason）（*n*=）

Statistical analysis（*n*=）

Exclusion（reason）（*n*=）

Statistical analysis（*n*=）

Exclusion（reason）（*n*=）

1. **Record indicator：**
2. Intraoperative observation indicators：

SpO_2_, HR, MAP and sedation index will be recorded at before anesthesia (T1), 10 minutes after lording dexmedetomidine(1μg/kg) infusion (T2), after intubation (T3), after opening intercostal (T4), 30 minutes after the beginning of one-lung ventilation (T5), the time point of cutting off the bronchus (T6), the end of one-lung ventilation (T7), and after extubation (T8). 0.5ml of radial artery blood will be extracted for blood gas analysis at four time points : before anesthesia (T1), 1 hour after the beginning of the operation (T2), 2 hours after the beginning of the operation (T3), and after extubation (T4), and blood glucose levels and lactate values will be recorded at these four time points. The consumption of sevoflurane, recovery time, extubation time and awakening score will be recorded. The consumption of anesthetics and the consumption of analgesics should also be recorded. The block plane will be measured at 10~20 minutes after thoracic paravertebral block.

1. Postoperative follow-up observation indicators：

HR, MAP, VAS scores at resting, VAS scores on cough, number of segments of block plane, PCA pressing time, consumption of rescue analgesic and sedation index will be recorded from postoperative 1^st^ day to postoperative 5^th^ day. Postoperative exhaust time, drainage tube removal time, postoperative nausea and vomiting, skin itching, abdominal pain, urinary retention, hypoxemia, pulmonary complications, and other adverse reactions, as well as postoperative hospital stay will be observed.

1. Long-term follow-up indicators：

Patients will be revisited one year after surgery, and postoperative complications, chronic pain, tumor recurrence and survival will be followed up.

1. **Data collection:**
2. The patients need to be hospitalized for 5-10 days after surgery, so patients can complete face-to-face postoperative follow-up; Telephone follow-up will be conducted if the patient was discharged early.
3. Data management: Two person will input the data collected in the experiment.
4. Data quality control: Data supervisors are not involved in the implementation of the trial, and they are independent of the sponsor, and have no conflict of interest. Data supervisors are responsible for checking the input data, checking random label, grouping, follow-up records and data collection.
5. Quit the trial: If one or more of the following conditions occur, the patients will quit the study, such as refusing to participate in or continue to participate in this study, rejecting cooperate with the follow-up, experiencing postoperative delirium, meeting postoperative bleeding, transferring to ICU and having PCA fault.
6. Endpoint： SpO_2_, HR, MAP, sedation index, blood glucose levels, lactate values VAS scores, block plane, PCA pressing times, consumption of rescue analgesic at different time points are primary endpoints. Postoperative exhaust time, drainage tube removal time, postoperative nausea and vomiting, skin itching, abdominal pain, urinary retention, hypoxemia, pulmonary complications and other adverse reactions, as well as postoperative hospital stay are secondary endpoints.

**Statistical method:**

SPSS21.0 statistical software will be used for data analysis. Measurement data will be expressed as mean ± standard deviation, independent sample T-test will be used. Count data will be expressed as M (IQR), rank sum test will be used. *P* < 0.05 will be considered statistically significant.

**Research results published:**

The results will be published in journals.

**Supplementary description:**

1. Patient Management system:

Informed consent is provided to the subjects or their authorized clients before the surgery to inform them of the possible risks of randomization and to decide whether to participate in the trial after full consideration. Subjects are entitled to withdraw from the study at any stage of the study. Subjects will be randomly assigned and will not be informed of the outcome until the patient has finished the follow-up trial.

1. Adverse reaction management system:

The drugs, devices and operations involved in this study are required by conventional general anesthesia, so the risks involved are all those related to conventional general anesthesia for thoracic surgery. For example, the possible side effects of thoracic paravertebral blockade include bradycardia and hypotension. Ultrasound-guided thoracic paravertebral puncture can significantly reduce the incidence of puncture of pleura or blood vessels (the literature shows that without the ultrasound, the above risk is about 2.6%~5%). The blocking side is the operative side, so that pneumothorax or bleeding caused by puncture can be observed during the operation, and timely repair can be performed. The use of NSAIDs may increase the risk of gastrointestinal ulcer. In case of bradycardia and hypotension, vasoactive drugs will be applied in time.

1. Main commonly used drugs in the study

Sedative drug: Etomidate, Sevoflurane, Dexmedetomidine.

Analgesics: Sufentanil, Remifentanil, Etorolac tromethamine.

Muscle relaxant: Cisatracurium.

* the deviations from this study protocol in the Methods section of your manuscript
