# Supplementary material for: Opioid-free anesthesia compared to opioid anesthesia for lung cancer patients undergoing video-assisted thoracoscopic surgery: A randomized controlled study
Source: PLoS One. 2021 Sep 23;16(9):e0257279. doi: 10.1371/journal.pone.0257279 (PMC8460000; doi:10.1371/journal.pone.0257279)
Supplement: S3 File — (DOCX) [file pone.0257279.s004.docx]

| **Protocol changes** | **Reason for changes** | **Notation** |
| --- | --- | --- |
| The pain threshold index (PTI) is determined to be the only primary outcome in our study, and the pain index (PI) is not recorded. | Our study aims to compare the intraoperative analgesic effect between the OFA and the OA.  PI can be only used to objectively monitor the degree of pain in conscious patients. So, our paper does not have data on PI. | Explained separately in method and discussion of the manuscript. |
| Serum potassium concentration was not recorded as the secondary outcome. | We did not record intraoperative blood potassium values, because some patients were detected in hypokalemia before the operation, and received intravenous infusion potassium chloride. | Explained separately in method and discussion of the manuscript. |
| Cancel the medication before induction of anesthesia. | Because midazolam is associated with postoperative delirium, midazolam is no longer used before anesthesia in patients undergoing thoracic surgery in our hospital. | The eligible patient was informed that they aren’t received any medication before anesthesia induction and signed a written informed consent form after obtaining the consent of the participant. |
| The registration content was revised on July 1, 2020, and it was approved on July 5, 2020. | The original protocol only said to detect arterial blood gas, and we clarified that the specific items of blood gas detection include PH, PaO2, lactic acid and blood glucose. |  |

**Protocol Changes**
