# Supplementary material for: Opioid-free anesthesia compared to opioid anesthesia for lung cancer patients undergoing video-assisted thoracoscopic surgery: A randomized controlled study
Source: PLoS One. 2021 Sep 23;16(9):e0257279. doi: 10.1371/journal.pone.0257279 (PMC8460000; doi:10.1371/journal.pone.0257279)
Supplement: Attachment — Submitted filename: Comments_PONE-D-20-27554_R4.pdf [file pone.0257279.s009.pdf]

PLOS ONE

Opioid-free anesthesia compared to opioid anesthesia for lung cancer patients undergoing video-assisted thoracoscopic surgery: a randomized controlled study

--Manuscript Draft--

**Manuscript Number:**	PONE-D-20-27554R4
**Article Type:**	Clinical Trial
**Full Title:**	Opioid-free anesthesia compared to opioid anesthesia for lung cancer patients undergoing video-assisted thoracoscopic surgery: a randomized controlled study
**Short Title:**	Equal analgesic index and lower sedation index with opioid-free anesthesia compared with opioid anesthesia
**Corresponding Author:**	Xuelian Zhao<br>Hebei Medical University Fourth Affiliated Hospital and Hebei Provincial Tumor Hospital<br>Shijiazhuang, CHINA
**Keywords:**	Analgesia, Dexmedetomidine; Thoracic Surgery, Video-Assisted; Analgesics, Opioid; Pain Management, Blood glucose.
**Abstract:**	Background<br>Reducing intra-operative opioid consumption benefits patients by decreasing postoperative opioid-related adverse events.We assessed whether opioid-free anesthesia would provide effective analgesia-antinociception monitored by analgesia index in video-assisted thoracoscopic surgery.<br>Methods<br>Ninety-seven patients (ASA I-II, 18-65 years old, BMI <30 kg m−2) scheduled to undergo video-assisted thoracoscopic surgery under general anesthesia. Patients were randomly allocated into two groups to receive opioid-free anesthesia (group OFA) with dexmedetomidine, sevoflurane plus thoracic paravertebral blockade or opioid-based anesthesia (group OA) with remifentanil, sevoflurane, and thoracic paravertebral blockade. The primary outcome variable was pain intensity during the operation, assessed by the depth of analgesia using the pain threshold index with the multifunction combination monitor HXD-I. Secondary outcomes were depth of sedation monitoring by wavelet index and blood glucose concentration achieved from blood gas.<br>Results<br>Intraoperative pain threshold index readings were not significantly different between group OFA and group OA from arriving operation room to extubation (P = 0.86), while the brain wavelet index readings in group OFA were notably lower than those in group OA from before general anesthesia induction to recovery of double lungs ventilation (P <0.001) by multivariate test of repeated measurement ANOVA. After beginning of operation, the blood glucose levels in group OFA ascend by 20% compared with baseline blood glucose values (P < 0.001). The recovery time and extubation time in group OFA were significantly longer than those in group OA (P <0.007).<br>Conclusions<br>This study showed that our OFA regimen achieved equally effective intraoperative pain threshold index compared to OA in video-assisted thoracoscopic surgery. Depth of sedation was significantly lower and blood glucose levels were higher with OFA.
**Order of Authors:**	Guangquan An<br>Yiwen Zhang<br>Nuoya Chen<br>Jianfeng Fu<br>Bingsha Zhao<br>Xuelian Zhao
**Opposed Reviewers:**	
**Response to Reviewers:**	1. Comments. You should insert authors contributions (see PLOS One guidelines)<br>Author response: Thank you for your suggestion. As suggested by reviewer, we have

Powered by Editorial Manager® and ProduXion Manager® from Aries Systems Corporation

inserted author contributions in title page and submitted online.

2. Comments. I suggest to use a structured abstract (not exceeding 300 words)
Author response: We are grateful for the suggestion. To be more clearly and in accordance with the comment, we have converted the abstract into a structured abstract, see abstract with yellow labels.

3. Comments. Abstract. You should cite the EEG monitoring system in the abstract.
Author response: We agree with your comment. We have added the EEG monitoring system in the abstract, see Lines 14-15 with yellow labels.

4. Comments. Abstract. The TPVB abbreviation should be removed from the abstract.
Author response: Thank you for pointing this out. We have removed the TPVB abbreviation from the abstract, see Lines 11 with yellow labels.

5. Comments. Abstract, line 20-24. It should read: “This study showed that our OFA regimen achieved equally effective intraoperative pain threshold index compared to OA in video-assisted thoracoscopic surgery. Depth of sedation was significantly lower and blood glucose levels were higher with OFA.”
Author response: We do think these are excellent suggestions which makes the manuscript more rigorous and concise (Lines: 27-31, marked with yellow).

6. Comments. Introduction, line 39. It should read: “…respiratory depression [3], postoperative nausea and vomiting (PONV) [4] as well as postoperative delirium…”
Author response: Thank you for pointing this out. We have added ‘postoperative’ in lines 47 and marked with yellow.

7. Comments. Methods, trial design, line 85-88. This paragraph should be removed: “Because midazolam is associated with postoperative delirium, midazolam is no longer used before anesthesia in patients undergoing thoracic surgery in our hospital. The eligible patient was informed that they are not received midazolam and signed a written informed consent form after obtaining the consent of the participant.”
Author response: We deeply appreciate the reviewer’s suggestion. We have deleted the paragraph (Lines:95-98, yellow marked), and added the sentence “The eligible patient signed a written informed consent form after obtaining the consent of the participant.” (Lines:106-107, yellow marked).

8. Comments. Methods, allocation and blinding, line 97. You should not report results in the methods. It should read: “Patients were randomized in two groups (group OA and group OFA) using computer-generated random numbers (Excel® version 16)”
Author response: We do think this is good suggestion. We have corrected it, see line 110.

9. Comments. Methods, Anesthetic management protocol, page 8, line 140-141. It should read: “Sevoflurane was adjusted to maintain anesthesia. The use of midazolam was avoided. The patients were maintained on mechanical ventilation to keep the ….”
Author response: We do think this is excellent suggestion. We have made changes in relevant places in the manuscript marked with yellow, line 156-157.

10. Comments. Methods, outcomes, page 10, line 183. Exhaust time should be defined
Author response: Thank you for pointing this out. We have added the definition of exhaust time (Lines: 191, marked in yellow).

11. Comments. Methods, outcomes, page 10, line 185-190. The English of the paragraph should be improved.
Author response: We apologize for the language problems in the manuscript. Language presentation was improved with assistance from a native English speaker with appropriate research background (Lines: 193-203, marked with yellow).

12. Comments. Results, page 14, line 283-284. This sentence should be improved “No patient died, was transferred to ICU and respiratory dysfunction, and no patient was intubated again after the tracheal intubation was removed”. If judged correct, it should read: “No patient was transferred to the ICU, had respiratory insufficiency, need reintubation, or died during the follow-up”.

Author response: Our deepest gratitude goes to you for your careful work and thoughtful suggestions that have helped improve this paper substantially (Lines: 303-307, marked with yellow).

13. Comments. Discussion, first paragraph, line 290-295. It should read: “This prospective randomized study showed that our OFA regimen could provide equally adequate intraoperative analgesia-nociception balance in non-obese, 18-65 years old, ASA I-II patients compared to opioid based anesthesia guided by PTI monitoring in patients undergoing VATS. However, in group OFA, the intraoperative depth of sedation was deeper, and the blood glucose levels were higher, than those levels in patients with OA.”
Author response: Thank you for your precious comments and advice. The comment is all valuable and extremely helpful for revising and improving our paper. We have revised the manuscript accordingly, see line 313-318and yellow-marked.

14. Comments. Discussion, line 312-313. Ij judged correct, it should read: “…sevoflurane and TPVB were used in our study to replace opioids[26].”
Author response: Thank you for pointing this out. We have re-written this sentence (Lines: 340, yellow-marked).

15. Comments. Discussion, page 16, line 332. It should read: “…postoperatively improved pain relief (especially pain on coughing) up to 48 h….”
Author response: Thank you for pointing this out. We have rewritten this sentence (Lines: 359-361, marked with yellow).

16. Comments. Discussion, page 17, line 358. It should read: “Our results suggested that, in patients under non-opioid anesthesia…”
Author response: We do think this is good suggestion, which will make our paper more clarity. We have modified it (Lines: 389, marked with yellow).

17. Comments. Discussion, page 19, limitations paragraph, line 391. It should read: “Fourth, our study was a single-institution study, small sample size, and some protocol amendments were performed. More in details, no data on PI were reported since PI can be only used in conscious patients and the serum potassium concentration was not recorded, because some patients were given potassium supplementation, which potentially results in a selection bias. Fifth, we cannot perform an intention-to-treat analysis including the 3 patients excluded after randomization since we did not follow up these patients. Further studies will be needed to assess the long-term benefits of the avoidance of opioid use in lung cancer patients, as well as to assess the benefits of increasing survival.”
Author response: Thank you very much for revising this part. You have given me a more rigorous understanding of English wording and sentence making. According to your comment, we have rewritten this part, see line 425-437 and marked with yellow.

18. Comments. Conclusions, line 402-405. It should read: “OFA with dexmedetomidine and sevoflurane plus TPVB appears to be feasible for the management of intraoperative nociception in ASA I-II, 18-65 years old, non-obese patients undergoing VATS for lung cancer. However, a deeper sedation level was produced in OFA than in OA for the achievement of an optimal balance of analgesia-nociception.”
Author response: Thank you for underlining this deficiency. This section was revised and modified according to the suggestion (Lines:440-442, marked with yellow).

19. Comments. Manuscript. The language quality must be improved. We advise that you seek assistance from a colleague or have a professional editing service.
Author response: Thank you for your careful review. We are deeply sorry for the inconvenience they caused in your reading. The manuscript has been thoroughly revised and rewritten by a native English speaker, so we hope it can meet the journal’s standard. The modified details have been marked in yellow.
20. Comments. Please review your reference list to ensure that it is complete and correct. If you have cited papers that have been retracted, please include the rationale for doing so in the manuscript text, or remove these references and replace them with relevant current references. Any changes to the reference list should be mentioned in the rebuttal letter that accompanies your revised manuscript. If you need to cite a retracted article, indicate the article’s retracted status in the References list and also

	include a citation and full reference for the retraction notice.<br>Author response: Thank you for your reminder, we have re-checked the references, and the changes are marked in yellow.<br>Note: The authors of this article, Nuoya Chen and Bingsha Zhao, have moved to Hengshui People's Hospital and Tianjin Chest Hospital due to their job changes. We have revised their current addresses (see Title page),<br>Thank you for your careful review. We really appreciate your efforts in reviewing our manuscript. We wish good health to you, your family, and community. Your careful review has helped to make our study clearer and more comprehensive.
**Additional Information:**	
**Question**	**Response**
**Financial Disclosure**<br><br>Enter a financial disclosure statement that describes the sources of funding for the work included in this submission. Review the submission guidelines for detailed requirements. View published research articles from *PLOS ONE* for specific examples.<br><br>This statement is required for submission and **will appear in the published article** if the submission is accepted. Please make sure it is accurate.<br><br>**Unfunded studies**<br>Enter: *The author(s) received no specific funding for this work.*<br><br>**Funded studies**<br>Enter a statement with the following details:<br>• Initials of the authors who received each award<br>• Grant numbers awarded to each author<br>• The full name of each funder<br>• URL of each funder website<br>• Did the sponsors or funders play any role in the study design, data collection and analysis, decision to publish, or preparation of the manuscript?<br>• **NO** - Include this sentence at the end of your statement: *The funders had no role in study design, data collection and analysis, decision to publish, or preparation of the manuscript.*<br>• **YES** - Specify the role(s) played.<br><br>* typeset	The authors received no specific funding for this work.
**Competing Interests**<br><br>Use the instructions below to enter a	The authors have declared that no competing interests exist.

competing interest statement for this submission. On behalf of all authors, disclose any competing interests that could be perceived to bias this work—acknowledging all financial support and any other relevant financial or non-financial competing interests.<br><br>This statement **will appear in the published article** if the submission is accepted. Please make sure it is accurate. View published research articles from *PLOS ONE* for specific examples.<br><br>**NO authors have competing interests**<br><br>Enter: *The authors have declared that no competing interests exist.*<br><br>**Authors with competing interests**<br><br>Enter competing interest details beginning with this statement:<br><br>*I have read the journal's policy and the authors of this manuscript have the following competing interests: [insert competing interests here]*<br><br>* typeset	
**Ethics Statement**<br><br>Enter an ethics statement for this submission. This statement is required if the study involved:<br><br>• Human participants<br>• Human specimens or tissue<br>• Vertebrate animals or cephalopods<br>• Vertebrate embryos or tissues<br>• Field research<br><br>Write "N/A" if the submission does not require an ethics statement.<br><br>General guidance is provided below. Consult the submission guidelines for detailed instructions. **Make sure that all information entered here is included in the**	This prospective, randomized, parallel-group and single-center clinical trial was conducted from November 2018 to March 2019, at Fourth Hospital of Hebei Medical University China. The study complied with the Declaration of Helsinki and was approved by the local ethics committee (Fourth Hospital of Hebei Medical University Ethics Committee, Hebei, China, #2018049, Chairperson Prof Guiying Wang, on 20 Nov 2018).

**Methods section of the manuscript.**<br><br>**Format for specific study types**<br><br>**Human Subject Research (involving human participants and/or tissue)**<br>• Give the name of the institutional review board or ethics committee that approved the study<br>• Include the approval number and/or a statement indicating approval of this research<br>• Indicate the form of consent obtained (written/oral) or the reason that consent was not obtained (e.g. the data were analyzed anonymously)<br><br>**Animal Research (involving vertebrate animals, embryos or tissues)**<br>• Provide the name of the Institutional Animal Care and Use Committee (IACUC) or other relevant ethics board that reviewed the study protocol, and indicate whether they approved this research or granted a formal waiver of ethical approval<br>• Include an approval number if one was obtained<br>• If the study involved *non-human primates*, add *additional details* about animal welfare and steps taken to ameliorate suffering<br>• If anesthesia, euthanasia, or any kind of animal sacrifice is part of the study, include briefly which substances and/or methods were applied<br><br>**Field Research**<br><br>Include the following details if this study involves the collection of plant, animal, or other materials from a natural setting:<br>• Field permit number<br>• Name of the institution or relevant body that granted permission	
**Data Availability**<br><br>Authors are required to make all data underlying the findings described fully available, without restriction, and from the time of publication. PLOS allows rare exceptions to address legal and ethical concerns. See the PLOS Data Policy and FAQ for detailed information.	Yes - all data are fully available without restriction

Powered by Editorial Manager® and ProduXion Manager® from Aries Systems Corporation

A Data Availability Statement describing where the data can be found is required at submission. Your answers to this question constitute the Data Availability Statement and **will be published in the article**, if accepted.<br><br>**Important:** Stating 'data available on request from the author' is not sufficient. If your data are only available upon request, select 'No' for the first question and explain your exceptional situation in the text box.<br><br>Do the authors confirm that all data underlying the findings described in their manuscript are fully available without restriction?	
**Describe where the data may be found in full sentences. If you are copying our sample text, replace any instances of XXX with the appropriate details.**<br><br>• If the data are **held or will be held in a public repository**, include URLs, accession numbers or DOIs. If this information will only be available after acceptance, indicate this by ticking the box below. For example: *All XXX files are available from the XXX database (accession number(s) XXX, XXX.*).<br>• If the data are all contained **within the manuscript and/or Supporting Information files**, enter the following: *All relevant data are within the manuscript and its Supporting Information files.*<br>• If neither of these applies but you are able to provide **details of access elsewhere**, with or without limitations, please do so. For example:<br><br>*Data cannot be shared publicly because of [XXX]. Data are available from the XXX Institutional Data Access / Ethics Committee (contact via XXX) for researchers who meet the criteria for access to confidential data.*<br><br>*The data underlying the results*	All relevant data are within the manuscript.

Powered by Editorial Manager® and ProduXion Manager® from Aries Systems Corporation

presented in the study are available from (include the name of the third party and contact information or URL).<br>• This text is appropriate if the data are owned by a third party and authors do not have permission to share the data.<br><br>* typeset	
Additional data availability information:	Tick here if the URLs/accession numbers/DOIs will be available only after acceptance of the manuscript for publication so that we can ensure their inclusion before publication.

Powered by Editorial Manager® and ProduXion Manager® from Aries Systems Corporation

12 August 2020

Dear Editor:

I wish to submit an original clinical article for publication in PLOS ONE, titled “Equal effects of analgesia and deeper sedation with opioid-free anesthesia compared to opioid anesthesia for lung cancer patients undergoing video-assisted thoracoscopic surgery: a prospective, randomized, controlled study.”

It is reportedly beneficial to reduce opioid intra- and postoperative use to spare patients of postoperative opioid-related adverse events. In this randomized controlled trial, we assessed whether opioid-free anesthesia plus thoracic paravertebral blockade would provide effective hypnosis and pain relief monitoring with pain threshold index with a sample of 97 patients scheduled to undergo video-assisted thoracoscopic surgery under general anesthesia.
The patients in the experimental group received opioid-free anesthesia with dexmedetomidine and sevoflurane plus thoracic paravertebral blockade, and the patients in the control group received opioid-based anesthesia with remifentanil, sevoflurane plus thoracic paravertebral blockade.

We believe that our study makes a significant contribution to the literature and will be of interest to the readership of your journal because we demonstrated that our opioid-free anesthesia regimen achieved equally effective perioperative analgesia and greatly reduced the depth of sedation compared to opioid anesthesia in video-assisted thoracoscopic surgery.

Please consider, as potential referees, 1. Ashham Mansur (University Medical Center Goettingen, GERMANY, orcid.org/0000-0002-0322-2480); 2. Zheng-Liang Ma (Nanjing University Medical School Affiliated Nanjing Drum Tower Hospital, CHINA, orcid.org/0000-0002-5085-750X).

This manuscript has not been published or presented elsewhere in part or in entirety and is not under consideration by another journal. All study participants provided informed consent, and the study design was approved by the appropriate ethics review board. We have read and understood your journal’s policies, and we believe that neither the manuscript nor the study violates any of these.

The authors received no specific funding for this work.
The authors have declared that no competing interests exist.

Thank you for your consideration. I look forward to hearing from you.

Sincerely,
Xuelian Zhao
Department of Anesthesia, the Fourth hospital of Hebei Medical University
Postal address:12 Jiankang Road Shijiazhuang China 050011
Mobile: 86-15832120086
mail address: zhaoxuelian@hebmu.edu.cn

Manuscript

Click here to access/download;Manuscript;plos-manuscript-4.DOCX

Opioid-free anesthesia compared to opioid anesthesia for lung cancer patients undergoing video-assisted thoracoscopic surgery: a randomized controlled study

Guangquan An MD [1¶], Yiwen Zhang[2¶], Nuoya Chen[2#a], Jianfeng Fu MD[2], Bingsha Zhao[2#b], Xuelian Zhao MD PhD[2*].

[1] Department of Second Surgery, the Fourth hospital of Hebei Medical University, Shijiazhuang, Hebei, PR China

[2] Department of Anesthesia, the Fourth hospital of Hebei Medical University, Shijiazhuang, Hebei, PR China

[#a]Current address: Department of Anesthesia, Hengshui People's Hospital, Hebei, PR China

[#b]Current address: Department of Anesthesia, Tianjin Chest Hospital, Tianjin, PR China

* Corresponding author:

E-mail address: zhaoxuelian@hebmu.edu.cn （XL Z）

[¶] These authors contributed equally to this work.

Authors' Contributions

Conceptualization: YW Z, NY C and XL Z; Data Curation: NY Cand BS Z; Formal Analysis: YW Z; Investigation: GQ A and NY C ; Methodology: YW Z, NY C, JF F and XL Z; Project Administration: GQ A, ; Resources: GQ A, ; Software: YW Z and BS Z; Supervision: JF F and XL Z; Validation: JF F; Writing-Original Draft Preparation: GQ A, YW Z and XL Z; Writing Review &Editing: XL Z.

Abstract

Background

Reducing intra-operative opioid consumption benefits patients by decreasing postoperative opioid-related adverse events. We assessed whether opioid-free anesthesia would provide effective analgesia-antinociception monitored by analgesia index in video-assisted thoracoscopic surgery.

Methods

Ninety-seven patients (ASA I–II, 18-65 years old, BMI <30 kg m^{-2}) scheduled to undergo video-assisted thoracoscopic surgery under general anesthesia. Patients were randomly allocated into two groups to receive opioid-free anesthesia (group OFA) with dexmedetomidine, sevoflurane plus thoracic paravertebral blockade or opioid-based anesthesia (group OA) with remifentanil, sevoflurane, and thoracic paravertebral blockade. The primary outcome variable was pain intensity during the operation, assessed by the depth of analgesia using the pain threshold index with the multifunction combination monitor HXD- I. Secondary outcomes were depth of sedation monitoring by wavelet index and blood glucose concentration achieved from blood gas.

Results

Intraoperative pain threshold index readings were not significantly different between group OFA and group OA from arriving operation room to extubation (*P* = 0.86), while the brain wavelet index readings in group OFA were notably lower than those in group OA from before general anesthesia induction to recovery of double lungs ventilation (*P* <0.001) by multivariate test of repeated measurement ANOVA. After beginning of operation, the blood

glucose levels in group OFA ascend by 20% compared with baseline blood glucose values ($P < 0.001$). The recovery time and extubation time in group OFA were significantly longer than those in group OA ($P <0.007$).

Conclusions

This study showed that our OFA regimen achieved equally effective intraoperative pain threshold index compared to OA in video-assisted thoracoscopic surgery. Depth of sedation was significantly lower and blood glucose levels were higher with OFA.

Keywords: Analgesia, Dexmedetomidine; Thoracic Surgery, Video-Assisted; Analgesics, Opioid; Pain Management, Blood glucose.

TRIAL REGISTRATION: ChiCTR1800019479, Title: "Opioid-free anesthesia in video-assisted thoracoscopic surgery lobectomy".

Introduction

Pain management following video-assisted thoracoscopic surgery (VATS) is still a challenging issue associated with significant pain and a risk of developing chronic pain due to intercostal nerve compression and injuries that occur when instrumentation is used in the intercostal space [1]. Opioids are the most effective analgesics and are widely used in VATS with opioid based anesthesia (OA) to produce perioperative analgesia, hypnosis, and the inhibition of the sympathetic system. However, even short-term use of opioid analgesics during surgery can affect the patient's postoperative recovery. Due to impaired health and the patient ' s respiratory function, side effects associated with intraoperative opioid administration include hyperanalgesia [2], chronic postoperative pain, respiratory depression

[3], postoperative nausea and vomiting (PONV) [4] and postoperative delirium [5]. The possibility of higher risks of progression and metastasis of lung cancer cells treated with opioids has also been reported [6-9]. This explains the current trend of using non-opioid drugs as an alternative to the use of opioids for pain management during the perioperative period.

Recent studies have shown that opioid-free anesthesia (OFA) with multimode intraoperative opioid sparing strategy, such as locoregional analgesia, ketamine, clonidine, and dexmedetomidine, can provide better postoperative analgesia, as well as lower risks of PONV, and hypoxemia in laparoscopic cholecystectomy, breast cancer surgery, spine surgery, and bariatric surgery [10-13]. In a retrospective study, Bello and colleagues reported that OFA for patients undergoing thoracotomy appeared to be feasible and enhance postoperative pain relief with less cumulative ropivacaine consumption in the PCEA machine for 48 h after surgery, in addition to less postoperative morphine consumption and lower postoperative VAS scores [14]. In another retrospective study, the data from Guinot and colleagues showed that OFA in cardiac surgery was associated with lower morphine consumption, shorter intubation times and shorter ICU stays [15]. We hypothesized that the use of OFA in patients receiving VATS can achieve the goals of analgesia, hypnosis, hemodynamic stability, and the avoidance of the side effects of opioids. Few studies have reported pain management related to VATS with OFA and the continuous monitoring of the depth of analgesia during VATS.

~~Under deep general anesthesia, the patient was unconscious. The shift from opioid-based anesthesia to non-opioid anesthesia raises the problem of nociceptive monitoring; in other words, the monitoring of the pathophysiological response to anesthesia and surgical~~

stress [16]. A variety of analgesia monitors have been developed (based on physiological principles) to determine the depth of pain during surgery. The pain threshold index (PTI), which is a 0-100 noninvasive index calculated from changes in electroencephalographic (EEG) signals, has also been recently proposed to reflect the analgesia-nociception balance during general anesthesia (GA). This equipment can offer a continuous analgesia-nociception index reading in unconscious patients under GA [17, 18]. There are currently few trials in the literature that have investigated whether OFA provides a uniform balance of analgesia-nociception when compared to traditional opioid-anesthesia in VATS with PTI.

In this prospective randomized trial, we tested the main hypothesis that protocol-driven intraoperative OFA can provide effective intraoperative analgesia compared to the opioid-based GA with PTI.

Materials and Methods

Trial design

This prospective, randomized, parallel-group and single-center clinical trial was conducted from November 2018 to March 2019, at Fourth Hospital of Hebei Medical University China. The study complied with the Declaration of Helsinki and was approved by the local ethics committee (Fourth Hospital of Hebei Medical University Ethics Committee, Hebei, China, #2018049, Chairperson Prof Guiying Wang, on 20 Nov 2018). The study was registered on ClinicalTrials.gov (ChiCTR 1800019479).

Participant eligibility and consent

Inclusion criteria: Patients with ASA physical status I or II, aged 18-65 years who scheduled for elective thoracoscopic radical resection of lung cancer were recruited. Exclusion criteria: Patients who were pregnant or breastfeeding women; patients with allergy to any experimental drug or its excipients; patients who were prescribed β-blockers and HR <50 bpm; patients with body mass index (BMI) more than 30 kg m^{-2}; patients with central nervous system diseases (such as epilepsy, cerebral infarction, or cerebral hemorrhage history), history of chronic pain, alcohol, or drug abuse. The eligible patient signed a written informed consent form after obtaining the consent of the participant.

Allocation and blinding

Patients were randomized in two groups (group OA and group OFA) using computer-generated random numbers (Excel® version 16). The distribution results are sealed in opaque, sealed envelopes and kept by the research coordinator. On the day of operation, the research coordinator gave each patient an envelope to the anesthesiologist in the operation room. To facilitate the management of intraoperative anesthesia, anesthesiologists who did not participate in patient evaluation at any time are aware of this plan. In the operation room, the data collector who records various outcomes during general anesthesia knows the method of anesthesia. Patients, surgical staff, and postoperative evaluators were unaware of their groupings.

EEG measurement method

After the patient rested for 2 min, the forehead and the mastoid behind the ears were cleaned. The EEG electrodes of the multifunction combination monitor HXD-I

(Heilongjiang Huaxiang Technology Co., Ltd., Heilongjiang, China) were placed on 2 cm above the midpoint between the eyebrows, above the bilateral eyebrows, and at the bilateral mastoid process. Two channels of EEG data were recorded by HXD-I and were then implemented to reduce the complexity of the features through continuous and discrete wavelet transform. The repeatable and regular changes when pain present were extracted from the brain waves as the characteristic indicator of subjective pain.

The analgesic index PTI calculated according to changes in EEG signals, specifically reflects the tolerance of the cerebral cortex to painful stimuli. It is suitable for GA but not suitable for local anesthesia. The range is 0 - 100. A PTI < 39 may be a risk of overdose of analgesics; A PTI 40-60 is appropriate for intraoperative analgesia; A PTI >70 denotes insufficient analgesia and need to add analgesics. The PTI increases following painful stimuli and decreases with administration of analgesics.

The sedation index (wavelet index, WLI) ranging from 0-100 shows the depth of sedation with changes in EEG signals. WLI < 35 over sedation; WLI 35- 69 narcotization； WLI 70-89 light narcosis/ sleep; WLI > 90 wake fulness.

Anesthetic management protocol

On arrival at the operating room, the overnight fasting patients received standard monitoring, including ECG, invasive blood pressure, pulse oximetry, temperature. The intraoperative analgesic administration regimen was variable between the groups (Tab 1):

- For patients in the group OFA, a loading dose of dexmedetomidine (1 μg kg^{-1} over 10 min) and a bolus of atropine 0.5 mg were administered before GA induction. GA was maintained with dexmedetomidine infusion (0.5 μg $kg^{-1}h^{-1}$) terminated 40 min before the end of surgery. All patients received ketorolac 30 mg IV at GA induction.

- For patients in the group OA, a bolus of sufentanil (0.5 μg kg^{-1}) IV was administered for GA induction. For maintenance of GA, remifentanil (25 μg ml^{-1}, 8-20 ml h^{-1}) was continuously infused and discontinued before skin closure.

In both groups, GA was induced with a bolus of etomidate (0.2– 0.3mg kg^{-1}), followed by cisatracurium 0.2mg kg^{-1} for muscle relaxation. After that, a single-lumen endotracheal tube (ETT) and a bronchial blocker (BB) were inserted into airway in turn. Muscle relaxation was maintained with intermittent intravenous injection boluses of cisatracurium (2-4 mg per 30 min). Sevoflurane was adjusted to maintain anesthesia. The use of midazolam was avoided. The patients were maintained on mechanical ventilation to keep the $EtCO_2$ at 30–40 mmHg during two-lung ventilation (6-8 ml kg^{-1}, 12-14 tpm) and to keep the $EtCO_2$ at 30–50 mmHg during the period of one-lung ventilation (6 ml kg^{-1}, 14-16 tpm) with 100% oxygen inhalation. Intraoperative normothermia was maintained with forced air warming blankets positioned over the exposed parts of the body. Hypotension (MAP < 60 mmHg) was treated with norepinephrine, and bradycardia (HR < 45 bpm) was treated with atropine. Nitroglycerine was administered when MAP > 20% of the preinduction baseline values. The target of PTI was from 40 to 60 and that of WLI ranged from 40 to 60. The tracheal extubation was left to the clinician’s discretion when patients achieved a regular spontaneous breathing pattern.

All patients were placed in a lateral position with operation side up after anesthesia induction. After surgical disinfection, in-plane ultrasound-guided (Voluson i, GE USA) single-injecting thoracic paravertebral blockade (TPVB) at T5-6 was performed. Once the image of the disposable injection needle (18G 10 cm, Kangdelai China) was caught in a site between the internal intercostal membrane and the pleura, ropivacaine (0.5%, 15 ml) was administered over 30s. During injection, an anterior displacement of the pleura (downward

movement) was observed.

Table 1. Time chart of anesthetic management

Premedication	Loading (10 min)	GA Induction	GA Maintenance	GA Recovery
Group OFA	Dexmedetomidine<br>(1μg kg^{-1}, 4μg/ml)	Dexmedetomidine<br>(0.5 μg kg^{-1} h^{-1})<br>Ketorolac<br>(30 mg)<br>Etomidate<br>(0.2-0.3 mg kg^{-1})<br>Cisatracurium<br>(0.2 mg kg^{-1})	Dexmedetomidine *<br>(0.5 μg kg^{-1} h^{-1})<br>Sevoflurane #<br>(1%-3%)<br>Cisatracurium<br>(2-4 mg per 30 min)	Palonosetron<br>0.25 mg<br>Neostigmine<br>(up to 2 mg)<br>Atropine<br>(0.2-1 mg)
Group OA		Sufentanil<br>(0.5 μg kg^{-1})<br>Etomidate<br>(0.2-0.3 mg kg^{-1})<br>Cisatracurium<br>(0.2 mg kg^{-1})	Remifentanil #<br>(200-500 μg h^{-1})<br>Sevoflurane #<br>(1%-3 %)<br>Cisatracurium<br>(2 -4 mg per 30 min)	Namefene[19, 20]<br>0.5 mg<br>Palonosetron<br>(0.25 mg)<br>Neostigmine<br>(up to 2 mg)<br>Atropine<br>(0.2-1 mg)

* Infusion was stopped 40 min before end of surgery. # At end of surgery, inhalation and infusion were terminated.

GA: general anesthesia.

Outcomes

The primary outcome of this study was intraoperative PTI reading recorded at after entering the operating room (PTI0, baseline value), 10min after infusion of loading dose dexmedetomidine (PTI1), after intubation (PTI2), 30min after one-lung ventilation (PTI3), recovery of double lungs ventilation (PTI4), 5min after extubation in the operation room (PTI5). Secondary outcomes include WLI reading, MAP and HR at same timepoints as PTI, arterial partial pressure of oxygen, blood glucose concentration and lactic acid value from intraoperative blood gas at after entering the operating room (BGA0, baseline value), 1h after surgery begun (BGA1), 2h after surgery begun (BGA2), 5min after extubation in the

operation room (BGA3). Total consumption of anesthesia medications, exhaust time (the time from returning to the ward to the first exhaust), PONV, length of stay, pH and SpO_2 were measured and recorded.

In our trial registered with Clinical-Trials.gov, PI (pain index) was declared as primary outcome, and potassium concentration was recorded as secondary outcome. PI is a value indicating the degree of pain in awake subjects and is not suitable for patients under anesthesia. Therefore, ~~our paper has no data on PI.~~ Since some patients received intravenous potassium supplementation due to BGA0 blood gas detection of hypokalemia before induction of anesthesia, the intraoperative blood potassium values were not recorded.

Power analysis and sample size calculation

The sample size requirement was based on preliminary data from a previous pilot study of 15 patients undergoing VATS with OA which mean (*s*) PTI was 55.5 (10.4) after open chest. We assumed that OFA could achieve an equal PTI. The significance level is 0.05, the power is 0.9, the true difference is 6 and the standard deviation is 10. We calculated that each group should include 44 patients (PASS 11). Considering that the postoperative follow-up was carried out in the ward before discharge, the dropout rate was determined to be 10%, We recruited 50 patients in each group.

Statistical analysis

The categorical data were analyzed using the chi-squared test for independence or the Fisher's exact test. The quantitative data were analyzed using the unpaired Student's *t*-test for significance. If the data were not normally distributed as assessed by the Shapiro–Wilk test, the Wilcoxon rank-sum test was used. Descriptive parameters are represented as means (SD). Highly skewed quantitative data are presented as median with interquartile range (IQR).

Repeated measures analysis of variance and student t-test were used to compare PTI, WLI, MAP, HR at six time points and PaO_2, lactic acid levels and blood glucose concentration at four time points within and between groups. Statistical significance was set at $P < 0.05$. All statistical analyses were performed using the SPSS 17.0.

Results

A total of 222 patients who from November 2018 to March 2019 were assessed for eligibility, finally 100 patients were enrolled in this clinical investigation and were comparable in the two groups (Fig 1). one patient turned to open surgery, one peripheral venous catheter slid out during surgery, one patient needed re-exploration for postoperative intra-thoracic bleeding on postoperative day 1, and the remaining 97 patients were suitable for analysis (Fig 1). The two groups had similar characteristics at baseline (Tab 2).

Fig 1. Study protocol.

* Reasons for exclusion from the analysis: Group OFA: 1-converted to open lobetomy surgery. Group OA: 1-failure of PCA machine. 2-postopertive intra-thoracic bleeding requiring re-exploration on postoperative day 1.

Table 2. Clinical characteristics.

	Group OFA<br>n =49	Group OA<br>n =48	*P* –value
Gender (Female/Male)	27/22	29/19	0.68[#]
Age (IQR)	55 (47.5-60.5)	55 (50.3-59.3)	0.93[*]
Body Mass Index (BMI, IQR)	23.4 (21.3-25.5)	23.4 (22.2-26.4)	0.27[*]
Sites of VATS (Left/Right, n)	14/35	15/33	0.83[#]
Wedge resection/ Lobectomy/both: n	7/40/2	10/38/0	0.28[#]
Duration of anesthesia (SD, min)	151.3 (47.6)	160.8 (68.6)	0.44[§]
Duration of Surgery (SD, min)	137.1 (47.2)	144.6 (68.9)	0.54[§]
Fluid Infusion Volumes (IQR, ml)	1000 (1000-1250)	1100 (937.5-1263)	0.39*
Urinary volume （IQR, ml）	350 (250-500)	300 (225-500)	0.89*
Dexmedetomidine consumption (IQR, **μ**g)	140 (130-165)	—	
Remifentanil consumption (IQR, mg)	—	1.3 (1.0-1.6)	
End-expiratory sevoflurane concentration at end of surgery (IQR, %)	1.4 (1.2-1.7)	1.3 (1.2-1.4)	0.15*
Ephedrine consumption (IQR, mg)	0 (0-6.0)	0 (0-5.3)	0.47*
Norepinephrine consumption (IQR, **μ**g)	8 (0-46)	22 (0-85)	0.11*
Recovery time (SD, min)	13.2 (6.1)	9.5 (4.1)	< 0.001[§]
Extubation time (SD, min)	12.5 (5.7)	9.7 (4.1)	0.007[§]
Remove drainage tube time (IQR, day)	4 (3.5-6)	5 (4-6)	0.62*
Dizziness	2 (4.3%)	12 (25%)	0.005[#]
Postoperative nausea and vomiting	2 (4.3%)	20 (41.7%)	<0.001[#]
Exhaust time (IQR, day)	1 (1-1)	1 (1-2)	0.02[*]
Hospital Discharge time (95%CI, day)	7.2(4.2)	6.6(1.7)	0.66*

Data are presented as relative number of patients, mean ± standard deviation (SD), median (IQR). Times are shown in minutes.

[*] Wilcoxon rank-sum test; [#] Fisher's exact test; [§] Student's *t*-test.

Recovery time: time from intravenous injection of neostigmine and/or nalmefene to WLI >90; Extubation time: time from the completion of intravenous neostigmine and/or nalmefene to the removal of the tracheal tube; Exhaust time: the time from returning to the ward to the first breaking wind. If the duration is less than 24h, it is recorded 1 day; 24h-48h is 2 days, 48h-72h is 3 days, and so on.

The results of single factor (time) repeated measurement analysis showed that PTI value, WLI value, PaO_2, lactic acid level and blood glucose concentration of the two groups showed significant changes at different time points (P < 0.001). At the same time, the linear fit test of the trend of PTI, WLI, PaO_2, lactic acid and blood glucose under either opioid-free anesthesia or opioid-based anesthesia were also statistically significant (Tab 3 and 4, Fig 2 and 3, P <0.001).

Table 3. Summary of PTI and WLI with repeated measurement (mean, 95% CI)

	Group	PTI0	PTI1	PTI2	PTI3	PTI4	PTI5	*P*		
								Time	Group	Time*Group
PTI	OFA	91.8 (89.5-94.1)	88.2 (84.7-91.8)	61.9 (58.6-65.2)$^{\#}$	53.6 (50.0-57.3)$^{\#}$	59.7 (55.7-63.8)$^{\#}$	92.7 (90.8-94.6)	< 0.001*	0.86	0.62
	OA	91.3 (89.1-93.5)	88.8 (85.2-92.4)	63.0 (59.6-66.4)$^{\#}$	54.2 (50.1-57.8)$^{\#}$	60.6 (56.6-64.5)$^{\#}$	88.8 (84.7-92.9)			
WLI	OFA	96.3 (95.0-97.5)	82.7 (77.8-8.5)$^{\#\wedge}$	46.9 (42.8-51.1)$^{\#\wedge}$	47.8 (44.3-51.3)$^{\#\wedge}$	55.2 (51.4-59.0)$^{\#\wedge}$	88.4 (86.9-89.9)$^{\#}$	< 0.001*	< 0.001	< 0.001
	OA	95.4 (93.9-96.9)	90.9 (86.6-95.2)	54.5 (51.0-58.3)$^{\#}$	53.2 (50.6-55.8)$^{\#}$	59.5 (56.0-63.0)$^{\#}$	88.6 (87.2-89.9)			
MAP	OFA	106 (102-109)	107 (101-111)	115 (110-120)$^{\wedge}$	88 (84 (92)$^{\#}$	89 (85-93)$^{\#}$	98 (94-102)$^{\wedge}$	< 0.001*	0.89	<0.001
	OA	104 (101-108)	102 (99-106)	105 (101-110)	86 (83-89)$^{\#}$	93 (90-97)$^{\#}$	109 (105-113)			
HR	OFA	73 (69-76)	71 (68-74)	78 (74-81)	73 (70-76)	70 (66-73)	74 (71-76)$^{\wedge}$	< 0.001	0.004	0.001
	OA	76 (72-80)	75 (71-78)	82 (77-86)	74 (71-77)	74 (71-76)	87 (82-92)$^{\#}$			

PTI0: baseline value, PTI1: 10min after infusion of loading does dexmedetomidine, PTI2: after intubation, PTI3: 30min after one-lung ventilation, PTI4: recovery of double lungs ventilation, PTI5: 5 min after extubation.

WLI: wavelet index; PTI: pain threshold index; MAP: mean arterial pressure; HR: heart rate.

*: Linear fitting test (time) $P < 0.05$; $^{\#}$: Compared to PTI0 $P < 0.05$; $^{\wedge}$: Compared between two groups $P < 0.05$.

Table 4. Summary of PaO_2, Lactic and Blood glucose with repeated measurement (mean, 95% CI)

	Group	BGA0	BGA1	BGA2	BGA3	*P*		
						Time	Group	Time*Group
PaO_2	OFA	81.7 (78.8-84.5)	166.9 (137.4-196.3)$^{\#}$	282.6 (243.2-322.0)$^{\#\wedge}$	84.5 (78.3-90.7)	< 0.001*	0.39	0.01
	OA	81.6 (79.1-84.1)	190.6 (157.6-223.7)$^{\#}$	223.9 (188.6-259.2)$^{\#}$	82.5 (72.2-92.8)			
Lactic	OFA	1.2 (1.1-1.3)	1.2 (1.2-1.3)	1.2 (1.1-1.3)	1.4 (1.3-1.5)$^{\#}$	< 0.001*	0.71	0.06
	OA	1.3 (1.2-1.4)	1.2 (1.1-1.3)	1.2 (1.1-1.3)	1.5 (1.4-1.7)$^{\#}$			
Blood Glucose	OFA	5.6 (5.4-5.7)	6.7 (6.4-7.0)$^{\#\wedge}$	6.6 (6.4-6.8)$^{\#\wedge}$	6.3 (6.1-6.6)$^{\#}$	< 0.001*	0.004	< 0.001
	OA	5.8 (5.6-5.9)	5.8 (5.6-6.0)	5.9 (5.7-6.1)	6.2 (5.9-6.5)$^{\#}$			

BGA0: baseline value, BGA1: 1h after surgery begun, BGA2: 2h after surgery begun, BGA3: 5 min after extubation.

PaO_2: arterial oxygen partial pressure.

*: Linear fitting test (time) $P < 0.05$; $^{\#}$: Compared to BGA0 $P < 0.05$; $^{\wedge}$: Compared between two groups $P < 0.05$.

Fig 2. Changes in wavelet index, pain threshold index, mean arterial pressure and heart rate (mean ±SD) between two groups.

WLI: wavelet index; PTI: pain threshold index; MAP: mean arterial pressure; HR: heart rate. PTI0: baseline value, PTI1: 10min after dexmedetomidine infusion (1μg kg^{-1}), PTI2: after intubation, PTI3: 30min after one lung ventilation, PTI4: both lungs ventilation, PTI5: 5 min after extubation. *: $p < 0.05$ between groups

Fig 3: Changes in arterial oxygen partial pressure, lactic and blood glucose with blood gas (mean±SD) between two groups.

BGA0: baseline value, BGA1: 1h after surgery begun, BGA2: 2h after surgery begun, BGA3: 5 min after extubation. PaO_2: arterial oxygen partial pressure. *: $p < 0.05$ between two groups.

PTI readings between two groups were not significantly different at six time-points during the surgery ($P > 0.05$, Fig 2). The WLI readings at PTI1, PTI2, PTI3 and PTI4 were statistically significant lower in group OFA than those in group OA (such as 46.7 at PTI2 in group OFA vs. 54.5 at PTI2 in group OA; difference, 9.5 [95% CI, 3 to 13]; $P < 0.001$), and there was also the interaction between grouping factors * time factors ($P < 0.001$).

The results of single factor (time) repeated measurement analysis showed that (Table 3, Fig 3) the blood glucose levels of patients without opioid anesthesia were significantly increased (20%) at BGA1, BGA2 and BGA3 ($P < 0.01$) which can be considered as blood glucose levels increased during the operation. There was no significant difference in blood glucose levels at different timepoints in group OA ($P > 0.05$). The results of multivariate repeated measure analysis of variance showed that the difference in blood glucose levels between the two anesthesia methods was statistically significant ($P < 0.001$).

The recovery time and extubation time of the OFA group were significantly prolonged ($P < 0.05$). There were not significantly difference in PaO_2, pH and SpO_2 between the two groups during operation. There was no local infections or hematomata at the puncture site 24 h after operation. During the follow-up period, no patient was transferred to the ICU, had respiratory insufficiency, need reintubation, or died. Acute cerebral infarction occurred in 1

case in group OA, and atrial fibrillation occurred in 1 case after operation. No patients in group OFA had hypoxia ($SpO_2 < 90\%$), hypotension or bradycardia requiring treatment.

Discussion

This prospective randomized study showed that compared with opioid anesthesia under the guidance of PTI monitoring in patients receiving VATS, our OFA program can provide the same sufficient intraoperatively for ASA Ⅰ-Ⅱ, 18-65-years-old, non-obese patients Analgesia-nociception balance. However, in the group OFA had deeper intraoperative sedation and higher blood glucose levels.

In the present study, a non-opioid anesthesia regimen was effective in controlling the balance of nociception–antinociception of patients undergoing VATS, with intraoperative pain management guided by the PTI rather than via hemodynamic indices. The PTI readings in both groups were similar at all the time points and effectively maintained the optimal range from 40 to 60 during surgery, which is thought to predict adequate analgesia to nociceptive stimuli. Compared with OA, for patients who are unconscious under general anesthesia, there are few reports on the efficacy of intraoperative monitoring of the nociception-anti-nociception of OFA; therefore, the effect of non-opioid anesthesia has been criticized [21]. PTI has become a new parameter to balance nociception and analgesia during surgery, emphasizes the adequacy of perioperative analgesia in unconscious patients [22-24]. OFA is a multimodal anesthesia associated with hypnotics, N-methyl-D-aspartate antagonists, local anesthetics, anti-inflammatory drugs, and α-2 agonists. Tripathy and colleagues [25] found that patients undergoing magnetic resonance mammography with axillary dissection for breast cancer achieved superior analgesia with opioid-free pectoral nerve block-based

anesthesia that involved dexmedetomidine, propofol, and isoflurane. Due to the fact that there is specific type of OFA protocol, dexmedetomidine, sevoflurane and TPVB were used in our study to replace opioids [26]. Dexmedetomidine is a highly, potent, selective α- adrenergic agonist with intrinsic analgesic properties that can be prolonged for at least 12 h after surgery. Due to its analgesic properties, it has been used as an opioid substitute in various surgical interventions [10, 27-29]. ~~According to a meta-analysis review,~~ ~~Grape and colleagues demonstrated~~ that intraoperative dexmedetomidine was superior to remifentanil and its pain improvement can last up to 24 hours after surgery [30]. A loading dose of dexmedetomidine (1 μg kg^{-1}) infusion before the induction of anesthesia usually exhibits a central anti-sympathetic effect that may deepen anesthesia and spare the dose of anesthetics, and it can provide an optimal balance between adequate postoperative analgesic properties and adverse effects in sensory nerve blocks [31]. Han and colleagues reported that the intraoperative administration of a loading dose of dexmedetomidine (1 μg kg^{-1}) combined with sevoflurane (compared to the use of propofol) led to a significant stability in blood pressure during the operative period [32]. When compared with systemic agents, regional blockade is most effective for controlling movement-evoked pain [33]. PVB not only cause dense somatic afferent blockades, but (due to the anatomy of the paravertebral space) it also completely blocks transmission within the sympathetic chain. TPVB can produce the same effective analgesia (compared to the thoracic-epidural-blocks) and can reduce the consumption of opioids without the negative events associated with epidural blocks [34]. In a prospective, randomized, blinded study, patients receiving VATS, the authors observed relief of pain (especially pain during coughing) after TPVB for up to 48 hours. In additionally, intravenous infusion of dexmedetomidine significantly increased the duration of intermuscular sulcus

brachial plexus block analgesia and reduced cumulative opioid consumption [35, 36].

The WLI is a similar EEG-derived parameter to the BIS and is used to monitor the depth of sedation under GA [22, 37, 38]. Our results showed that with similar PTI readings between the two groups, WLI readings in the OFA group showed a more apparent decrease (10%) than those readings in the OA group. In addition, dexmedetomidine produced a state resembling stage 2 NREM sleep in the EEG with delta and alpha ranges of activity and characteristic arousal sedation [39]. Xu and colleagues [37] reported that, after dexmedetomidine infusion (a loading dose of 0.75 μg kg^{-1} and a continuous infusion of 0.5 μg kg^{-1} h^{-1}), the calculated cutoff WLI readings ranged from 55.8-93.7. Yan and Feng reported that injection equivalent analgesia doses of sufentanil (0.2 μg kg^{-1}) and remifentanil (2 μg kg^{-1}) produced mildly decreased WLI readings from 84.7-98.6 and 91.4-99.0 respectively [40]. Zhang and colleagues reported that, in patients undergoing posterior lumbar interbody fusion surgery with inhalation sevoflurane ranging from 1.5 vol % to 4 vol %, the WLI readings at the incision were 46.7 and 60 at skin closure [41]. Gu and colleagues reported that due to less propofol consumption, as well as different action sites between dexmedetomidine and propofol, the BIS value for loss consciousness was higher in a loading dose of dexmedetomidine (1μg kg^{-1}) with propofol than with an infusion propofol alone [37]. In our study, the consumption of sevoflurane during surgery was not recorded; therefore, we were not sure whether the lower WLI in OFA is caused by the effects of sevoflurane that requires further research. Our results showed that deeper sedation in the non-opioid anesthesia regimen with dexmedetomidine and sevoflurane (compared to opioid-based anesthesia with remifentanil and sevoflurane) was statistically significant during the in period of surgery, and the WLI readings in both groups were maintained at normal values

ranging from 40-60.

Our results suggested that, in patients under non-opioid anesthesia, the intraoperative blood glucose values increased significantly until the end of surgery. The increase in plasma glucose concentration is one of the main features of the metabolic response to surgery and simultaneously demonstrates the effect of different anesthesia techniques on the stress response [42]. Dexmedetomidine attenuates the intraoperative surgical stress response by decreasing nociceptive transmission and by decreasing cortisol levels, thus leading to lower blood glucose levels [43, 44]. In contrast, a recent paper showed that α2A-adrenoreceptors, which are present on pancreatic β-cells, is an important regulator of blood glucose homeostasis, and the activation of these receptors inhibits insulin secretion [45]. Therefore, dexmedetomidine may cause hypoinsulinaemia and a resultant state of hyperglycemia. Ghimire and colleagues [46] reported that 0.4 μg kg^{-1} dexmedetomidine decreased plasma insulin levels and mildly increased plasma glucose concentrations (4%) in healthy fasting human subjects. Görges and colleagues reported that in children undergoing elective surgery with total intravenous anesthesia, elevations in blood glucose levels (20%) were observed 15 minutes after the induction of anesthesia[47]. Additionally, sevoflurane anesthesia has been shown to increase plasma glucose levels by impairing insulin secretion, inducing insulin resistance, and reducing glucose uptake and utilization [48, 49]. The effects of sevoflurane combined with dexmedetomidine on blood glucose levels and insulin sensitivity in general anesthesia have not been reported yet. In our study, the blood glucose value was determined with the use of arterial blood gases to avoid tissue uptake and use glucose. Our results showed that the lactic acid levels were no significant difference between the two groups at the time points. The reasons for the increase in blood glucose concentrations in patients under

nonopioid anesthesia are that insulin secretion is inhibited by dexmedetomidine and sevoflurane and/or nonopioid anesthesia could not effectively control the stress response. These possible mechanisms requiring future studies.

This study had several limitations. First, intraoperative anesthesiologists ~~cannot turn a blind eye to interventions~~. Second, in our study, single-lumen bronchial intubation and BB (instead of a dual-lumen endotracheal tube) were used to reduce the stress response to intubation and extubation. Third, it is impossible to accurately calculate the amount of sevoflurane consumed during the operation; therefore, the end-tidal sevoflurane concentrations of the two group at the end of operation was compared. Considering that sevoflurane may affect the intraoperative sedation index and analgesia index, we began to calculate the consumption of sevoflurane at different times during the operation in follow-up experiments. Fourth, our research is a study a single-institution, with a small sample size, and some more detailed modifications have been made to some programs. There are no data reports on PI, since PI can only be used in conscious patients and the serum potassium concentration is not recorded, because some patients are supplemented with potassium, which may lead to selection bias. Fifth, we cannot perform an intention-to-treat analysis including the 3 patients excluded after randomization since we did not follow up these patients. Further studies will be needed to assess the long-term benefits of the avoidance of opioid use in lung cancer patients, as well as to assess the benefits of increasing survival.

Conclusions

OFA with dexmedetomidine and sevoflurane plus TPVB seems to be feasible for the management of intraoperative nociception in ASA Ⅰ-Ⅱ, 18-65 years old, non-obese patients

undergoing VATS for lung cancer. However, a deeper sedation level was produced in OFA than in OA for the achievement of an optimal balance of analgesia-nociception. We also identified significant elevation in blood glucose levels after the beginning of operation with opioid-free anesthesia. The patients under opioid anesthesia had slight changes in intraoperative blood levels. Further studies are required to evaluate the sedation depth of OFA with WLI as the primary outcome and to observe blood glucose levels in diabetic patients who accept OFA.

Acknowledgements

The authors would like to thank the operating room teams of the Fourth Hospital of Hebei University, including nurses and surgeons, for their help in permitting the experimental protocol to take place.

Reference

1. Chen Z, Ali JM, Xu H, Jiang L, Aresu G. Anesthesia and enhanced recovery in subxiphoid video-assisted thoracoscopic surgery. J Thorac Dis. 2018;10:6987-92.
2. Fletcher D, Martinez V. How can we prevent opioid induced hyperalgesia in surgical patients? Br J Anaesth. 2016;116:447-9.
3. Practice Guidelines for the Prevention, Detection, and Management of Respiratory Depression Associated with Neuraxial Opioid Administration: An Updated Report by the American Society of Anesthesiologists Task Force on Neuraxial Opioids and the American Society of Regional Anesthesia and Pain Medicine. Anesthesiology 2016;124:535-52.
4. Smith HS, Laufer A. Opioid induced nausea and vomiting. Eur J Pharmacol. 2014;722:67-78.
5. Harold S Minkowitz, Richard Scranton, Stephen K Gruschkus, Kathy Nipper-Johnson, Laura Menditto, Akash Dandappanavar. Development and Validation of a Risk Score to Identify Patients at High Risk for Opioid-Related Adverse Drug Events. J Manag Care Spec Pharm. 2014;20:948-58.

6. Cata JP, Keerty V, Keerty D, Feng L, Norman PH, Gottumukkala V, et al. A retrospective analysis of the effect of intraoperative opioid dose on cancer recurrence after non-small cell lung cancer resection. Cancer Med. 2014;3:900-8.

7. Singleton PA, Mirzapoiazova T, Hasina R, Salgia R, Moss J. Increased mu-opioid receptor expression in metastatic lung cancer. British journal of anaesthesia. 2014;113 Suppl 1:i103-8.

8. Maher DP, Wong W, White PF, McKenna R Jr, Rosner H, Shamloo B, et al. Association of increased postoperative opioid administration with non-small-cell lung cancer recurrence: a retrospective analysis. Br J Anaesth. 2014;113 Suppl 1:i88-94.

9. Oh TK, Jeon JH, Lee JM, Kim MS, Kim JH, Cho H, et al. Investigation of opioid use and long-term oncologic outcomes for non-small cell lung cancer patients treated with surgery. PLoS One. 2017;12:e0181672.

10. Bakan M, Umutoglu T, Topuz U, Uysal H, Bayram M, Kadioglu H, et al. Opioid-free total intravenous anesthesia with propofol, dexmedetomidine and lidocaine infusions for laparoscopic cholecystectomy: a prospective, randomized, double-blinded study. Braz J Anesthesiol. 2015;65:191-9.

11. Swagata Tripathy, Satyajit Rath, Suresh Agrawal, P Bhaskar Rao, A Panda, T S Mishra, and Sukdev Nayak. Opioid-free anesthesia for breast cancer surgery: An observational study. J Anaesthesiol Clin Pharmacol. 2018;34:35-40.

12. Ziemann-Gimmel P, Goldfarb AA, Koppman J, Marema RT. Opioid-free total intravenous anaesthesia reduces postoperative nausea and vomiting in bariatric surgery beyond triple prophylaxis. Br J Anaesth. 2014;112:906-11.

13. Kim DJ, Bengali R, Anderson T A. Opioid-free anesthesia using continuous dexmedetomidine and lidocaine infusions in spine surgery. Korean J Anesthesiol. 2017;70:652-3.

14. Bello M, Oger S, Bedon-Carte S, Vielstadte C, Leo F, Zaouter C, et al. Effect of opioid-free anaesthesia on postoperative epidural ropivacaine requirement after thoracic surgery: A retrospective unmatched case-control study. Anaesth Crit Care Pain Med. 2019;38:499-505.

15. Guinot P G, Spitz A, Berthoud V, Ellouze O, Missaoui A, Constandache T, et al. Effect of opioid-free anaesthesia on post-operative period in cardiac surgery: a retrospective matched case-control study. BMC Anesthesiol. 2019;19:136.

16. Lavand'homme P, Estebe JP. Opioid-free anesthesia: a different regard to anesthesia practice. Curr Opin Anaesthesiol. 2018;31:556-61.

17. An JX, Wang Y, Cope DK, Williams JP. Quantitative Evaluation of Pain with Pain Index Extracted from Electroencephalogram. Chin Med J (Engl). 2017;130:1926-31.

18. Hao Kong, Hong Zhang. Research progress of nociceptive stimulation and analgesic level monitoring during operation. Journal of Clinical Anesthesiology. 2020;36:612-5.

19. Chen Yingqi, Yue Yun, Qing Enming, Lin Duomao, Yang Baxian, Feng Yi, et al. Nalmefene antagonizes the effect of opioid postoperative respiratory depression-a multicenter, randomized, double-blind, positive drug control study. Chinese Journal of Anesthesiology. 2011;31:307-9.

20. Jia Zhen, Chen Yi, Zhang Linlin, Wang Xin, Zhao Yuan, Guo Suqian, et al. The effect of different low-dose nalmefene in preventing postoperative hyperalgesia induced by remifentanil. Chinese Journal of Anesthesiology. 2017;37:1159-62.

21. Frauenknecht J, Kirkham KR, Jacot-Guillarmod A, Albrecht E. Analgesic impact of intra-operative opioids vs. opioid-free anaesthesia: a systematic review and meta-analysis. Anaesthesia. 2019;74:651-62.

22. Wu L, Wang S, Wang Y, Zhang K, Bai J, Zheng J. Prediction of Hemodynamic Reactivity by Electroencephalographically Derived Pain Threshold Index in Children Undergoing General Anesthesia: A Prospective Observational Study. J Pain Res. 2019;12:3245-55.

23. Zhen Su, Lijun An, Yang Zhang, Penghua Chen. The clinical value of analgesia index in evaluating the degree of analgesia during general anesthesia. Chinese Journal of Applied Physiology. 2018;34:461-3.

24. Zhu Qingqing, Yue Wei, Jiang Jingnan, Zhang Miao, Yang Jianxin. Application of target-controlled infusion of remifentanil guided by pain threshold index in laparo-scopic subtotal gastrectomy. J Clin Anesthesiol. 2020;36:634-7

25. Tripathy S, Rath S, Agrawal S, Rao PB, Panda A, Mishra TS, et al. Opioid-free anesthesia for breast cancer surgery: An observational study. J Anaesthesiol Clin Pharmacol. 2018;34(1):35-40.

26. Dario Bugada, Luca F L, Patricia Lavand'homme. Opioid free anesthesia: evidence for short and long-term outcome. Minerva Anestesiol. 2021;87:230-7.

27. Kim DJ, Bengali R, Anderson TA. Opioid-free anesthesia using continuous dexmedetomidine and lidocaine infusions in spine surgery. Korean J Anesthesiol. 2017;70:652-3.

28. Kumar K, Kirksey MA, Duong S, Wu CL. A Review of Opioid-Sparing Modalities in Perioperative Pain Management: Methods to Decrease Opioid Use Postoperatively. Anesth Analg. 2017;125:1749-60.

29. Gaszynski T, Gaszynska E, Szewczyk T. Dexmedetomidine for awake intubation and an opioid-free general anesthesia in a superobese patient with suspected difficult intubation. Drug Des Devel Ther. 2014;8:909-12.

30. Grape S, Kirkham KR, Frauenknecht J, Albrecht E. Intra-operative analgesia with remifentanil vs. dexmedetomidine: a systematic review and meta-analysis with trial sequential analysis. Anaesthesia. 2019;74:793-800.

31. Motaghi E, Ghasemi Pirbaooti M, Bozorgi H, Eslami M, Rashidi M. Safety and Efficacy of Dexmedetomidine in Breast Surgeries: A Systematic Review and Meta-Analysis. J Perianesth Nurs. 2021;36:179-186.

32. Han Y, Han L, Dong M, Sun Q, Ding K, Zhang Z, et al. Comparison of a loading dose of dexmedetomidine combined with propofol or sevoflurane for hemodynamic changes during anesthesia maintenance: a prospective, randomized, double-blind, controlled clinical trial. BMC Anesthesiol. 2018;18:12.

33. Shanthanna H, Ladha KS, Kehlet H, Joshi GP. Perioperative Opioid Administration. Anesthesiology. 2021;134:645-59.

34. Agnoletti V GC, Piraccini E, Maitan S, Corso MR, Buccioli M, Poletti V, et al. Efficacy and safety of thoracic paravertebral block for medical thoracoscopy. Br J Anaesth. 2011;106:916-7.

35. RyungA Kang, Ji Seon Jeong, Jae Chul Yoo, Ju Hyun Lee, Soo Joo Choi, Mi Sook Gwak, et al. Effective Dose of Intravenous Dexmedetomidine to Prolong the Analgesic Duration of Interscalene Brachial Plexus Block: A Single-Center, Prospective, Double-Blind, Randomized Controlled Trial. Reg Anesth Pain Med. 2018;43:488-95.

36. Abdallah FW, Dwyer T, Chan VWS, Niazi A. U, Ogilvie-Harris D. J, Oldfield S, et al. IV and Perineural Dexmedetomidine Similarly Prolong the Duration of Analgesia after Interscalene Brachial Plexus Block: A Randomized, Three-arm, Triple-masked, Placebo-controlled Trial. Anesthesiology. 2016;124:683-95.

37. Xu Li, LI Zhao, Tian Guogang. Evalution of wavelet index in monitoring sedation depth with dexmedetomidine under combined spinal epidural anesthesia. J Clin Anesthesiol. 2018;34:1202-4.

38. Chun Xia, Yue Yun. Comparative investigation of wavelet index and bispectral index in monitoring depth of anesthesia. J Clin Anesthesiol. 2010;26:836-9.

39. Yang Gu, Fan Yang, Yonghai Zhang, Junwei Zheng, Jie Wang, Bin Li, et al. The effects of different doses of dexmedetomidine on the requirements for propofol for loss of consciousness in

patients monitored via the bispectral index a double-blind, placebo-controlled trial. BMC Anesthesiol. 2020;20:96-102.

40. Qi Yan, Yi Feng. Differences in sedation level and adverse effects produced by equivalent analgesic doses of remifentanil, sufentanil and fentanyl. Chin J Anesthesiol. 2012;32:853-6.

41. Zhang JW, Lv ZG, Kong Y, Han CF, Wang BG. Wavelet and pain rating index for inhalation anesthesia: A randomized controlled trial. World J Clin Cases. 2020;8:5221-34.

42. Desborough JP. The stress response to trauma and surgery. Br J Anaesth. 2000;85(1):109-17.

43. Li C S, Liu SF, Zhou Y, Lu X H. Effect of Dexmedetomidine on Perioperative Stress and Postoperative Pain in Patients With Radical Resection of Esophageal Cancer Under Combined Thoracoscope and Laparoscope. Zhonghua Yi Xue Za Zhi. 2018;98:3778-83.

44. Swarnendu Dey, Meenakshi Kumar. Comparison of pretreatment with dexmedetomidine with midazolam for prevention of etomidate-induced myoclonus and attenuation of stress response at intubation: A randomized controlled study. J Anaesthesiol Clin Pharmacol 2018;34:94-8.

45. Fagerholm V, Haaparanta M, Scheinin M. alpha2-adrenoceptor regulation of blood glucose homeostasis. Basic Clin Pharmacol Toxicol. 2011;108:365-70.

46. Ghimire LV, Muszkat M, Sofowora GG, Scheinin M, Wood AJ, Stein CM, et al. Variation in the alpha(2A) adrenoceptor gene and the effect of dexmedetomidine on plasma insulin and glucose. Pharmacogenet Genomics. 2013;23:479-86.

47. Görges M, Poznikoff AK, West NC, Brodie SM, Brant RF, Whyte SD. Effects of Dexmedetomidine on Blood Glucose and Serum Potassium Levels in Children Undergoing General Anesthesia: A Secondary Analysis of Safety Endpoints During a Randomized Controlled Trial. Anesthesia & Analgesia. 2019;129:1093-9.

48. O. Y. COK, Z. Ozkose, H. Pasaoglu, S. Yardim. Glucose response during craniotomy propofol-remifentanil versus isoflurane-remifentanil. Minerva Anestesiol 2011;77:1141-8.

49. Katsuya Tanaka, Takashi Kawano, Takehito Tomino, Hiroaki Kawano, Tsuyoshi Okada, Shuzo Oshita, et al. Mechanisms of Impaired Glucose Tolerance and Insulin Secretion during Isoflurane Anesthesia. Anesthesiology. 2009;111:1044-51.

Supporting information

S1 File. CONSORT checklist

S2 File. Ethic Document Comments

S3 File. Ethic Document Comments-English

S4 File. Informed Consent- Original

S5 File. Informed Consent-English

S6 File. Study Protocol-English

S7 File. Study Protocol-Original

S8 File. Language Editing Certificate

Figure
Click here to access/download;Figure;Figure 1 - CONSORT Flow Diagram.doc

CONSORT 2010 Flow Diagram

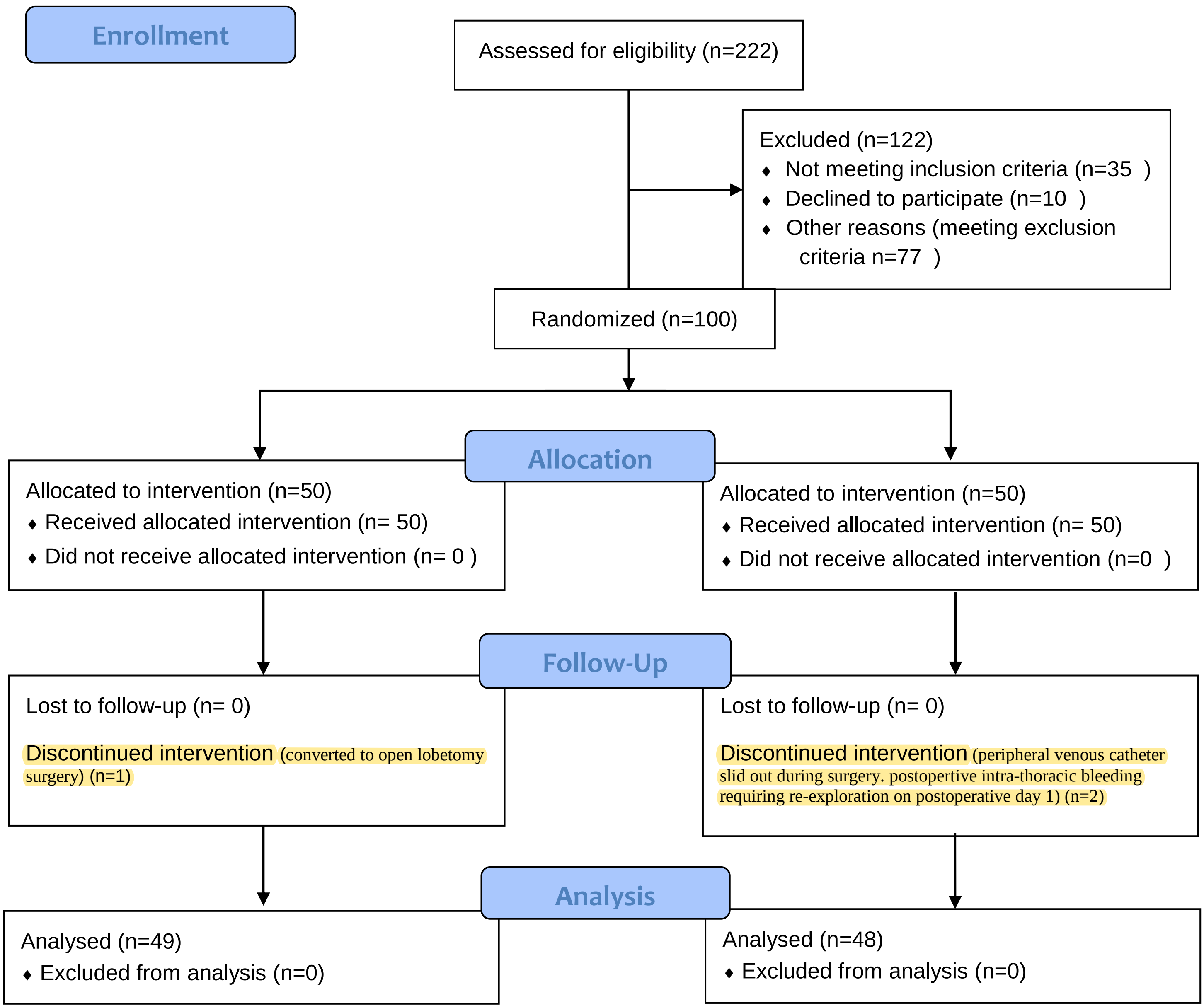

Fig 1: study protocol. * Reasons for exclusion from the analysis: Group OFA: 1-converted to open lobetomy surgery. Group OA: 1- peripheral venous catheter slid out during surgery. 2-postopertive intra-thoracic bleeding requiring re-exploration on postoperative day 1.

Figure Click here to access/download;Figure;Fig 2 PTI and WLI.docx

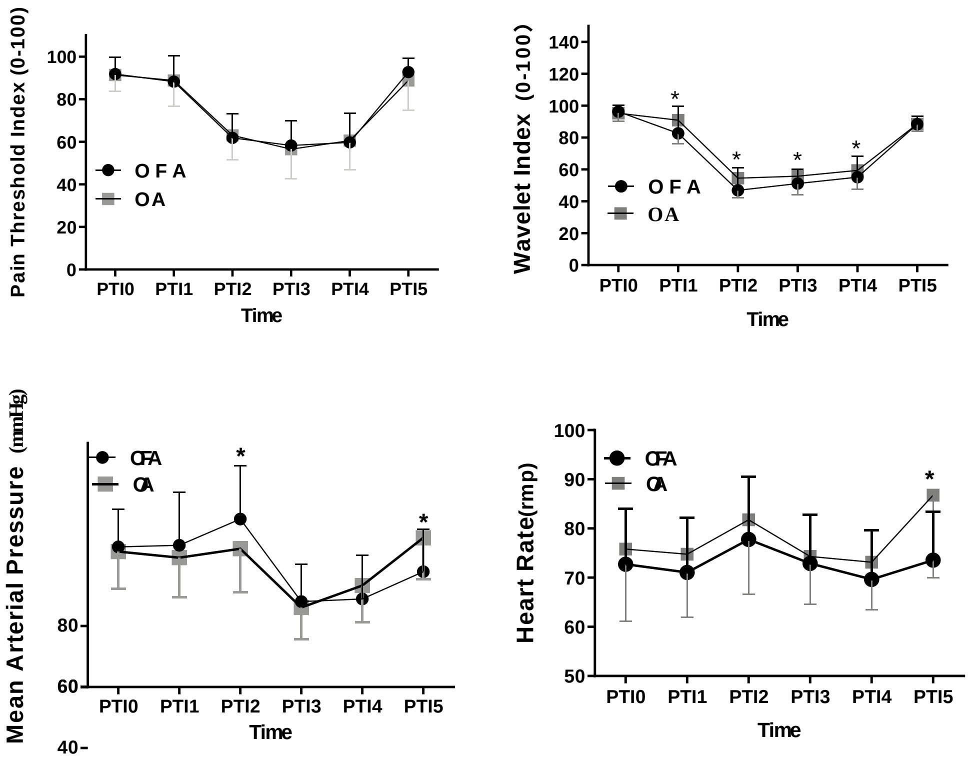

Figure 2 Changes in pain threshold index, wavelet index, mean arterial pressure and heart rate (mean ±SD) between two groups during intra-operative periods. PTI0: baseline value, PTI1: 10min after dexmedetomidine infusion (1μg kg^{-1}), PTI2: after intubation, PTI3: 30min after one lung ventilation, PTI4: both lungs ventilation, PTI5: 5 min after extubation. *: $p < 0.05$ between groups.

Figure Click here to access/download;Figure;Fig 3 Blood Gas.docx

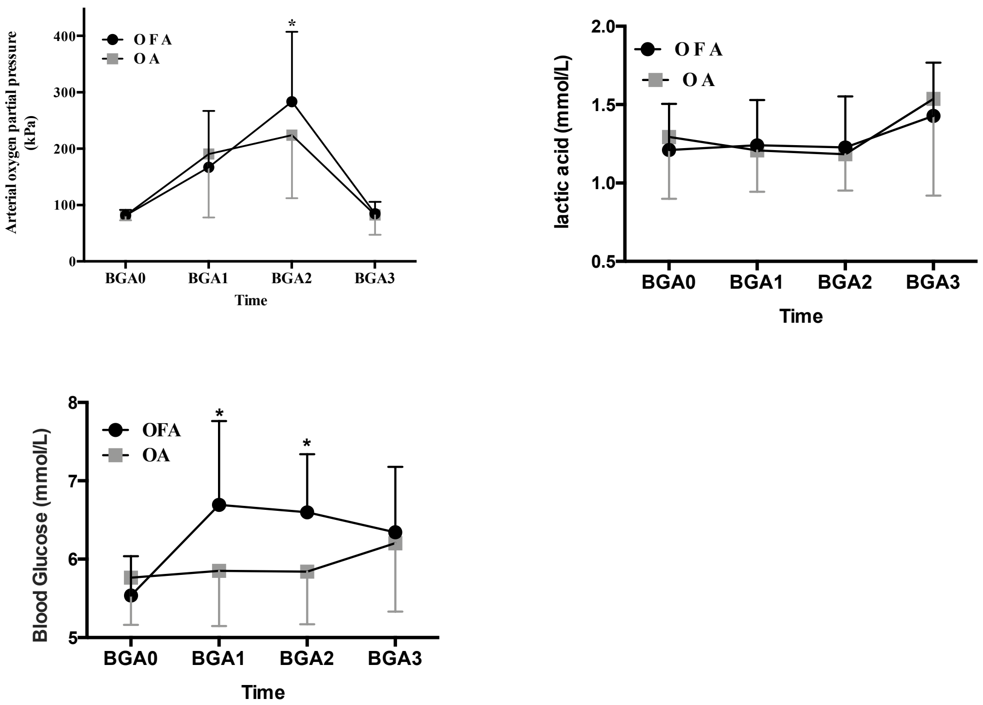

Figure 3: Changes in arterial oxygen partial pressure, lactic acid and blood glucose with blood gas (mean±SD) between two groups during surgery.BGA0: baseline value, BGA1: 1h after surgery begun, BGA2: 2h after surgery begun,BGA3: 5 min after extubation. *: $p < 0.05$ between two groups.

Figure Click here to access/download;Figure;Fig 2 PTI and WLI.docx

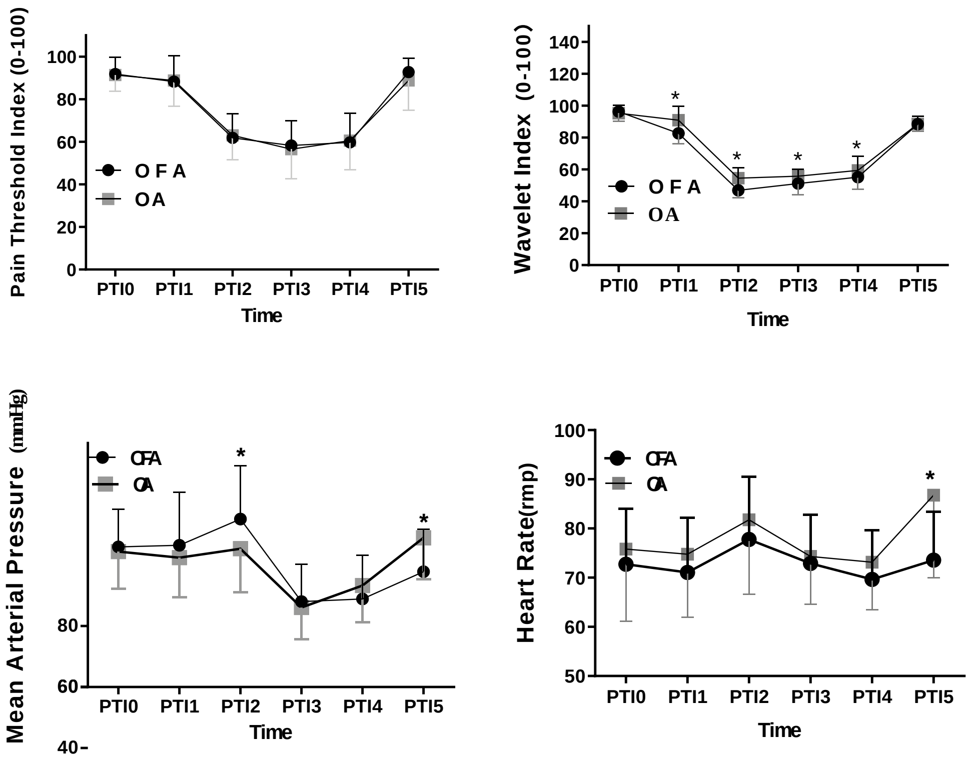

Figure 2 Changes in pain threshold index, wavelet index, mean arterial pressure and heart rate (mean ±SD) between two groups during intra-operative periods. PTI0: baseline value, PTI1: 10min after dexmedetomidine infusion (1μg kg^{-1}), PTI2: after intubation, PTI3: 30min after one lung ventilation, PTI4: both lungs ventilation, PTI5: 5 min after extubation. *: $p < 0.05$ between groups.

Figure Click here to access/download;Figure;Fig 3 Blood Gas.docx

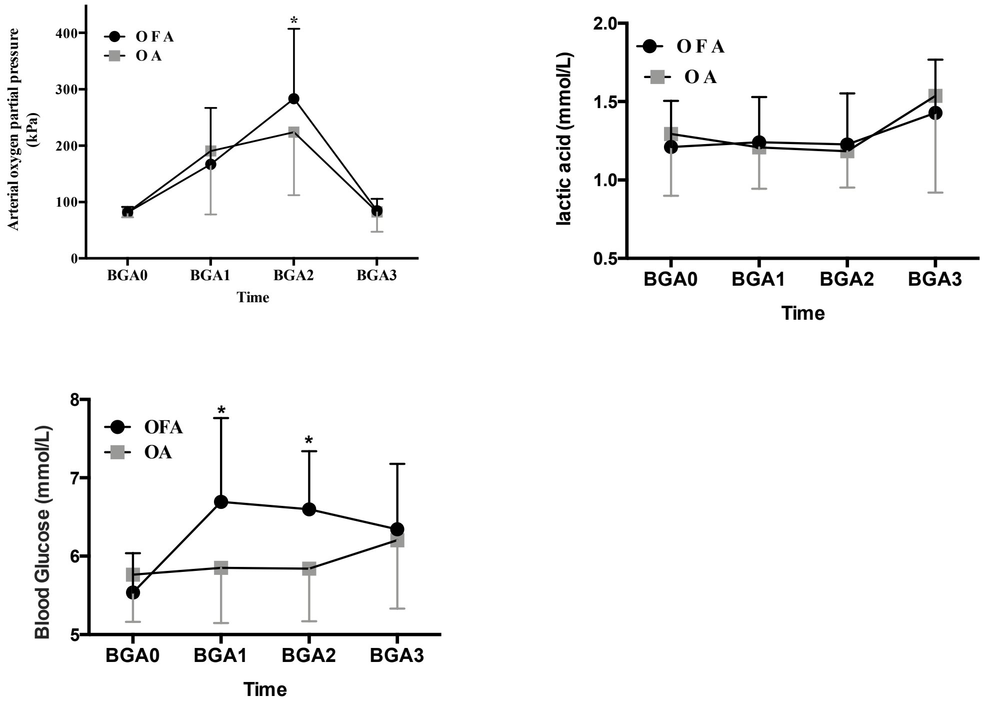

Figure 3: Changes in arterial oxygen partial pressure, lactic acid and blood glucose with blood gas (mean±SD) between two groups during surgery.BGA0: baseline value, BGA1: 1h after surgery begun, BGA2: 2h after surgery begun,BGA3: 5 min after extubation. *: $p < 0.05$ between two groups.

Click here to access/download
Supporting Information
Study Protocol-English.docx

Click here to access/download
Supporting Information
Study Protocol- Original.docx

Click here to access/download
Supporting Information
CONSORT 2010 Checklist.doc

Click here to access/download
Other
Ethic Document Comments.pdf

Click here to access/download
Other
Ethic Document-English.docx

Click here to access/download
Other
Informed Consent-Original.pdf

Click here to access/download
Other
Informed consent-English.docx

Click here to access/download
Other
Language Editing Certificate.pdf

Opioid-free anesthesia compared to opioid anesthesia for lung cancer patients undergoing video-assisted thoracoscopic surgery: a randomized controlled study

Guangquan An MD [1¶], Yiwen Zhang[2¶], Nuoya Chen[2#a], Jianfeng Fu MD[2], Bingsha Zhao[2#b], Xuelian Zhao MD PhD[2*].

[1] Department of Second Surgery, the Fourth hospital of Hebei Medical University, Shijiazhuang, Hebei, PR China

[2] Department of Anesthesia, the Fourth hospital of Hebei Medical University, Shijiazhuang, Hebei, PR China

[#a] Current address: Department of Anesthesia, ~~the Second Affiliated Hospital of Third Military Medical University , Chongqing , PR China~~ Hengshui People's Hospital, Hebei, PR China

[#b] Current address: Department of Anesthesia, Tianjin Chest Hospital, Tianjin, PR China

* Corresponding author:

E-mail address: zhaoxuelian@hebmu.edu.cn （XL Z）

[¶] These authors contributed equally to this work.

Authors' Contributions

Conceptualization: YW Z, NY C and XL Z; Data Curation: NY Cand BS Z; Formal Analysis: YW Z; Investigation: GQ A and NY C ; Methodology: YW Z, NY C, JF F and XL Z; Project Administration: GQ A, ; Resources: GQ A, ; Software: YW Z and BS Z; Supervision: JF F and XL Z; Validation: JF F; Writing-Original Draft Preparation: GQ A, YW Z and XL Z; Writing Review &Editing: XL Z.

Abstract

Background

Reducing intra-operative opioid consumption benefits patients by decreasing postoperative opioid-related adverse events. We assessed whether opioid-free anesthesia would provide effective analgesia-antinociception monitored by analgesia index in video-assisted thoracoscopic surgery.

Methods

Ninety-seven patients (ASA Ⅰ-Ⅱ, 18-65 years old, BMI <30 kg m^{-2}) scheduled to undergo video-assisted thoracoscopic surgery under general anesthesia. Patients were randomly allocated into two groups to receive opioid-free anesthesia (group OFA) with dexmedetomidine, sevoflurane plus thoracic paravertebral blockade ~~(TPVB)~~ or opioid-based anesthesia (group OA) with remifentanil, sevoflurane, and thoracic paravertebral blockade. The primary outcome variable was pain intensity during the operation, assessed by the depth of analgesia using the pain threshold index with the multifunction combination monitor HXD-Ⅰ. Secondary outcomes were depth of sedation monitoring by wavelet index and blood glucose concentration achieved from blood gas.

Results

Intraoperative pain threshold index readings were not significantly different between group OFA and group OA from arriving operation room to extubation (*P* = 0.86), while the brain wavelet index readings in group OFA were notably lower than those in group OA from before general anesthesia induction to recovery of double lungs ventilation (*P* <0.001) by multivariate test of repeated measurement ANOVA. After beginning of operation, the blood

glucose levels in group OFA ascend by 20% compared with baseline blood glucose values (*P* < 0.001). The recovery time and extubation time in group OFA were significantly longer than those in group OA (*P* <0.007).

Conclusions

This study showed that our ~~opioid-free anesthesia~~ OFA regimen achieved equally effective intraoperative ~~analgesia~~ pain threshold index compared to ~~opioid anesthesia~~ OA in video-assisted thoracoscopic surgery. ~~, while with opioid-free anesthesia,~~ ~~d~~Depth of sedation was significantly lower and blood glucose levels were higher ~~than those~~ with ~~opioid anesthesia~~ OFA.

Keywords: Analgesia, Dexmedetomidine; Thoracic Surgery, Video-Assisted; Analgesics, Opioid; Pain Management, Blood glucose.

TRIAL REGISTRATION: ChiCTR1800019479, Title: "Opioid-free anesthesia in video-assisted thoracoscopic surgery lobectomy".

Introduction

Pain management following video-assisted thoracoscopic surgery (VATS) is still ~~continues to be~~ a challenging issue associated with significant pain and a risk of developing chronic pain due to intercostal nerve compression and injuries that occur when instrumentation is used in the intercostal space [1]. Opioids are the most effective analgesics and are widely used in VATS with opioid based anesthesia (OA) to produce perioperative analgesia, hypnosis, and the inhibition of the sympathetic system. However, even short-term use of opioid analgesics during surgery can affect the patient's postoperative recovery. Due

to impaired health and the patient's respiratory function, s~~S~~ide effects ~~that are related to~~ associated with intraoperative opioid administration include hyperanalgesia [2], chronic postoperative pain, respiratory depression [3], postoperative nausea and vomiting (PONV) [4] ~~as well as~~ and postoperative delirium [5]~~, due to the compromised health status and impaired respiratory function of the patients~~. The possibility of higher risks of progression and metastasis of lung cancer cells treated with opioids has also been reported [6-9]. This explains the current trend ~~in~~ of using non-opioid drugs as an alternative to the use of opioids for pain management during the perioperative period.

Recent studies have shown that opioid-free anesthesia (OFA) with multimode intraoperative opioid sparing strategy, such as locoregional analgesia, ketamine, clonidine, and dexmedetomidine, can provide~~s~~ better postoperative analgesia, as well as lower risks of PONV, and hypoxemia in laparoscopic cholecystectomy, breast cancer surgery, spine surgery, and bariatric surgery [10-13]. In a retrospective study, Bello and colleagues reported that OFA for patients undergoing ~~open thoracic surgery~~ thoracotomy appeared to be feasible and enhance~~ds~~ postoperative pain relief with less cumulative ropivacaine consumption in the PCEA machine for 48 h after surgery, in addition to less postoperative morphine consumption and lower postoperative ~~pain~~ VAS scores [14]. In another retrospective study, the data from Guinot and colleagues showed that OFA in cardiac surgery was associated with lower morphine consumption, shorter intubation times and shorter ICU stays [15]. We hypothesized that the use of OFA in patients receiving VATS can achieve the goals of ~~the objectives of~~ analgesia, hypnosis, hemodynamic stability, and the avoidance of the side effects~~ of opioids can be achieved with OFA in patients undergoing VATS~~. Few studies have reported ~~on VATS-related~~ pain management related to VATS with OFA and the ~~constant~~ continuous monitoring

of the depth of analgesia during VATS.

Under deep general anesthesia, the patient was unconscious. The shift from opioid-based anesthesia to non-opioid anesthesia raises the problem of nociceptive monitoring; in other words, the monitoring of the pathophysiological response to anesthesia and surgical stress [16]. A variety of analgesia monitors have been developed (based on physiological principles) to determine the depth of pain during surgery. The pain threshold index (PTI), which is a 0-100 noninvasive index calculated from changes in electroencephalographic (EEG) signals, has also been recently proposed to reflect the analgesia-nociception balance during general anesthesia (GA). This equipment can offer a continuous analgesia-nociception index reading in unconscious patients under GA [17, 18]. There are currently few trials in the literature that have investigated whether OFA provides a uniform balance of analgesia-nociception when compared to traditional opioid-anesthesia in VATS with PTI.

In this prospective~~,~~ randomized trial, we tested the ~~primary~~ main hypothesis that protocol-driven intraoperative OFA can provide eff~~icacious~~ective intraoperative analgesia compared to the opioid-based GA with PTI~~-monitoring~~.

Materials and Methods

Trial design

This prospective, randomized, parallel-group and single-center clinical trial was conducted from November 2018 to March 2019, at Fourth Hospital of Hebei Medical University China. The study complied with the Declaration of Helsinki and was approved by the local ethics committee (Fourth Hospital of Hebei Medical University Ethics Committee, Hebei, China, #2018049, Chairperson Prof Guiying Wang, on 20 Nov 2018). The study was

registered on ClinicalTrials.gov (ChiCTR 1800019479).

Participant eligibility and consent

Because midazolam is associated with postoperative delirium, midazolam is no longer used before anesthesia in patients undergoing thoracic surgery in our hospital. The eligible patient was informed that they are not received midazolam and signed a written informed consent form after obtaining the consent of the participant. Inclusion criteria: Patients with ASA physical status I or II, aged 18-65 years who scheduled for elective thoracoscopic radical resection of lung cancer were recruited. The eExclusion criteria: included Patients who were pregnant or breastfeeding women,; patients with allergy to one of the drugs used for anesthesia, any experimental drug or its excipients; patients who were chronically treated with prescribed β-blockers and HR <50 bpm,; patients with body mass index (BMI) ≥ more than 30 kg m^{-2},; patients with central nervous system diseases (such as epilepsy, cerebral infarction, or cerebral hemorrhage history), history of chronic pain, alcohol, or drug abuse. The eligible patient signed a written informed consent form after obtaining the consent of the participant.

Allocation and blinding

100 pPatients were randomized in two groups (group OA and group OFA) using computer-generated random numbers (Excel® version 16). The allocation distribution results were are sealed in opaque, sealed envelopes that were and kept with by the research coordinator. On the day of surgery operation, the research coordinator gave each patient an handed one envelope per patient to the anesthesiologist in the operation room. In order tTo

facilitate the management of intraoperative anesthesia ~~management~~, ~~the~~ anesthesiologists who did not participate in ~~the assessment of the~~ patient~~s~~ evaluation at any time ~~were~~ are aware of this~~e protocol~~ plan. In the operation room, the data collector who records various outcomes during general anesthesia knows the method of anesthesia. Patients, surgical staff~~s~~, and postoperative evaluators were unaware of their groupings ~~assessors were blinded to their group assignment~~.

EEG measurement method

After the patient rested for 2 min, the forehead and the mastoid behind the ears were cleaned. The EEG electrodes of the multifunction combination monitor HXD‐I (Heilongjiang Huaxiang Technology Co., Ltd., Heilongjiang, China) were placed on 2 cm above the midpoint between the eyebrows, above the bilateral eyebrows, and at the bilateral mastoid process. Two channels of EEG data were recorded by HXD-I and were then implemented to reduce the complexity of the features through continuous and discrete wavelet transform. The repeatable and regular changes when pain present were extracted from the brain waves as the characteristic indicator of subjective pain.

The analgesic index PTI calculated ~~from~~ according to changes in EEG signals, specifically reflects the tolerance of the cerebral cortex to painful stimul~~iation~~. ~~and~~ It is suitable for GA~~,~~ but not suitable for local anesthesia.~~,~~ ~~and~~ The range~~s from~~ is 0 ~~to~~ -100. A PTI < 39 may be a risk of overdose of analgesics; A PTI 40-60 is appropriate for intraoperative analgesia; A PTI >70 denotes insufficient analgesia and need to add analgesics. The PTI increases following painful stimuli and decreases with administration of analgesics.

The sedation index (wavelet index, WLI) ranging from 0-100 shows the depth of

sedation with changes in EEG signals. WLI < 35 over sedation; WLI 35- 69 narcotization ； WLI 70-89 light narcosis/ sleep; WLI > 90 wake fulness.

Anesthetic management protocol

On arrival at the operating room, the overnight fasting patients ~~were applied with~~ received standard monitoring, ~~consist~~including ~~of~~ ECG, invasive blood pressure, pulse oximetry, temperature. The intraoperative analgesic administration regimen was variable between the groups (Tab 1):

- For patients in the group OFA, a loading dose of dexmedetomidine (1 μg kg^{-1} over 10 min) and a bolus of atropine 0.5 mg were administered before GA induction. GA was maintained with dexmedetomidine infusion (0.5 μg kg^{-1}h^{-1}) terminated 40 min before the end of surgery. All patients received ketorolac 30 mg IV at GA induction.
- For patients in the group OA, a bolus of sufentanil (0.5 μg kg^{-1}) IV was administered for GA induction. For maintenance of GA, remifentanil (25 μg ml^{-1}, 8-20 ml h^{-1}) was continuously infused ~~(25 μg ml^{-1}, 8-20 ml h^{-1})~~ and discontinued before skin closure.

In both groups, GA was induced with a bolus of etomidate (0.2– 0.3mg kg^{-1}), followed by cisatracurium 0.2mg kg^{-1} for muscle relaxation. ~~Then~~ After that, a single-lumen endotracheal tube (ETT) and a bronchial blocker (BB) were inserted into airway in turn. Muscle relaxation was maintained with intermittent intravenous injection boluses of cisatracurium (2-4 mg per 30 min). Sevoflurane was adjusted to maintain anesthesia. The use of midazolam was avoided. The patients were maintained on mechanical ventilation to keep the $EtCO_2$ at 30–40 mmHg during two-lung ventilation (6-8 ml kg^{-1}, 12-14 tpm) and to keep the $EtCO_2$ at 30–50 mmHg during the period of one-lung ventilation (6 ml kg^{-1}, 14-16 tpm) with 100% oxygen inhalation. Intraoperative normothermia was maintained with forced air

warming blankets positioned over the exposed parts of the body. Hypotension (MAP < 60 mmHg) was treated with norepinephrine, and bradycardia (HR < 45 bpm) was treated with atropine. Nitroglycerine was administered when MAP > 20% of the preinduction baseline values. The target of PTI was from 40 to 60 and that of WLI ranged from 40~~-~~ to 60. ~~At the end of surgery,~~ The tracheal extubation was left to the clinician's discretion when patients achieved a regular spontaneous breathing pattern.

All ~~the~~ patients were placed in a lateral position with operation side up after anesthesia induction. After surgical disinfection, in-plane ultrasound-guided (Voluson i, GE USA) single-injecting thoracic paravertebral blockade (TPVB) at T5-6 was performed. Once the image of the disposable injection needle (18G 10 cm, Kangdelai China) was caught in a site between the internal intercostal membrane and the pleura, ropivacaine (0.5%, 15 ml) was administered over 30s. During injection, an anterior displacement of the pleura (downward movement) was observed.

Table 1. Time chart of anesthetic management

Premedication	Loading (10 min)	GA Induction	GA Maintenance	GA Recovery
Group OFA	Dexmedetomidine (1μg kg^{-1}, 4μg/ml)	Dexmedetomidine (0.5 μg kg^{-1} h^{-1})<br><br>Ketorolac (30 mg)<br>Etomidate (0.2-0.3 mg kg^{-1})<br>Cisatracurium (0.2 mg kg^{-1})	Dexmedetomidine * (0.5 μg kg^{-1} h^{-1})<br><br>Sevoflurane # (1%-3%)<br>Cisatracurium (2-4 mg per 30 min)	Palonosetron 0.25 mg<br>Neostigmine (up to 2 mg)<br>Atropine (0.2-1 mg)

Group OA	Sufentanil (0.5 μg kg^{-1}) Etomidate (0.2-0.3 mg kg^{-1}) Cisatracurium (0.2 mg kg^{-1})	Remifentanil # (200-500 μg h^{-1}) Sevoflurane # (1%-3 %) Cisatracurium (2 -4 mg per 30 min)	Namefene[19, 20] 0.5 mg Palonosetron (0.25 mg) Neostigmine (up to 2 mg) Atropine (0.2-1 mg)

* Infusion was stopped 40 min before end of surgery. # At end of surgery, inhalation and infusion were terminated.

GA: general anesthesia.

Outcomes

The primary outcome of this study was intraoperative PTI reading recorded at after entering the operating room (PTI0, baseline value), 10min after infusion of loading dose dexmedetomidine (PTI1), after intubation (PTI2), 30min after one-lung ventilation (PTI3), recovery of double lungs ventilation (PTI4), 5min after extubation in the operation room (PTI5). Secondary outcomes include WLI reading, MAP and HR at same timepoints as PTI, arterial partial pressure of oxygen, blood glucose concentration and lactic acid value from intraoperative blood gas at after entering the operating room (BGA0, baseline value~~s~~), 1h after surgery begaun (BGA1), 2h after surgery begun (BGA2), 5min after extubation in the operation room (BGA3). Total consumption of anesthesia medications, exhaust time (the time from returning to the ward to the first exhaust), PONV, length of stay, pH and SpO_2 were measured and recorded.

~~When the~~ In our trial ~~was~~ registered with ~~on~~ Clinical-Trials.gov, ~~the~~ ~~comparison of~~ PI (pain index) ~~were~~ was declared as primary outcome, and potassium concentration ~~between the groups~~ ~~and~~ was recorded as secondary outcome~~, respectively~~. ~~PI can be only used to objectively monitor the degree of pain in conscious patients.~~ PI is a value indicating the

degree of pain in awake subjects and is not suitable for patients under anesthesia. Therefore ~~So~~, our paper ~~doe~~has no~~t have~~ data on PI. ~~We The intraoperative blood potassium values did were not recorded intraoperative blood potassium values, because some patients were detected in hypokalemia before the operation and received intravenous infusion potassium chloride before GA induction.~~ Since some patients received intravenous potassium supplementation due to BGA0 blood gas detection of hypokalemia before induction of anesthesia, the intraoperative blood potassium values were not recorded.

Power analysis and sample size calculation

The sample size requirement was based on preliminary data from a previous pilot study of 15 patients undergoing VATS with OA which mean (*s*) PTI was 55.5 (10.4) after open chest. We assumed that OFA could achieve an equal PTI. ~~With a~~ The significance level ~~of significance~~ is 0.05, ~~a~~ the power is 0.9, the true difference is 6 and the standard deviation is ~~SD~~ 10.~~,~~ ~~w~~We calculated that each group should include 44 patients (PASS 11). ~~Considering~~ Taking into account that the postoperative follow-up was carried out in the ward before discharge, and the withdrawal ~~dropout~~ rate was determined to be 10%, We recruited 50 patients ~~per~~ in each group.

Statistical analysis

The categorical data were analyzed using the chi-squared test for independence or the Fisher’s exact test. The quantitative data were analyzed using the unpaired Student’s *t*-test for significance. If the data were not normally distributed as assessed by the Shapiro–Wilk test, the Wilcoxon rank-sum test was used. Descriptive parameters are represented as means

(SD). Highly skewed quantitative data are presented as median with interquartile range (IQR).

Repeated measures analysis of variance and student t-test were used to compare ~~Within and between-group comparisons of either~~ PTI, WLI, MAP, HR at six time points and PaO_2, lactic acid levels and blood glucose concentration at four time points~~ were performed using repeated measurement analysis of variance and Student's *t*-tests~~ within and between groups. Statistical significance was set at $P < 0.05$. All statistical analyses were performed using the SPSS 17.0.

Results

A total of 222 patients who from November 2018 to March 2019 were assessed for eligibility, finally 100 patients were enrolled in this clinical investigation and were comparable in the two groups (Fig 1). one patient turned to open surgery, one peripheral venous catheter slid out during surgery, one patient needed re-exploration for postoperative intra-thoracic bleeding on postoperative day 1, and the remaining ~~leaving~~ 97 patients were suitable ~~to be~~ for analysis~~zed~~ (Fig 1). The two groups had ~~were~~ similar characteristics at baseline (Tab 2).

Fig 1. Study protocol.

* Reasons for exclusion from the analysis: Group OFA: 1-converted to open lobetomy surgery. Group OA: 1-failure of PCA machine. 2-postopertive intra-thoracic bleeding requiring re-exploration on postoperative day 1.

Table 2. Clinical characteristics.

	Group OFA<br>n =49	Group OA<br>n =48	*P* –value

Gender (Female/Male)	27/22	29/19	0.68#
Age (IQR)	55 (47.5-60.5)	55 (50.3-59.3)	0.93*
Body Mass Index (BMI, IQR)	23.4 (21.3-25.5)	23.4 (22.2-26.4)	0.27*
Sites of VATS (Left/Right, n)	14/35	15/33	0.83#
Wedge resection/ Lobectomy/both: n	7/40/2	10/38/0	0.28#
Duration of anesthesia (SD, min)	151.3 (47.6)	160.8 (68.6)	0.44§
Duration of Surgery (SD, min)	137.1 (47.2)	144.6 (68.9)	0.54§
Fluid Infusion Volumes (IQR, ml)	1000 (1000-1250)	1100 (937.5-1263)	0.39*
Urinary volume （IQR, ml）	350 (250-500)	300 (225-500)	0.89*
Dexmedetomidine consumption (IQR, μg)	140 (130-165)	—	
Remifentanil consumption (IQR, mg)	—	1.3 (1.0-1.6)	
End-expiratory sevoflurane concentration at end of surgery (IQR, %)	1.4 (1.2-1.7)	1.3 (1.2-1.4)	0.15*
Ephedrine consumption (IQR, mg)	0 (0-6.0)	0 (0-5.3)	0.47*
Norepinephrine consumption (IQR, μg)	8 (0-46)	22 (0-85)	0.11*
Recovery time (SD, min)	13.2 (6.1)	9.5 (4.1)	< 0.001§
Extubation time (SD, min)	12.5 (5.7)	9.7 (4.1)	0.007§
Remove drainage tube time (IQR, day)	4 (3.5-6)	5 (4-6)	0.62*
Dizziness	2 (4.3%)	12 (25%)	0.005#
Postoperative nausea and vomiting	2 (4.3%)	20 (41.7%)	<0.001#
Exhaust time (IQR, day)	1 (1-1)	1 (1-2)	0.02*
Hospital Discharge time (95%CI, day)	7.2(4.2)	6.6(1.7)	0.66*

Data are presented as relative number of patients, mean ± standard deviation (SD), median (IQR). Times are shown in minutes.

* Wilcoxon rank-sum test; # Fisher's exact test; § Student's *t*-test.

Recovery time: time from intravenous injection of neostigmine and/or nalmefene to WLI >90; Extubation time: time from the completion of intravenous neostigmine and/or nalmefene to the removal of the tracheal tube; Exhaust time: the time from returning to the ward to the first exhaust. If the duration is less than 24h, it is recorded 1 day; 24h-48h is 2 days, 48h-72h is 3 days, and so on.

The results of s~~S~~ingle factor (time) repeated measurement analysis ~~results~~ showed that PTI values, WLI values, PaO_2, lactic acid levels and blood glucose concentrations of the two groups showed significant changes at different time points (*P* < 0.001). At the same time, the linear fit~~ting~~ test of the ~~change~~ trends of PTI, WLI, PaO_2, lactic acid and blood glucose under either opioid-free anesthesia or opioid-based anesthesia were also statistically significant (Tab 3 and 4, Fig 2 and 3, *P* <0.001).

Table 3. Summary of PTI and WLI with repeated measurement (mean, 95% CI)

	Group	PTI0	PTI1	PTI2	PTI3	PTI4	PTI5	*P*		
								Time	Group	Time*Group
PTI	OFA	91.8 (89.5-94.1)	88.2 (84.7-91.8)	61.9 (58.6-65.2) #	53.6 (50.0-57.3) #	59.7 (55.7-63.8) #	92.7 (90.8-94.6)	< 0.001*	0.86	0.62
	OA	91.3 (89.1-93.5)	88.8 (85.2-92.4)	63.0 (59.6-66.4) #	54.2 (50.1-57.8) #	60.6 (56.6-64.5) #	88.8 (84.7-92.9)			
WLI	OFA	96.3 (95.0-97.5)	82.7 (77.8-8.5) # ^	46.9 (42.8-51.1) # ^	47.8 (44.3-51.3) # ^	55.2 (51.4-59.0) # ^	88.4 (86.9-89.9) #	< 0.001*	< 0.001	< 0.001
	OA	95.4 (93.9-96.9)	90.9 (86.6-95.2)	54.5 (51.0-58.3) #	53.2 (50.6-55.8) #	59.5 (56.0-63.0) #	88.6 (87.2-89.9)			
MAP	OFA	106 (102-109)	107 (101-111)	115 (110-120) ^	88 (84 (92) #	89 (85-93) #	98 (94-102) ^	< 0.001*	0.89	<0.001

	OA	104 (101-108)	102 (99-106)	105 (101-110)	86 (83-89)$^{\#}$	93 (90-97)$^{\#}$	109 (105-113)			
HR	OFA	73 (69-76)	71 (68-74)	78 (74-81)	73 (70-76)	70 (66-73)	74 (71-76)$^{\wedge}$	< 0.001	0.004	0.001
	OA	76 (72-80)	75 (71-78)	82 (77-86)	74 (71-77)	74 (71-76)	87 (82-92)$^{\#}$			

PTI0: baseline value~~s~~, PTI1: 10min after infusion of loading does dexmedetomidine, PTI2: after intubation, PTI3: 30min after one-lung ventilation, PTI4: recovery of double lungs ventilation, PTI5: 5 min after extubation.

~~B~~WLI: ~~brain~~ wavelet index; PTI: pain threshold index; MAP: ~~M~~mean ~~A~~arterial ~~P~~pressure; HR: ~~H~~heart ~~R~~rate.

*: Linear fitting test (time) $P < 0.05$; $^{\#}$: Compared to PTI0 $P < 0.05$; $^{\wedge}$: Compared between two groups $P < 0.05$.

Table 4. Summary of PaO_2, Lactic and Blood glucose with repeated measurement (mean, 95% CI)

	Group	BGA0	BGA1	BGA2	BGA3	*P*		
						Time	Group	Time*Group
PaO_2	OFA	81.7 (78.8-84.5)	166.9 (137.4-196.3)$^{\#}$	282.6 (243.2-322.0)$^{\#\wedge}$	84.5 (78.3-90.7)	< 0.001*	0.39	0.01
	OA	81.6 (79.1-84.1)	190.6 (157.6-223.7)$^{\#}$	223.9 (188.6-259.2)$^{\#}$	82.5 (72.2-92.8)			
Lactic	OFA	1.2 (1.1-1.3)	1.2 (1.2-1.3)	1.2 (1.1-1.3)	1.4 (1.3-1.5)$^{\#}$	< 0.001*	0.71	0.06
	OA	1.3 (1.2-1.4)	1.2 (1.1-1.3)	1.2 (1.1-1.3)	1.5 (1.4-1.7)$^{\#}$			
Blood Glucose	OFA	5.6 (5.4-5.7)	6.7 (6.4-7.0)$^{\#\wedge}$	6.6 (6.4-6.8)$^{\#\wedge}$	6.3 (6.1-6.6)$^{\#}$	< 0.001*	0.004	< 0.001
	OA	5.8 (5.6-5.9)	5.8 (5.6-6.0)	5.9 (5.7-6.1)	6.2 (5.9-6.5)$^{\#}$			

BGA0: baseline value~~s~~, BGA1: 1h after surgery begun, BGA2: 2h after surgery begun, BGA3: 5 min after extubation.

PaO_2: arterial oxygen partial pressure.

*: Linear fitting test (time) $P < 0.05$; $^{\#}$: Compared to BGA0 $P < 0.05$; $^{\wedge}$: Compared between two groups $P < 0.05$.

Fig 2. Changes in ~~brain~~ wavelet index, pain threshold index, mean arterial pressure and heart rate (mean ±SD) between two groups.

~~B~~WLI: ~~brain~~ wavelet index; PTI: pain threshold index; MAP: mean arterial pressure; HR: heart rate. PTI0: baseline value~~s~~, PTI1: 10min after dexmedetomidine infusion (1μg kg^{-1}), PTI2: after intubation, PTI3: 30min after one lung ventilation, PTI4: both lungs ventilation, PTI5: 5 min after extubation. *: $p < 0.05$ between groups

Fig 3: Changes in arterial oxygen partial pressure, lacti~~cate~~ ~~concentration~~ and blood

glucose with blood gas (mean±SD) between two groups.

BGA0: baseline values, BGA1: 1h after surgery begun, BGA2: 2h after surgery begun, BGA3: 5 min after extubation. PaO_2: arterial oxygen partial pressure. *: $p < 0.05$ between two groups.

PTI readings between two groups were not significantly different at six time-points during the surgery ($P > 0.05$, Fig 2). The WLI readings at PTI1, PTI2, PTI3 and PTI4 were statistically significant lower in group OFA than those in group OA (such as 46.7 at PTI2 in group OFA vs. 54.5 at PTI2 in group OA; difference, 9.5 [95% CI, 3 to 13]; $P < 0.001$), and there was also the interaction between grouping factors * time factors ($P < 0.001$).

The results of s~~S~~ingle factor (time) repeated measurement analysis ~~results~~ showed (Table 3, Fig 3) that ~~(Table 3, Fig 3)~~ the blood glucose levels of patients ~~under~~ without opioid~~-free~~ anesthesia was significantly increased (20%) at BGA1, BGA2 and BGA3 ($P < 0.01$) which can be considered ~~that~~ as ~~the~~ blood glucose ~~value~~ levels increased ~~in the whole period of~~ during the operation. There was~~ere~~ no~~t~~ significant difference ~~changes of~~ in blood glucose levels ~~in patients under group OA~~ at different time points in the group OA ($P > 0.05$). The results ~~from~~ of multivariate ~~multiple factors~~ repeated measures~~ment~~ analysis of variance showed that the difference in blood glucose ~~value~~ levels between the two anesthesia methods was statistically significant ~~between the two anesthesia methods~~ ($P < 0.001$).

The ~~mean~~ recovery time and extubation time of the OFA group were significantly prolonged~~r in group OFA~~ ($P < 0.05$). There were no~~t~~ significantly differen~~t~~ce in PaO_2, pH and SpO_2 between the two groups during ~~the~~ operation. There was n~~N~~o local infections or hematomata ~~were detected~~ at the puncture site 24 h after operation. ~~No patient died, was transferred to ICU and respiratory dysfunction, and no patient was intubated again after the~~

~~tracheal intubation was removed.~~ During the follow-up period, ~~N~~no patient was transferred to the ICU, had respiratory insufficiency, need reintubation, or died ~~during the follow-up~~. ~~In group OA, one patient received~~ ~~a~~Acute cerebral infarction occurred in 1 case in group OA, and ~~one patient met~~ atrial fibrillation occurred in 1 case after ~~surgery~~ operation. No patients in group OFA ~~developed~~ had hypoxia (SpO_2 < 90%), hypotension or bradycardia requiring treatment.

Discussion

This prospective randomized study showed that compared with opioid anesthesia under the guidance of PTI monitoring in patients receiving VATS, our ~~opioid-free anesthesia~~ OFA ~~regimen~~ program c~~ould~~an provide the same sufficient ~~equally adequate~~ intraoperatively ~~analgesia-nociception balance~~ ~~in~~ for ASA Ⅰ-Ⅱ, 18-65- years- old, non-obese patients Aanalgesia-nociception balance ~~(ASA Ⅰ-Ⅱ, 18-65 years old, non-obese)~~ ~~compared to opioid based anesthesia guided by PTI monitoring in patients undergoing VATS.~~, ~~h~~However, ~~with OA in~~ the group OFA~~,~~ had~~the intraoperative depth of sedation was~~ deeper intraoperative sedation~~,~~ and higher ~~the~~ blood glucose levels ~~were higher~~, ~~than those levels in patients with OA~~.

In the present study, a non-opioid anesthesia regimen was effective in controlling the ~~intraoperative~~ balance of nociception–antinociception ~~in~~ of patients undergoing VATS, with intraoperative pain management guided by the PTI rather than via hemodynamic indices. The ~~intraoperative~~ PTI readings in both groups were similar at all the time points ~~during surgery~~ and effectively maintained the optimal range from 40 to 60 during surgery, which is thought to predict adequate analgesia to nociceptive stimuli. Compared with OA, for patients who

are unconscious under general anesthesia, ~~T~~there are few reports on the efficacy of ~~concerning the~~ intraoperative monitoring of the ~~potency of~~ nociception-anti-nociception ~~in~~ of OFA ~~compared to OA for unconscious patients under general anesthesia~~; therefore, the effect of non-opioid anesthesia ~~is~~ has been criticized [21]. PTI has ~~arisen as~~ become a new parameter ~~for the~~ to balance ~~of~~ nociception and analgesia ~~in~~ during surgery, ~~as well as for an~~ emphasizes ~~on~~ the adequacy of perioperative analgesia in unconscious patients [22-24]. OFA is a multimodal anesthesia ~~that~~ associated~~s~~ with hypnotics, N-methyl-D-aspartate antagonists, local anesthetics, anti-inflammatory drugs, and α-2 agonists. Tripathy and colleagues [25] found that patients undergoing magnetic resonance mammography with axillary dissection for breast cancer achieved superior analgesia with opioid-free pectoral nerve block-based anesthesia that involved dexmedetomidine, propofol, and isoflurane. Due to the fact that there is specific type of OFA protocol, dexmedetomidine, sevoflurane and TPVB were used in our study to replace opioids ~~anesthesia~~[26]. Dexmedetomidine is a highly, potent, selective α-adrenergic agonist with intrinsic analgesic properties that can be prolonged for at least 12 h after surgery~~,~~. ~~and~~ Due to its analgesic properties, it has been used as an opioid substitute in various surgical interventions~~, due to its analgesic properties~~ [10, 27-29]. ~~Based on~~ According to a meta-analysis review, Grape and colleagues demonstrated that intraoperative dexmedetomidine was superior to remifentanil and its ~~with improved~~ pain improvement can ~~outcomes that~~ last up to 24 ~~postoperative~~ hours after surgery [30]. A loading dose of dexmedetomidine (1 μg kg^{-1}) infusion before the induction of anesthesia usually exhibits a central anti-sympathetic effect that may deepen anesthesia and spare the dose of anesthetics, and it can provide an optimal balance between adequate postoperative analgesic properties and adverse effects in sensory nerve blocks [31]. Han and colleagues reported that the

intraoperative administration of a loading dose of dexmedetomidine (1 μg kg^{-1}) combined with sevoflurane (compared to the use of propofol) led to a significant stability in blood pressure during the operative period [32]. When compared with systemic agents, regional blockade is most effective for controlling movement-evoked pain [33]. PVB ~~will~~ not only cause dense somatic afferent blockades, but (due to the anatomy of the paravertebral space) ~~will~~ it also completely blocks transmission within the sympathetic chain. TPVB can produce ~~equally potent~~ the same effective analgesia (compared to the thoracic-epidural-blocks) and can reduce the consumption of opioids without the negative events associated with epidural blocks [34]. In a prospective, randomized, blinded study ~~in~~ patients ~~undergoing~~ receiving VATS, the authors observed relief of pain (especially pain during coughing) after ~~that~~ TPVB ~~postoperatively improved pain relief (especially for pain on coughing)~~ for up to 48 hours. In ~~A~~additionally, intravenous infusion of dexmedetomidine ~~infusion~~ significantly increased the duration of intermuscular~~lene~~ sulcus brachial plexus block analgesia and reduced cumulative opioid consumption [35, 36].

The WLI is a similar EEG-derived parameter to the BIS and is used to monitor the depth of sedation under GA [22, 37, 38]. Our results showed that with similar PTI readings between the two groups, WLI readings in the OFA group showed a more apparent decrease (10%) than those readings in the OA group. In addition, dexmedetomidine produced a state resembling stage 2 NREM sleep in the EEG with delta and alpha ranges of activity and characteristic arousal sedation [39]. Xu and colleagues [37] reported that, after dexmedetomidine infusion (a loading dose of 0.75 μg kg^{-1} and a continuous infusion of 0.5 μg kg^{-1} h^{-1}), the calculated cutoff WLI readings ranged from 55.8-93.7. Yan and Feng reported that injection equivalent analgesia doses of sufentanil (0.2 μg kg^{-1}) and remifentanil

(2 μg kg^{-1}) produced mildly decreased WLI readings from 84.7-98.6 and 91.4-99.0 respectively [40]. Zhang and colleagues reported that, in patients undergoing posterior lumbar interbody fusion surgery with inhalation sevoflurane ranging from 1.5 vol % to 4 vol %, the WLI readings at the incision were 46.7 ~~at incision~~ and 60 at skin closure [41]. Gu and colleagues reported that due to less propofol consumption, as well as different action sites between dexmedetomidine and propofol, the BIS value ~~of~~ for los~~sing~~ of consciousness was higher in a ~~1μg kg^{-1}~~ loading dose of dexmedetomidine (1μg kg^{-1}) with propofol than with an infusion ~~of solely~~ propofol alone ~~due to less propofol consumption, as well as different action sites between dexmedetomidine and propofol~~ [37]. In our study, the consumption of sevoflurane during surgery was not recorded; therefore, we were not sure ~~as to~~ whether the lower WLI in ~~the~~ OFA is caused by the effects ~~resulted from the action~~ of sevoflurane ~~which~~ that requires further research. Our results showed that deeper sedation in the non-opioid anesthesia regimen with dexmedetomidine and sevoflurane (compared to opioid-based anesthesia with remifentanil and sevoflurane) was statistically significant during the in period of surgery, and the WLI readings in both groups were maintained at normal values ranging from 40-60.

Our results ~~showed~~ suggested that, in patients under non-opioid anesthesia, the intraoperative blood glucose values increased significantly until the end of surgery. The increase in plasma glucose concentration is one of the main features of the metabolic response to surgery and simultaneously demonstrates the effect of different anesthesia techniques on the stress response [42]. Dexmedetomidine attenuates the intraoperative surgical stress response by decreasing nociceptive transmission and ~~by~~ decreasing cortisol levels, thus leading to ~~reduced~~ lower blood glucose levels [43, 44]. In contrast, a recent paper

showed that α2A-adrenoreceptors, which are present on pancreatic β-cells, is an important regulator of blood glucose homeostasis, and the activation of these receptors inhibits insulin secretion [45]. Therefore, dexmedetomidine may cause hypoinsulinaemia and a resultant state of hyperglycemia. Ghimire and colleagues [46] reported that 0.4 μg kg^{-1} dexmedetomidine decreased plasma insulin levels and mildly increased plasma glucose concentrations (4%) in healthy fasting human subjects. Görges and colleagues reported that in children undergoing elective surgery with total intravenous anesthesia, elevations in blood glucose levels (20%) were observed 15 minutes after the induction of anesthesia[47]. Additionally, sevoflurane anesthesia has been shown to increase plasma glucose levels by impairing insulin secretion, inducing insulin resistance, and reducing glucose uptake and utilization [48, 49]. The effects of sevoflurane combined with dexmedetomidine on blood glucose levels and insulin sensitivity in general anesthesia have not been reported yet. In our study, the blood glucose value was determined with the use of arterial blood gases to avoid tissue uptake and use glucose. ~~In our study,~~ Our results showed that the lactic acid levels ~~showed that there was~~ were no significant difference between the two groups at the time points. The reasons for the increase in blood glucose concentrations in patients under nonopioid anesthesia are that insulin secretion is inhibited by dexmedetomidine and sevoflurane and/or nonopioid anesthesia could not effectively control the stress response. ~~with t~~These possible mechanisms requiring future studies.

This study had several limitations. First of all, intraoperative anesthesiologists c~~ould~~annot ~~be~~ turn a blind~~ed~~ eye to ~~the~~ interventions. Second, in our study, single-lumen bronchial intubation and BB (instead of a dual-lumen endotracheal tube) were used to ~~lower~~ reduce the stress response to intubation and extubation~~, (instead of a dual-lumen~~

~~endotracheal tube), were used for one-lung ventilation in our study~~. Third, it ~~wa~~is impossible to accurately calculate the amount ~~consumption~~ of sevoflurane consumed during the operation; th~~us~~erefore, the end-~~expiratory~~tidal sevoflurane concentrations of the two groups at the end of ~~surgery in both groups~~ the operation w~~ere~~as compared. ~~When c~~Considering that sevoflurane may affect the intraoperative sedation index and analgesia index, we began to calculate the consumption of sevoflurane at different times during the operation in follow-up experiments. Fourth, our research is a study ~~was~~ of a single~~-~~institution~~, study,~~ with a small sample size, and some more detailed modifications have been made to some programs. There are no data reports on PI, ~~were reported~~ ~~that changing in primary and secondary outcomes (there are not data on PI because~~ ~~since~~ because PI can ~~be~~ only be used in conscious patients~~;~~ and the serum potassium concentration ~~wa~~is not recorded, because some patients are supplemented with ~~were given~~ potassium ~~supplementation~~ ~~because the blood gas in the room showed low potassium)~~, which ~~potentially results in a~~ may lead to selection bias. Fifth, we cannot perform an intention-to-treat analysis including the 3 patients excluded after randomization since we did not follow up these patients. ~~the ITT analysis included all randomized patients to fully observe the effect of the intervention (exhaust time, PONV and length of stay). Unfortunately, we did not follow up the excluded patients.~~ Further studies will be needed to assess the long-term benefits of the avoidance of opioid use in lung cancer patients, as well as to assess the benefits of increasing survival.

Conclusions

OFA with dexmedetomidine and sevoflurane plus TPVB ~~appear~~seems to be feasible for the management of intraoperative nociception ~~in VATS for lung cancer patients~~ in ASA I-

II, 18-65 years old, non-obese patients undergoing VATS for lung cancer.~~;~~ ~~h~~However, a deeper sedation level was produced in OFA than in OA for the achievement of an optimal balance of analgesia-nociception. We also identified significant elevation in blood glucose levels after the beginning of operation with opioid-free anesthesia. The patients under opioid anesthesia had ~~mild~~ slight changes in intraoperative blood levels. Further studies are required to evaluate the sedation depth of OFA with WLI as the primary outcome and to observe blood glucose levels in diabetic patients who accept OFA.

Acknowledgements

The authors would like to thank the operating room teams of the Fourth Hospital of Hebei University, including nurses and surgeons, for their help in permitting the experimental protocol to take place.

Reference

1. Chen Z, Ali JM, Xu H, Jiang L, Aresu G. Anesthesia and enhanced recovery in subxiphoid video-assisted thoracoscopic surgery. J Thorac Dis. 2018;10~~(12)~~:6987-92. ~~doi: 10.21037/jtd.2018.11.90. PubMed PMID: 30746244; PubMed Central PMCID: PMCPMC6344778.~~
2. Fletcher D, Martinez V. How can we prevent opioid induced hyperalgesia in surgical patients? Br J Anaesth. 2016;116~~(4)~~:447-9. ~~Epub 2016/03/20. doi: 10.1093/bja/aew050. PubMed PMID: 26994226.~~
3. Practice Guidelines for the Prevention, Detection, and Management of Respiratory Depression Associated with Neuraxial Opioid Administration: An Updated Report by the American Society of Anesthesiologists Task Force on Neuraxial Opioids and the American Society of Regional Anesthesia and Pain Medicine. Anesthesiology 2016;124~~(3)~~:535-52. ~~doi: 10.1097/ALN.0000000000000975>.~~
4. Smith HS, Laufer A. Opioid induced nausea and vomiting. Eur J Pharmacol. 2014;722:67-78. ~~Epub 2013/10/26. doi: 10.1016/j.ejphar.2013.09.074. PubMed PMID: 24157979.~~
5. Harold S Minkowitz, Richard Scranton, Stephen K Gruschkus, Kathy Nipper-Johnson, Laura Menditto, Akash Dandappanavar. Development and Validation of a Risk Score to Identify Patients at High Risk for Opioid-Related Adverse Drug Events. J Manag Care Spec Pharm. 2014;20~~(9)~~:948-58.
6. Cata JP, Keerty V, Keerty D, Feng L, Norman PH, Gottumukkala V, et al. A retrospective analysis of the effect of intraoperative opioid dose on cancer recurrence after non-small cell lung

cancer resection. Cancer Med. 2014;3(4):900-8. doi: 10.1002/cam4.236. PubMed PMID: 24692226; PubMed Central PMCID: PMCPMC4303157.

7. Singleton PA, Mirzapoiazova T, Hasina R, Salgia R, Moss J. Increased mu-opioid receptor expression in metastatic lung cancer. British journal of anaesthesia. 2014;113 Suppl 1:i103-8. doi: 10.1093/bja/aeu165. PubMed PMID: 24920011; PubMed Central PMCID: PMCPMC4111280.

8. Maher DP, Wong W, White PF, McKenna R, Jr., Rosner H, Shamloo B, et al. Association of increased postoperative opioid administration with non-small-cell lung cancer recurrence: a retrospective analysis. British journal of anaesthesia. 2014;113 Suppl 1:i88-94. doi: 10.1093/bja/aeu192. PubMed PMID: 25009195.

9. Oh TK, Jeon JH, Lee JM, Kim MS, Kim JH, Cho H, et al. Investigation of opioid use and long-term oncologic outcomes for non-small cell lung cancer patients treated with surgery. PLoS One. 2017;12(7):e0181672. doi: 10.1371/journal.pone.0181672. PubMed PMID: 28732026; PubMed Central PMCID: PMCPMC5521798.

10. Bakan M, Umutoglu T, Topuz U, Uysal H, Bayram M, Kadioglu H, et al. Opioid-free total intravenous anesthesia with propofol, dexmedetomidine and lidocaine infusions for laparoscopic cholecystectomy: a prospective, randomized, double-blinded study. Braz J Anesthesiol. 2015;65(3):191-9. Epub 2015/05/01. doi: 10.1016/j.bjane.2014.05.001. PubMed PMID: 25925031.

11. Swagata Tripathy SR, Suresh Agrawal, P Bhaskar Rao, A Panda, T S Mishra, and Sukdev Nayak. Opioid-free anesthesia for breast cancer surgery: An observational study. J Anaesthesiol Clin Pharmacol. 2018;34(1):35-40. doi: 10.4103/joacp.JOACP_143_17.

12. Ziemann-Gimmel P, Goldfarb AA, Koppman J, Marema RT. Opioid-free total intravenous anaesthesia reduces postoperative nausea and vomiting in bariatric surgery beyond triple prophylaxis. Br J Anaesth. 2014;112(5):906-11. Epub 2014/02/21. doi: 10.1093/bja/aet551. PubMed PMID: 24554545.

13. Kim DJ, Bengali R, Anderson TA. Opioid-free anesthesia using continuous dexmedetomidine and lidocaine infusions in spine surgery. Korean J Anesthesiol. 2017;70(6):652-3. Epub 2017/12/12. doi: 10.4097/kjae.2017.70.6.652. PubMed PMID: 29225750; PubMed Central PMCID: PMCPMC5716825.

14. Bello M, Oger S. Bedon-Carte S, Vielstadte C, Leo F, Zaouter C, et al. Effect of opioid-free anaesthesia on postoperative epidural ropivacaine requirement after thoracic surgery: A retrospective unmatched case-control study. Anaesth Crit Care Pain Med. 2019;38(5):499-505. Epub 2019/02/08. doi: 10.1016/j.accpm.2019.01.013. PubMed PMID: 30731138.

15. Guinot PG, Spitz A, Berthoud V, Ellouze O, Missaoui A, Constandache T, et al. Effect of opioid-free anaesthesia on post-operative period in cardiac surgery: a retrospective matched case-control study. BMC Anesthesiol. 2019;19(1):136. Epub 2019/08/02. doi: 10.1186/s12871-019-0802-y. PubMed PMID: 31366330; PubMed Central PMCID: PMCPMC6668113.

16. Lavand'homme P, Estebe JP. Opioid-free anesthesia: a different regard to anesthesia practice. Curr Opin Anaesthesiol. 2018;31(5):556-61. Epub 2018/07/12. doi: 10.1097/ACO.0000000000000632. PubMed PMID: 29994942.

17. An JX, Wang Y, Cope DK, Williams JP. Quantitative Evaluation of Pain with Pain Index Extracted from Electroencephalogram. Chin Med J (Engl). 2017;130(16):1926-31. Epub 2017/08/05. doi: 10.4103/0366-6999.211878. PubMed PMID: 28776544; PubMed Central PMCID: PMCPMC5555126.

18. Hao Kong, Hong Zhang. Research progress of noxious pricking stimulation and analgesic water level monitoring during operation. Journal of Clinical Anesthesiology. 2020;36(06):612-5.

19. Chen Yingqi, Yue Yun, Qing Enming, Lin Duomao, Yang Baxian, Feng Yi, et al. Nalmefene antagonizes the effect of opioid postoperative respiratory depression-a multicenter, randomized, double-blind, positive drug control study. Chinese Journal of Anesthesiology,. 2011;31(3):307-9.

20. Jia Zhen, Chen Yi, Zhang Linlin, Wang Xin, Zhao Yuan, Guo Suqian, et al. The effect of different low-dose nalmefene in preventing postoperative hyperalgesia induced by remifentanil. Chinese Journal of Anesthesiology,. 2017;37~~(10)~~:1159-62.

21. Frauenknecht J, Kirkham KR, Jacot-Guillarmod A, Albrecht E. Analgesic impact of intra-operative opioids vs. opioid-free anaesthesia: a systematic review and meta-analysis. Anaesthesia. 2019;74~~(5)~~:651-62. ~~doi: 10.1111/anae.14582.~~

22. Wu L, Wang S, Wang Y, Zhang K, Bai J, Zheng J. Prediction of Hemodynamic Reactivity by Electroencephalographically Derived Pain Threshold Index in Children Undergoing General Anesthesia: A Prospective Observational Study. J Pain Res. 2019;12:3245-55. ~~Epub 2019/12/11. doi: 10.2147/JPR.S231596. PubMed PMID: 31819608; PubMed Central PMCID: PMCPMC6899069.~~

23. Zhen Su, Lijun An, Yang Zhang, ~~Lian~~Penghua Chen. The clinical value of analgesia index in evaluating the degree of analgesia during general anesthesia. Chinese Journal of Applied Physiology. 2018;34~~(5)~~:461-3.

24. ~~J i a n x i n . ZQYWJJZMY. Application of target-controlled infusion of remifentanil guided by pain threshold index in laparo-scopic subtotal gastrectomy. J Clin Anesthesiol. 2020;36(7):634-7.~~ Zhu Qingqing, Yue Wei, Jiang Jingnan, Zhang Miao, Yang Jianxin. Application of target-controlled infusion of remifentanil guided by pain threshold index in laparo-scopic subtotal gastrectomy. J Clin Anesthesiol. 2020;36:634-7

25. Tripathy S, Rath S, Agrawal S, Rao PB, Panda A, Mishra TS, et al. Opioid-free anesthesia for breast cancer surgery: An observational study. J Anaesthesiol Clin Pharmacol. 2018;34~~(1)~~:35-40. ~~Epub 2018/04/13. doi: 10.4103/joacp.JOACP_143_17. PubMed PMID: 29643620; PubMed Central PMCID: PMCPMC5885445.~~

26. Dario Bugada, L~~uca~~ FL, Patricia Lavand'homme. Opioid free anesthesia: evidence for short and long-term outcome. Minerva Anestesiol. 2021;87~~(2)~~:230-7.

27. Kim DJ, Bengali R, Anderson TA. Opioid-free anesthesia using continuous dexmedetomidine and lidocaine infusions in spine surgery. Korean J Anesthesiol. 2017;70~~(6)~~:652-3. ~~doi: 10.4097/kjae.2017.70.6.652. PubMed PMID: 29225750; PubMed Central PMCID: PMCPMC5716825.~~

28. Kumar K, Kirksey MA, Duong S, Wu CL. A Review of Opioid-Sparing Modalities in Perioperative Pain Management: Methods to Decrease Opioid Use Postoperatively. Anesth Analg. 2017;125~~(5)~~:1749-60. ~~doi: 10.1213/ANE.0000000000002497. PubMed PMID: 29049119.~~

29. Gaszynski T, Gaszynska E, Szewczyk T. Dexmedetomidine for awake intubation and an opioid-free general anesthesia in a superobese patient with suspected difficult intubation. Drug Des Devel Ther. 2014;8:909-12. ~~doi: 10.2147/DDDT.S64587. PubMed PMID: 25045249; PubMed Central PMCID: PMCPMC4094577.~~

30. Grape S, Kirkham KR, Frauenknecht J, Albrecht E. Intra-operative analgesia with remifentanil vs. dexmedetomidine: a systematic review and meta-analysis with trial sequential analysis. Anaesthesia. 2019;74~~(6)~~:793-800. ~~Epub 2019/04/06. doi: 10.1111/anae.14657. PubMed PMID: 30950522.~~

31. Motaghi E, Ghasemi Pirbaooti M, Bozorgi, H. Eslami, M. Rashidi, M. Safety and Efficacy of Dexmedetomidine in Breast Surgeries: A Systematic Review and Meta-Analysis. J Perianesth Nurs. ~~2020. Epub 2020/12/12. doi: 10.1016/j.jopan.2020.09.011. PubMed PMID: 33303343.~~ 2021;36:179-186.

32. Han Y, Han L, Dong M, Sun Q, Ding K, Zhang Z, et al. Comparison of a loading dose of dexmedetomidine combined with propofol or sevoflurane for hemodynamic changes during anesthesia maintenance: a prospective, randomized, double-blind, controlled clinical trial. BMC Anesthesiol. 2018;18~~(1)~~:12. ~~Epub 2018/01/26. doi: 10.1186/s12871-018-0468-x. PubMed PMID: 29368594; PubMed Central PMCID: PMCPMC5784722.~~

33. Shanthanna H, Ladha KS, Kehlet H, Joshi GP. Perioperative Opioid Administration. Anesthesiology. 2021;134~~(4)~~:645-59. E~~pub 2020/09/30. doi: 10.1097/ALN.0000000000003572. PubMed PMID: 32991672.~~

34. Agnoletti V GC, Piraccini E, Maitan S, Corso MR, Buccioli M, Poletti V, et al. Efficacy and safety of thoracic paravertebral block for medical thoracoscopy. Br J Anaesth. 2011;106~~(6)~~:916-7.

35. RyungA Kang, Ji Seon Jeong, Jae Chul Yoo, Ju Hyun Lee, Soo Joo Choi, Mi Sook Gwak, et al. Effective Dose of Intravenous Dexmedetomidine to Prolong the Analgesic Duration of Interscalene Brachial Plexus Block: A Single-Center, Prospective, Double-Blind, Randomized Controlled Trial. Reg Anesth Pain Med. 2018;43~~(5)~~:488-95.

36. Abdallah FW,Dwyer T, Niazi AU, Ogilvie-Harris DJ, Oldfield S, et al. IV and Perineural Dexmedetomidine Similarly Prolong the Duration of Analgesia after Interscalene Brachial Plexus Block: A Randomized, Three-arm, Triple-masked, Placebo-controlled Trial. Anesthesiology. 2016;124~~(3)~~:683-95. ~~Epub 2015/12/10. doi: 10.1097/ALN.0000000000000983. PubMed PMID: 26649424.~~

37. ~~XU Li LZ, TIAN Guogang. Evaluation of wavelet index in monitoring sedation depth with dexmedetomidine under combined spinal epidural anesthesia. J Clin Anesthesiol. 2018;34(12):1202-4.~~ Xu Li, LI Zhao, Tian Guogang. Evalution of wavelet index in monitoring sedation depth with dexmedetomidine under combined spinal epidural anesthesia. J Clin Anesthesiol. 2018;34:1202-4.

38. Chun Xia, Yue Yun. Comparative investigation of wavelet index and bispectral index in monitoring depth of anesthesia. J Clin Anesthesiol. 2010;26~~(10)~~:836-9.

39. Yang Gu, Fan Yang, Yonghai Zhang, Junwei Zheng, Jie Wang, Bin Li, et al. The effects of different doses of dexmedetomidine on the requirements for propofol for loss of consciousness in patients monitored via the bispectral index a double-blind, placebo-controlled trial BMC Anesthesiol. 2020;20~~(1)~~:96-102. ~~doi: 10.1186/s12871-020-01013-x.~~

40. Qi Yan, Yi Feng. Differences in sedation level and adverse effects produced by equivalent analgesic doses of remifentanil, sufentanil and fentanyl. Chin J Anesthesiol. 2012;32(7):853-6.

41. Zhang JW, Lv ZG, Kong Y, Han CF, Wang BG. Wavelet and pain rating index for inhalation anesthesia: A randomized controlled trial. . World J Clin Cases;. 2020;8~~(21)~~:5221-34.

42. Desborough JP. The stress response to trauma and surgery. Br J Anaesth. 2000;85~~(1)~~:109-17. Epub 2000/08/06. doi: 10.1093/bja/85.1.109. PubMed PMID: 10927999.

43. Li CS, ~~Li~~ Liu SF~~L~~, ~~Y~~ Zhou Y, Lu X H ~~Lu.~~ Effect of Dexmedetomidine on Perioperative Stress and Postoperative Pain in Patients With Radical Resection of Esophageal Cancer Under Combined Thoracoscope and Laparoscope. Zhonghua Yi Xue Za Zhi 2018;98:3778-83.

44. Swarnendu Dey, Meenakshi Kumar. Comparison of pretreatment with dexmedetomidine with midazolam for prevention of etomidate-induced myoclonus and attenuation of stress response at intubation: A randomized controlled study. J Anaesthesiol Clin Pharmacol 2018;34:94-8.

45. Fagerholm V, Haaparanta M, Scheinin M. alpha2-adrenoceptor regulation of blood glucose homeostasis. Basic Clin Pharmacol Toxicol. 2011;108(6):365-70. Epub 2011/03/23. doi: 10.1111/j.1742-7843.2011.00699.x. PubMed PMID: 21418144.

46. Ghimire LV, Muszkat M, Sofowora GG, Scheinin M, Wood AJ, Stein CM, et al. Variation in the alpha(2A) adrenoceptor gene and the effect of dexmedetomidine on plasma insulin and glucose. Pharmacogenet Genomics. 2013;23(9):479-86. Epub 2013/07/23. doi: 10.1097/FPC.0b013e3283642f93. PubMed PMID: 23873118; PubMed Central PMCID: PMCPMC3787837.

47. Görges M, Poznikoff AK, West NC, Brodie SM, Brant RF, Whyte SD. Effects of Dexmedetomidine on Blood Glucose and Serum Potassium Levels in Children Undergoing General Anesthesia: A Secondary Analysis of Safety Endpoints During a Randomized Controlled Trial. Anesthesia & Analgesia. 2019;129(4):1093-9. doi: 10.1213/ane.0000000000004154.

48. O. Y. COK, Z Ozkose, H. PasaogluASAOGLU, S. YardimARDIM. -Glucose response during craniotomy propofol-remifentanil versus isoflurane-remifentanil. Minerva Anestesiol 2011;77:1141-8.

49. Katsuya Tanaka TK, Takehito Tomino, Hiroaki Kawano, Tsuyoshi Okada, Shuzo Oshita, Akira Takahashi, Yutaka Nakaya, et al. Mechanisms of Impaired Glucose Tolerance and Insulin Secretion during Isoflurane Anesthesia. Anesthesiology. 2009;111:1044-51.

Supporting information

S1 File. CONSORT checklist

S2 File. Ethic Document Comments

S3 File. Ethic Document Comments-English

S4 File. Informed Consent- Original

S5 File. Informed Consent-English

S6 File. Study Protocol-English

S7 File. Study Protocol-Original

S8 File. Language Editing Certificate

Dear Dr Alessandro Putzu:

On behalf of my co-authors, we thank you very much for giving us an opportunity to revise our manuscript, we appreciate editor and reviewers very much for their precious comments and advice.

Comments and suggestions on our manuscript entitled ‘Lower sedation index and raised blood glucose with opioid-free anesthesia compared to opioid anesthesia for lung cancer patients undergoing video-assisted thoracoscopic surgery: a prospective, randomized, controlled study’(PONE-D-20-27554) are all valuable and immensely helpful for revising and improving our paper, as well as the important guiding significance to our research. We have studied comments carefully and have made correction which we hope meet with approval. Revised portions are marked in yellow in the paper.

We would like to express our great appreciation to you and reviewers for comments on our paper. We would be glad to respond to any further questions and comments that you may have. Looking forward to hearing from you.

Thank you and best wishes.

Yours sincerely,

Xuelian Zhao

9/6/2021

Here is our response to academic editors.

Major comments:

1. Comments. You should insert authors contributions (see PLOS One guidelines)

Author response: Thank you for your suggestion. As suggested by reviewer, we have inserted author contributions in title page and submitted online.

2. Comments. I suggest to use a structured abstract (not exceeding 300 words)

Author response: We are grateful for the suggestion. To be more clearly and in accordance with the comment, we have converted the abstract into a structured abstract, see abstract with yellow labels.

3. Comments. Abstract. You should cite the EEG monitoring system in the abstract.

Author response: We agree with your comment. We have added the EEG monitoring system in the abstract, see Lines 14-15 with yellow labels.

4. Comments. Abstract. The TPVB abbreviation should be removed from the abstract.

Author response: Thank you for pointing this out. We have removed the TPVB abbreviation from the abstract, see Lines 11 with yellow labels.

5. Comments. Abstract, line 20-24. It should read: “This study showed that our OFA

regimen achieved equally effective intraoperative pain threshold index compared to OA in video-assisted thoracoscopic surgery. Depth of sedation was significantly lower and blood glucose levels were higher with OFA.”

Author response: We do think these are excellent suggestions which makes the manuscript more rigorous and concise (Lines: 27-31, marked with yellow).

6. Comments. Introduction, line 39. It should read: “…respiratory depression [3], postoperative nausea and vomiting (PONV) [4] as well as postoperative delirium…”

Author response: Thank you for pointing this out. We have added ‘postoperative’ in lines 47 and marked with yellow.

7. Comments. Methods, trial design, line 85-88. This paragraph should be removed: “Because midazolam is associated with postoperative delirium, midazolam is no longer used before anesthesia in patients undergoing thoracic surgery in our hospital. The eligible patient was informed that they are not received midazolam and signed a written informed consent form after obtaining the consent of the participant.”

Author response: We deeply appreciate the reviewer’s suggestion. We have deleted the paragraph (Lines:95-98, yellow marked), and added the sentence “The eligible patient signed a written informed consent form after obtaining the consent of the participant.” (Lines:106-107, yellow marked).

8. Comments. Methods, allocation and blinding, line 97. You should not report

results in the methods. It should read: “Patients were randomized in two groups (group OA and group OFA) using computer-generated random numbers (Excel® version 16)”

Author response: We do think this is good suggestion. We have corrected it, see line 110.

9. Comments. Methods, Anesthetic management protocol, page 8, line 140-141. It should read: “Sevoflurane was adjusted to maintain anesthesia. The use of midazolam was avoided. The patients were maintained on mechanical ventilation to keep the ….”

Author response: We do think this is excellent suggestion. We have made changes in relevant places in the manuscript marked with yellow, line 156-157.

10. Comments. Methods, outcomes, page 10, line 183. Exhaust time should be defined

Author response: Thank you for pointing this out. We have added the definition of exhaust time (Lines: 191, marked in yellow).

11. Comments. Methods, outcomes, page 10, line 185-190. The English of the paragraph should be improved.

Author response: We apologize for the language problems in the manuscript. Language presentation was improved with assistance from a native

English speaker with appropriate research background (Lines: 193-203, marked with yellow).

12. Comments. Results, page 14, line 283-284. This sentence should be improved "No patient died, was transferred to ICU and respiratory dysfunction, and no patient was intubated again after the tracheal intubation was removed". If judged correct, it should read: "No patient was transferred to the ICU, had respiratory insufficiency, need reintubation, or died during the follow-up".

Author response: Our deepest gratitude goes to you for your careful work and thoughtful suggestions that have helped improve this paper substantially (Lines: 303-307, marked with yellow).

13. Comments. Discussion, first paragraph, line 290-295. It should read: "This prospective randomized study showed that our OFA regimen could provide equally adequate intraoperative analgesia-nociception balance in non-obese, 18-65 years old, ASA I-II patients compared to opioid based anesthesia guided by PTI monitoring in patients undergoing VATS. However, in group OFA, the intraoperative depth of sedation was deeper, and the blood glucose levels were higher, than those levels in patients with OA."

Author response: Thank you for your precious comments and advice. The comment is all valuable and extremely helpful for revising and improving our paper. We have revised the manuscript accordingly, see line 313-318and yellow-marked.

14. Comments. Discussion, line 312-313. Ij judged correct, it should read: "…sevoflurane and TPVB were used in our study to replace opioids[26]."

Author response: Thank you for pointing this out. We have re-written this sentence (Lines: 340, yellow-marked).

15. Comments. Discussion, page 16, line 332. It should read: "…postoperatively improved pain relief (especially pain on coughing) up to 48 h…."

Author response: Thank you for pointing this out. We have rewritten this sentence (Lines: 359-361, marked with yellow).

16. Comments. Discussion, page 17, line 358. It should read: "Our results suggested that, in patients under non-opioid anesthesia…"

Author response: We do think this is good suggestion, which will make our paper more clarity. We have modified it (Lines: 389, marked with yellow).

17. Comments. Discussion, page 19, limitations paragraph, line 391. It should read: "Fourth, our study was a single-institution study, small sample size, and some protocol amendments were performed. More in details, no data on PI were reported since PI can be only used in conscious patients and the serum potassium concentration was not recorded, because some patients were given potassium supplementation, which potentially results in a selection bias. Fifth, we cannot perform an intention-to-

treat analysis including the 3 patients excluded after randomization since we did not follow up these patients. Further studies will be needed to assess the long-term benefits of the avoidance of opioid use in lung cancer patients, as well as to assess the benefits of increasing survival."

Author response: Thank you very much for revising this part. You have given me a more rigorous understanding of English wording and sentence making. According to your comment, we have rewritten this part, see line 425-437 and marked with yellow.

18. Comments. Conclusions, line 402-405. It should read: "OFA with dexmedetomidine and sevoflurane plus TPVB appears to be feasible for the management of intraoperative nociception in ASA I-II, 18-65 years old, non-obese patients undergoing VATS for lung cancer. However, a deeper sedation level was produced in OFA than in OA for the achievement of an optimal balance of analgesia-nociception."

Author response: Thank you for underlining this deficiency. This section was revised and modified according to the suggestion (Lines:440-442, marked with yellow).

19. Comments. Manuscript. The language quality must be improved. We advise that you seek assistance from a colleague or have a professional editing service.

Author response: Thank you for your careful review. We are deeply sorry for the inconvenience they caused in your reading. The manuscript has been thoroughly

revised and rewritten by a native English speaker, so we hope it can meet the journal's standard. The modified details have been marked in yellow.

20. Comments. Please review your reference list to ensure that it is complete and correct. If you have cited papers that have been retracted, please include the rationale for doing so in the manuscript text, or remove these references and replace them with relevant current references. Any changes to the reference list should be mentioned in the rebuttal letter that accompanies your revised manuscript. If you need to cite a retracted article, indicate the article's retracted status in the References list and also include a citation and full reference for the retraction notice.

Author response: Thank you for your reminder, we have re-checked the references, and the changes are marked in yellow.

Note: The authors of this article, Nuoya Chen and Bingsha Zhao, have moved to Hengshui People's Hospital and Tianjin Chest Hospital due to their job changes. We have revised their current addresses (see Title page),

Thank you for your careful review. We really appreciate your efforts in reviewing our manuscript. We wish good health to you, your family, and community. Your careful review has helped to make our study clearer and more comprehensive.
