# Supplementary material for: Opioid-free anesthesia compared to opioid anesthesia for lung cancer patients undergoing video-assisted thoracoscopic surgery: A randomized controlled study
Source: PLoS One. 2021 Sep 23;16(9):e0257279. doi: 10.1371/journal.pone.0257279 (PMC8460000; doi:10.1371/journal.pone.0257279)
Supplement: Attachment — Submitted filename: Rebuttal Letter.docx [file pone.0257279.s005.docx]

Dear Editor:

On behalf of my co-authors, we thank you very much for giving us an opportunity to revise our manuscript, we appreciate editor and reviewers very much for their positive and constructive.

Comments and suggestions on our manuscript entitled ‘Lower sedation index and raised blood glucose with opioid-free anesthesia compared to opioid anesthesia for lung cancer patients undergoing video-assisted thoracoscopic surgery: a prospective, randomized, controlled study’(PONE-D-20-27554) are all valuable and immensely helpful for revising and improving our paper, as well as the important guiding significance to our research. We have studied reviewer’s comments carefully and have made revision which marked in yellow in the paper. We have tried our best to revise our manuscript according to the comments.

We would like to express our great appreciation to you and reviewers for comments on our paper. We would be glad to respond to any further questions and comments that you may have. Looking forward to hearing from you.

Thank you and best wishes to New Year.

Yours sincerely,

Xuelian Zhao

31/12/2020

Here is our response to academic editors.

1. Comments. Please ensure that your manuscript meets PLOS ONE's style requirements, including those for file naming.

 Author response: We are sorry that in the submitted manuscription, the format of the paper does not meet the requirements of PLOS. We have modified it in accordance with the PLOS ONE style templates.

2. Comments: a. Please amend your current ethics statement to include the full name of the ethics committee/institutional review board(s) that approved your specific study.
 b. Once you have amended this/these statement(s) in the Methods section of the manuscript, please add the same text to the “Ethics Statement” field of the submission form (via “Edit Submission”).
Author response: We are sorry for our negligence of the full name of the ethics committee. We have added the full name of the ethics committee in the method section and online submission (Lines: 104-105, marked with yellow).

3. Comments: Please provide additional details regarding participant consent. In the ethics statement in the Methods and online submission information, please ensure that you have specified what type you obtained (for instance, written or verbal, and if verbal, how it was documented and witnessed).

 Author response: Thank you for pointing this out. We have stated in the method section that the patient can only be accepted to the study after signing the informed consent form (Lines: 110, marked with yellow).

4. Comments: In your Methods section, please ensure you explain any deviations from your original protocol.

Author response: Thank you for pointing this out. We have stated in the method section that the investigator should explain to the patient the difference between the experimental method and the original protocol, and then sign the informed consent form after obtaining the patient’s consent before enrolled into the study (Lines: 109-110, marked in yellow).

5. Comments: We noted several instances of p < 0.000 in your manuscript. To comply with PLOS ONE submission guidelines, please report exact p-values for all values greater than or equal to 0.001. P-values less than 0.001 may be expressed as p < 0.001.

Author response: Thank you for pointing this out. We have made corrections in the results section.

6. Comments: Thank you for stating the following financial disclosure:

"No"

At this time, please address the following queries:

Please clarify the sources of funding (financial or material support) for your study. List the grants or organizations that supported your study, including funding received from your institution.

State what role the funders took in the study. If the funders had no role in your study, please state: “The funders had no role in study design, data collection and analysis, decision to publish, or preparation of the manuscript.”

If any authors received a salary from any of your funders, please state which authors and which funders.

If you did not receive any funding for this study, please state: “The authors received no specific funding for this work.”

Please include your amended statements within your cover letter; we will change the online submission form on your behalf.

Author response: Thank you for point this out. All of authors received no specific funding for this work. We have changed the online submission on our behalf.

7. Comments: Thank you for stating the following in your Competing Interests section:

"No"

Please complete your Competing Interests on the online submission form to state any Competing Interests. If you have no competing interests, please state "The authors have declared that no competing interests exist.", as detailed online in our guide for authors at [http://journals.plos.org/plosone/s/submit-now](about:blank)

 This information should be included in your cover letter; we will change the online submission form on your behalf.

Please know it is PLOS ONE policy for corresponding authors to declare, on behalf of all authors, all potential competing interests for the purposes of transparency. PLOS defines a competing interest as anything that interferes with, or could reasonably be perceived as interfering with, the full and objective presentation, peer review, editorial decision-making, or publication of research or non-research articles submitted to one of the journals. Competing interests can be financial or non-financial, professional, or personal. Competing interests can arise in relationship to an organization or another person. Please follow this link to our website for more details on competing interests: [http://journals.plos.org/plosone/s/competing-interests](about:blank)

Author response: Thank you for point this out. All of authors received no specific funding for this work. We have changed the online submission on our behalf.

8. Comments: Please include captions for your Supporting Information files at the end of your manuscript, and update any in-text citations to match accordingly. Please see our Supporting Information guidelines for more information: [http://journals.plos.org/plosone/s/supporting-information](about:blank).

 Author response: Thank you for point this out. We have corrected.

List of Responses

Reviewer #1: I would like to thank the authors for this RCT comparing OFA to OA in lung cancer surgery. The authors show that OFA is associated to non-inferior analgesia, despite a deeper level of sedation and raised blood glucose.

##### Dear Reviewer: Thank you for your letter and for the comments concerning our manuscript entitled “Lower sedation index and raised blood glucose with opioid-free anesthesia compared to opioid anesthesia for lung cancer patients undergoing video-assisted thoracoscopic surgery: a prospective, randomized, controlled study” (PONE-D-20-27554). Those comments are all valuable and very helpful for revising and improving our paper, as well as the important guiding significance to our research. We have studied the comments carefully and have made corrections which we hope meet with approval. Revised portion were marked in yellow in the paper. The main corrections in the paper and the responses to the reviewer’s comments are as flowing:

**Responses to the reviewer’s comments:**

1. Comments：Introduction
Page 3 L 64: please, detail that OFA provides better analgesia in the postoperative period. Intraoperative analgesia is actually the aim of this paper. This will help not making confusion.
See for reference
Analgesic impact of intra-operative opioids vs. opioid-free anaesthesia: a systematic review and meta-analysis. Frauenknecht J, Kirkham KR, Jacot-Guillarmod A, Albrecht E.Anaesthesia. 2019 May;74(5):651-662. doi: 10.1111/anae.14582. Epub 2019 Feb 25. PMID: 30802933 Free article.

Author response: We do think this is a good suggestion, which will make paper clearer and well reflect the content of the article. We have rewritten the introduction and described that OFA can improve postoperative analgesia in patients undergoing open thoracic surgery, see Lines 53-59 with yellow labels.

Thank you very much for the literature, see reference 19 (yellow-marked).

2. Comments：Page 4 line 86: merge this part to the initial part of introduction; this will keep all the details on OFA together. I also think the authors can shorten this part. They do not need to detail on paravertebral block or ketorolac or Dexmethomidine, but just briefly explain that “OFA encompasses different strategies (including dexmethomidine, ketorolac and so on)…and the use of regional anesthesia to act on different components of pain pathways”. They may cite some articles explaining the topic. For reference:
Opioid free anesthesia: evidence for short and long-term outcome.
Bugada D, Lorini LF, Lavand'homme P.Minerva Anestesiol. 2020 Aug 4. doi: 10.23736/S0375-9393.20.14515-2. Online ahead of print.PMID: 32755088
This is not mandatory but will significantly help the reader and make the reading more fluent.

Author response: As suggested by the reviewed, we have deleted the description of anesthetics in OFA strategies in the introduction and put this part into the discussion in detail.
Thank you very much for the literature, see reference 24 (yellow-marked).

3. Comments：Methods:
line 112 page 5: I guess thy mean “pregnant”

Author response: We are very sorry for our incorrect writing and have made correction “pregnant”.

4. Comments：
Please, better detail on allocation and blinding. This is important. How do you assign patients to their group and how you keep the investigators unaware of their assignment? How data collectors register data on awareness and nociception during general anesthesia (I guess they are in the OR) without noticing that the patient is infused with one drug or the other?

Author response: We do think these are good questions. We have rewritten the allocation and blinding in detail lines from 128-137 (yellow-marked). Because the management of OFA and OA are different, it is not possible to blind the anesthesiologist and the recorder during the operation.

5. Comments：Page 8 line 162
How do you maintain Sevoflurane ? Basing on Et%? Sevoflurane may interfere with hypnosis if sevoflurane dose is not standardized, how can we make sure that different levels of hypnosis between OFA and OA patients are given by the treatment rather than by different sevoflurane concentrations? Please, better detail on this point.

Author response: We do think these are good questions. We have added an analysis of the effect of sevoflurane on the sedation index in the discussion section (Lines: 355-357).

Sevoflurane affects the depth of sedation and could be monitored by WLI (Jian-Wen Zhang, Zhi-Gan Lv, Ying Kong, Chong-Fang Han, Bao-Guo Wang. Wavelet and pain rating index for inhalation anesthesia: A randomized controlled trial. World J Clin Cases 2020 November 6; 8(21): 5221-5234; Reference 39, yellow-marked). Sevoflurane is an important anesthetic for our study. The experienced anesthesiologists adjust the sevoflurane according to the operation process and various indicators of the patient. Since the consumption of sevoflurane used for anesthesia cannot be accurately calculated, we only record the exhaled concentration of sevoflurane at the end of the operation, and there is no significant difference (*P* >0.05). Therefore, we cannot determine whether intraoperative consumption of sevoflurane is one of the reasons for the difference in WLI between the two groups. This is also the limitation of this article, which we have already described in the discussion section.

6. Comments：Please, better describe in the text the sampling times used in the study: in the figure captions only it is not enough. This point should be clearer from the very beginning to the reader.

Author response: We are deeply sorry for the serious omission and have made correction which we hope will meet with approval (Lines195-201, which marked in yellow).

7. Comments：Results:
7.1. Page 9 line 193: please, remove the timeframe. It is already in the methods.

Author response: We do think this is a good suggestion, and the timeframe has been deleted from Results.

7.2. Page 12 line 247: significant “difference” , I guess.

Author response: We do think this is a good suggestion. It may be that we did not express it clearly. Line 247 means that there is no statistical difference in blood glucose levels of patients under opioid anesthesia at different time points. We have made corrections.

7.3. How do the authors define time for extubation? Any specific parameter?

Author response: We do think these are good questions. In our study, extubation time refers to the time from intravenous bolus of neostigmine to the removal of the tracheal intubation. The indications for tracheal intubation removal are controlled by the anesthesiologist.

8. Comments：Discussion:
My main point: this work challenges the idea that OFA does not provide enough analgesia comparing to OA. Part of the criticism on OFA comes from this wrong assumption, that patients will have pain during surgery because they don’t get opioids. This is false, and this work specifically demonstrate it, with an objective measure of analgesia, and as a primary outcome.
So, please, highlight this point. This is good. Let state that this is the first study giving any evidence that intraoperative analgesia is comparable to OA; this will reinforce the concept that OFA is not denying anything to the patients, as already evident for postoperative analgesia. See for reference (Analgesic impact of intra-operative opioids vs. opioid-free anaesthesia: a systematic review and meta-analysis. Frauenknecht J, Kirkham KR, Jacot-Guillarmod A, Albrecht E. Anaesthesia. 2019 May;74(5):651-662. doi: 10.1111/anae.14582. Epub 2019 Feb 25. PMID: 30802933 Free article).

Author response: We agree with the reviewer’s assessment. Accordingly, we have rewritten discussion and reiterated that the method of observing PTI readings was used for the first time to achieve real-time monitoring of the depth of analgesia during the operation, which confirmed that there was no difference between opioid anesthesia and opioid anesthesia (Lines: 309-311，319-333).

9. Comments：

9.1. Page 13 line 278-284: out of context. I would erase this part.

Author response: We do think these are good suggestion. We have deleted and rewritten this part (Lines:292-335).

9.2. Why do they not use ketamine. Ketamine is a cornerstone of OFA. Is it because it may interfere with sedation measurement? Please, discuss this point.

Author response: We do think these are good questions. Ketamine is a cornerstone of OFA, but ketamine can cause hallucinations and nightmares after surgery, so it is rarely used in ours hospital. Another important reason is that our hospital cannot order ketamine, there is no ketamine for clinical anesthesia.

9.3. Dexmedethomidine, as well as ketamine does, may give “false” or “apparent” levels of sedation because of their action of EEG.. the authors state it in the discussion, but then they stop, only citing a reference. Please, better detail this point for the reader, because it is extremely interesting in the global evaluation of the study

Author response: Thank you for pointing this out. We have detail in discussion (Lines:348, 356-359, yellow-marked)
10. Comments：

10.1. Page 15: line 317-320: not clear what do the authors mean

Author response: Thank you for pointing this out. We have rewritten this part, see lines 385-389 for details (yellow-marked).

10.2. Line 321-323: why is this a limitation?

Author response: We do think these are good questions. Because single-lung airway management in VATS generally uses dual-lumen endotracheal catheters, the use of single-lumen endotracheal catheters plus bronchial blocker is rare. Moreover, the stress response of double-lumen endotracheal tube intubation is more severe than that of single-lumen tube.

11. Comments：Conclusion:
I suggest some changes in the message the authors want to give. I suggest not to merely repeat the results
1- OFA provides comparable analgesia (see before)
2- They measure an higher level of sedation but: a) this is a secondary outcome; b) doses of sevoflurane may be adjusted (in future studies) when deeper sedation should not be achieved c) dexmedethomidine, as well as ketamine does, may give “false” or “apparent” levels of sedation because of their action of EEG. Is the deeper level measured really deeper? Further study with sedation as primary endpoint are needed.
3- Glucose is raised but it is probably because of the drugs and not because of surgical stress. So it does not probably mean that OFA does not reduce stress, but only that we need to stay aware and keep higher attention in diabetic patients.

Author response: As suggested by the reviewer, we have rewritten the conclusions. In our OFA regimen, sevoflurane plays an important role. If the intraoperative consumption of sevoflurane can be clarified, it can be more clearly explained whether sevoflurane is the cause of lower sedation index in OFA except dexmedetomidine. Therefore, in the new study, we calculate the consumption of sevoflurane in different time intervals and the number of changing concentrations of sevoflurane during the operation.

##### Once again, Special thanks to you for your good comments and suggestions.

Reviewer #2: I find the original manuscript interesting and it appears to be methodically correct and has not noticed any significant critical issues.
The title is appropriate to the content, informative, concise and clear.
The article deals with a very interesting topic in a comprehensive way and without structural shortcomings.
The data provided is complete and well described and the statistical analysis is absolutely well developed.
The study's objectives and conclusions are clear.
The references are sufficient as a scientific basis for the type of manuscript.

##### Dear Reviewer: Thank you for your letter and for the comments concerning our manuscript entitled “Lower sedation index and raised blood glucose with opioid-free anesthesia compared to opioid anesthesia for lung cancer patients undergoing video-assisted thoracoscopic surgery: a prospective, randomized, controlled study” (PONE-D-20-27554). Your comment is valuable and very helpful for revising and improving our paper, as well as the important guiding significance to our research. We have studied the comments carefully and have made corrections which we hope meet with approval. Revised portion were marked in yellow in the paper.

**Responses to the reviewer’s comments:**

1. Comments：I recommend to the authors a more detailed description of the paravertebral block (which needle was used? Which ultrasound probe? In plane or out of plane technique?)

Author response: We do think this is a good suggestion. According to your suggestions, we have perfected the operation of thoracic paraspinal block under ultrasound guidance (Lines: 184-188, marked in yellow).

##### Once again, Special thanks to you for your good comments and suggestions.

Reviewer #3: I read with great interest the study entitled “Lower sedation index and raised blood glucose with opioid-free anesthesia compared to opioid anesthesia for lung cancer patients undergoing video-assisted thoracoscopic surgery: a prospective, randomized, controlled study ».
This is an interesting study because it represents a validation of intraoperative analgesia with OFA in comparison to standard opioid anesthesia. To date, we do not have such data.

##### Dear Reviewer: Thank you for your letter and for the comments concerning our manuscript entitled “Lower sedation index and raised blood glucose with opioid-free anesthesia compared to opioid anesthesia for lung cancer patients undergoing video-assisted thoracoscopic surgery: a prospective, randomized, controlled study” (PONE-D-20-27554). Those comments are all valuable and very helpful for revising and improving our paper, as well as the important guiding significance to our research. We have studied the comments carefully and have made corrections which we hope meet with approval. Revised portion were marked in yellow in the paper. The main corrections in the paper and the responses to the reviewer’s comments are as flowing:

**Responses to the reviewer’s comments:**

1. Comments：• The title must be changed because it does not reflect the objective of the present study. According to authors “In this prospective, randomized trial we tested the primary hypothesis that protocol- intraoperative OFA would provide efficacious intraoperative analgesia compared to the opioid-based GA with PTI monitoring”. The results were that you did not demonstrate any difference between OFA and non-OFA groups in term of PTI.

Author response: We do think this is a good suggestion, which will make the title clearer and well reflect the content of the article. We have modified title to “The equal analgesic index and lower sedation index with opioid-free anesthesia compared to opioid anesthesia for lung cancer patients undergoing video-assisted thoracoscopic surgery: a prospective, randomized, controlled study”.

2. Comments：• I would suggest to use non-opioid balanced general anesthesia instead of opioid free anesthesia throughout the manuscript. In the title, you can use OFA but after do not use this term. OFA is a misleading term because people believe that you don’t treat pain.

Author response: We do think this is a good suggestion, which will help us make the paper more rigorous.

It can be seen from literatures that there are ‘non-opioid anesthesia’ and ‘opioid-sparing anesthesia’ similarly to ‘opioid-free anesthesia’. Currently what can be accepted so far is ‘opioid-free anesthesia’. Opioid-free anesthesia (OFA) is an emerging technique summarized as “multimodal anesthesia” to supply for OBA. As one drug will not replace opioids, it is the association of drugs and/or techniques that allows a good quality general anesthesia with no need for opioids. A rational strategy implies: 1) combining anti-nociceptive agents to target different circuits involved in nociceptive transmission, 2) monitoring nociception, 3) explicitly using sedative effects of anti-nociceptive agents to reduce the doses of hypnotic agents and 4) providing prolonged pain control.

Dario BUGADA, et al. Opioid free anesthesia: evidence for short and long-term outcome. [J] Minerva Anesthesiology 2020 Aug 04.

J. Frauenknecht, K. R. Kirkham, A. Jacot-Guillarmod and E. Albrecht. Analgesic impact of intra-operative opioids vs. opioid-free anaesthesia: a systematic review and meta-analysis.[J] Anaesthesia 2019, 74, 651–662

HeleneBeloeil. Opioid-free anesthesia. [J]. Best Practice & Research Clinical Anaesthesiology. 2019;[33(3](https://www.sciencedirect.com/science/journal/15216896/33/3)): 353-360

Therefore, in the revision of this article, we use OFA as a conceptual term, while some content descriptions we use non-free anesthesia.

3. Comments：• In the introduction section, the authors should better focus on cardiac and thoracic surgery. You have published OFA studies on this topic: Guinot PG, Spitz A, Berthoud V, Ellouze O, Missaoui A, Constandache T, Grosjean S, Radhouani M, Anciaux JB, Parthiot JP, Merle JP, Nowobilski N, Nguyen M, Bouhemad B. Effect of opioid-free anaesthesia on post-operative period in cardiac surgery: a retrospective matched case-control study. BMC Anesthesiol. 2019 Jul 31;19(1):136. doi: 10.1186/s12871-019-0802-y. PMID: 31366330; PMCID: PMC6668113. Bello M, Oger S, Bedon-Carte S, Vielstadte C, Leo F, Zaouter C, Ouattara A. Effect of opioid-free anaesthesia on postoperative epidural ropivacaine requirement after thoracic surgery: A retrospective unmatched case-control study. Anaesth Crit Care Pain Med. 2019 Oct;38(5):499-505. doi: 10.1016/j.accpm.2019.01.013. Epub 2019 Feb 5. PMID: 30731138. These studies are interesting because (even they are retrospective ones) they add data to improve the discussion on your finding. They have been performed on thoracic and mini-invasive cardiac surgery that are quite similar to your population. And they use different OFA protocol. You should discuss your protocol in regard of those published.

Author response: We have added the suggested content to the manuscript on introduction (Reference 14 and 15, yellow-marked). We have rewritten the introduction and described in detail that OFA can improve postoperative analgesia in patients undergoing open thoracic surgery, see Lines 53-59 with yellow labels.

4. Comments：• How was managed remifentanil dose during the surgery ?

Author response: We do think this is a good question.

The anesthesia of all patients in this study was completed by the experienced anesthesiologist. The anesthesiologist adjusts the remifentanil infusion rate during the operation according to the progress of the operation, the patient’s blood pressure and heart rate, and his own experience. We recorded the consumption of remifentanil throughout the operation.

5. Comments：• When did you perform TPVB? At the beginning or at the end of the surgery? Because if you performed the TPVB at the beginning of the surgery, you cannot exclude an effect of TPVB on your results.

Author response: We do think this is a good question.

In our study, after the anesthesia induction, all patients were placed in a lateral position and received single-injecting TPVB guided by an ultrasound machine at T5-6 with ropivacaine (0.5%, 15 ml). In our department of anesthesia, all patients undergoing thoracoscopic lobectomy received general anesthesia plus thoracic paravertebral block anesthesia (before beginning of surgery with different local anesthetic). Because we cannot rule out the analgesic effect of paravertebral block, all patients in this study received paravertebral block with 0.5%ropivacaine 15 ml.

We have discussed effect of TPVB on our result (Lines334-343).

6. Comments：• Table 2 : switch PRI with PTI. Please add definitions of abbreviations in the legend for all tables and figures.

Author response: We are very sorry for the serious omission and have made correction (marked in yellow) which we hope will meet with approval.

7. Comments: Even the result on BWI is statically significant, I don’t believe that it is clinically relevant. The values of BWI in the two groups were within the fixed ranges. This effect can be explained by the sedation effect of dexmedetomidine. It would be of interest to have values of end tidal sevoflurane during the surgery (T3 to T5). This is a major issue because you demonstrated a difference of sedation level.

Author response: We agree with the reviewer’s assessment.

Our results showed that the BWI readings in group OFA were significantly lower than those of group OA (55.2: 59.5 at T3), however, the BWI readings were in normal depth of sedation. We also made a statement in the discussion section (Lines: 364-365).

8. Comments：• Discussion section: don’t discuss the validity of the PTI. You should better focus on the effectiveness of your OFA protocol regarding analgesia during the surgery. We do not have one type of OFA protocol. Some groups use lidocaine, other mix lidocaine and dexmedetomidine… You should discuss the bias of using TPVB if you perform this block at the beginning of the surgery. You should discuss how you manage sedative drugs during the surgery, thus the low clinical impact of lower sedation level with OFA. You can use recent publications evaluating the impact of a low vs high level of sedation during anesthesia. The results on blood glucose values are interesting because I observe the same finding at bedside in major surgery being performed with OFA. I also note higher lactate values but you did not demonstrate this because you have studied non major thoracic surgery. You should shorten the corresponding paragraph.

Author response: We do think these are excellent suggestions. We have deleted the validity of the PTI and rewritten this part (Lines: 319-343).

Our study results show that there is no significant difference in lactic acid levels at the time points between the two groups, but the lactic acid values after extubation of the two groups are significantly higher than the preoperative basic values.

9. Comments：• Limitation section: I do not agree with your assessment “Third, it was impossible to calculate the consumption of sevoflurane during the operation, and thus the end-expiratory sevoflurane concentration at the end of surgery in both groups was compared.” It is possible to have value. Do you have a data base? Are you able to get these values?

Author response: We do think these are good questions.

We observed that during the entire period of anesthesia, the senior anesthesiologists need to frequently adjust the sevoflurane inhalation concentration and oxygen flow according to the progress of the operation and the patient's vital signs. Therefore, the amount of sevoflurane cannot be accurately calculated (Consumption of inhaled anesthetics (ml)/hour = constant λ × volatile tank scale (%) × fresh gas flow (L/min). We only record the exhaled concentration of sevoflurane at the end of the operation, and there is no significant difference (P>0.05). Therefore, we cannot determine whether intraoperative consumption of sevoflurane is one of the reasons for the difference in WLI between the two groups. This is one of the limitations of this article. However, sevoflurane is an important anesthetic for our study. which affects the depth of sedation and could be monitored by WLI (Jian-Wen Zhang, Zhi-Gan Lv, Ying Kong, Chong-Fang Han, Bao-Guo Wang. Wavelet and pain rating index for inhalation anesthesia: A randomized controlled trial. World J Clin Cases 2020 November 6; 8(21): 5221-5234). Therefore, in future experiments, we plan to record the number and time of adjusting sevoflurane inhalation concentration in order to calculate the amount of sevoflurane.

10. Comments：• Outcomes (Item 6a) Completely defined pre-specified primary outcome measure including how and when it was assessed.
Incompletely. What was the primary objective? PTI or BWI ? Why did you perform sample size calculation only on PTI?

Author response: Thank you for pointing this out. PTI was the primary objective of our study. The purpose of this study is to observe whether no opioid anesthesia can achieve the equal analgesic effect of traditional opioid anesthesia. Therefore, we set the analgesia index as the primary observation index and calculate the sample size based on the analgesia index.

11. Comments：• Allocation concealment (Item 9)
Mechanism used to implement random allocation sequence (such as sequentially numbered containers), describing any steps taken to conceal the sequence until interventions were assigned
Did you stratify randomization? If yes can you develop?
Author response: We do think this is a good question. We did not perform stratified randomization during random allocation. But in our new study, we are stratified and randomized whether you have diabetes.

12. Comments：• Harms (Items 19)
All important harms or unintended effects in each group
Could you provide data on safety ? bradycardia hypotension hypertension tachycardia…..

Author response: We do think this is a good question. Yes，we recorded the incidence of complications in this experiment, such as sinus brachytherapy, hypertension, hypotension, etc. There was no statistical difference between the two groups.

##### Once again, Special thanks to you for your good comments and suggestions.

##### Reviewer #4: Comments on Manuscript # PONE-D-20-27554 from An and colleagues. The authors propose a randomized study comparing opioid free anesthesia (OFA) using dexmedetomidine infusion with opioid based anesthesia (OA) using fentanyl and remifentanil infusion in patients scheduled for thoracoscopic lung cancer resection. All the patients received paravertebral block. The authors used intraoperative monitors to assess intraoperative nociception and depth of anesthesia (general anesthesia with sevoflurane).

Dear Reviewer:
Thank you for your letter and for the comments concerning our manuscript entitled “Lower sedation index and raised blood glucose with opioid-free anesthesia compared to opioid anesthesia for lung cancer patients undergoing video-assisted thoracoscopic surgery: a prospective, randomized, controlled study”(PONE-D-20-27554). Those comments are all valuable and very helpful for revising and improving our paper, as well as the important guiding significance to our research. We have studied the comments carefully and have made corrections which we hope meet with approval. Revised portion were marked in yellow in the paper. The main corrections in the paper and the responses to the reviewer’s comments are as flowing:

**Responses to the reviewer’s comments:**

##### Introduction and abstract: 1. Comments：- the authors should not use “pain intensity or pain relief” in anesthetized patients but should use the term “intraoperative level of nociception or control of intraoperative nociception”.

Author response: We do think this is a good suggestion, which will make our paper clearer and more well-organized. We have made changes in relevant places in the article.

2. Comments：- Also the abstract “results” mention T1….T2-T5 ? without any explanation in the methods. Please detail.

Author response: We are very sorry for the serious omission and have made correction which we hope will meet with approval (Lines194-201, which marked in yellow).

3. Comments：- The authors seem to rely on the monitoring they use to assess both anesthesia depth and nociception by analysis of EEG. The measures of all the monitors currently available to assess intraoperative nociception are subject to caution. More details about the monitor used here are mandatory – only one single reference (#17) is not enough.

Author response: We do think this is a good suggestion, which will make our paper more credible. We have added three more literatures. (#18: Hao Kong HZ. Research progress of noxious pricking stimulation and analgesic water level monitoring during operation. Journal of Clinical Anesthesiology. 2020;36(06):612-5; #21: Zhen Su LA, Yang Zhang, Lianhua Chen. The clinical value of analgesia index in evaluating the degree of analgesia during general anesthesia. Chinese Journal of Applied Physiology. 2018;34(5):461-3; #22: Qingqing Zhu, Wei Yue, Jinnan Jiang, Miao Zhang,JianxinＹang. Application of target-controlled infusion of remifentanil guided by pain threshold index in laparoscopic subtotal gastrectomy. J Clinical Anesthesiology, 2020;36(7):634-7).

4. Comments：- Introduction should be re-written. The authors have mixed all the potential benefits of OFA, from reduction of postoperative opioid side effects to chronic pain to cancer recurrence. Please focuse on OFA in the context of the present study i.e. control of intraoperative nociception and stress response to surgery.

Author response: We agree the reviewer’s suggestion which will make our paper clearer and well reflect the content of the article. We have rewritten the introduction and described in detail the effects of OFA on intraoperative surgical stress response and nociception in the discussion section.

Methods:
5. Comments：- The authors mention PTI values… please state if these are the values from the Company or the values from the scientific literature. Add more references about the intraoperative use of this monitor.

Author response: We do think this is a good suggestion, which will make our paper more credible. The values of PTI and values of BWI were from the Company（Yibin Wu. Extraction of objective and quantitative indexs of pain, anxiety, depression and other brain function states from electroencephalogram. China Medical Engineering, 2017;25(4):1-7）.

6. Comments：- When was the paravertebral block realized: end of anesthesia induction or end of surgery?

Author response: We are very sorry for the serious omission. After the anesthesia induction, all patients were placed in a lateral position and received single-injecting TPVB.

7. Comments：- Provide more details about primary and secondary outcomes e.g. blood gas measuring glucose level, lactic acid….

Author response: As the reviewer’s suggestion, we have added and improved the details about primary and secondary outcomes (Lines 194-201 and yellow-marked).

8. Comments：- More the different times of repeated measures to assess both the primary and secondary outcomes should be detailed here in the methods.

Author response: Thank you for pointing this out, which will make our paper more distinct. We have added and improved the details about primary and secondary outcomes (Lines 194-201 and yellow-marked).

9. Comments：- Too bad that the authors who state in introduction that OFA may help to control postoperative pain did not assess pain values and analgesics in patients in the recovery room

Author response: We do think this is a good suggestion, which will make our paper more distinct. Because the post-anesthesia care unit (PACU) of our hospital was rebuilt in 2018, we did not record the patient's PTI readings and BWI readings in the PACU. According to our original protocol, the patients in group OFA were treated with dexmedetomidine plus ketorolac in PCIA for postoperative analgesia and patients in group OA were received dezocine and ketorolac in PCIA for postoperative analgesia. Our results showed that there was no statistical difference in PTI values and BWI values between the two analgesic regimens. Because the postoperative analgesic of the OA group was different from intraoperative opioids, we did not discuss in this article.

10. Comments：- Statistical analysis is provided but power calculation is quite unclear because only mean PTI is reported; please describe the confidence interval or standard deviation.

Author response: We agree with the reviewer’s suggestion, which will make our paper more distinct. We have added standard deviation of PTI.

11. Comments：- Please add in tables 2 and 3 what is statistically significant from what – not clear for the reader or better provide figures (some figures are already displaying the results of the tables)

Author response: As suggested by the reviewer, we have improved the statistically significant of table 2 and table 3 highlighted with yellow marker.

12. Comments：Discussion: is confusing partly because your primary and secondary outcomes are not clearly describe in introduction and methods. You want to compare the control of intraoperative nociception, depth of anesthesia and stress response between two techniques of anesthesia (OFA versus OA).

Author response: We do think this is an excellent suggestion. We have rewritten introduction and methods, to clear that the primary outcome is PTI, and the secondary outcomes are WLI and blood glucose level and discussion part.

##### Once again, Special thanks to you for your good comments and suggestions.

Reviewer #5: Study appears well designed.

##### Dear Reviewer: Thank you for your letter and for the comments concerning our manuscript entitled “Lower sedation index and raised blood glucose with opioid-free anesthesia compared to opioid anesthesia for lung cancer patients undergoing video-assisted thoracoscopic surgery: a prospective, randomized, controlled study” (PONE-D-20-27554). Those comments are all valuable and very helpful for revising and improving our paper, as well as the important guiding significance to our research. We have studied the comments carefully and have made corrections which we hope meet with approval. Revised portion were marked in yellow in the paper. The main corrections in the paper and the responses to the reviewer’s comments are as flowing:

##### Responses to the reviewer’s comments:

##### 1. Comments: “Limitations should include single institution study too”.

##### Author response: It is really true as Reviewer suggested that the single institution study is the limitation of our study was added (Lines: 404-405, yellow-marked).

##### 2. Comments: “Paper has grammatical errors and spacing mistakes between sentences which need to be addressed”.

##### Author response: We are very sorry for our incorrect writing and have made correction.

##### Once again, Special thanks to you for your good comments and suggestions.
