# Supplementary material for: Opioid-free anesthesia compared to opioid anesthesia for lung cancer patients undergoing video-assisted thoracoscopic surgery: A randomized controlled study
Source: PLoS One. 2021 Sep 23;16(9):e0257279. doi: 10.1371/journal.pone.0257279 (PMC8460000; doi:10.1371/journal.pone.0257279)
Supplement: Attachment — Submitted filename: Rebuttal Letter 2.0.docx [file pone.0257279.s006.docx]

Dear Dr Alessandro Putzu:

Thank you very much for your email.

On behalf of my co-authors, we thank you very much for giving us an opportunity to revise our manuscript, we appreciate editor and reviewers very much for their positive and constructive.

Comments and suggestions on our manuscript entitled ‘Lower sedation index and raised blood glucose with opioid-free anesthesia compared to opioid anesthesia for lung cancer patients undergoing video-assisted thoracoscopic surgery: a prospective, randomized, controlled study’(PONE-D-20-27554) are all valuable and immensely helpful for revising and improving our paper, as well as the important guiding significance to our research. We are truly grateful to the reviewers’ critical comments and thoughtful suggestions. According to these comments and suggestions, we have made careful modifications on our manuscript. In the revised version, all changes made to the text descriptions are in yellow color in the revised version.

We would be glad to respond to any further questions and comments that you may have. Looking forward to hearing from you.

Thank you and best wishes.

Yours sincerely,

Xuelian Zhao

22/3/2021

Here is our response to academic editors.

1. Comments. The manuscript should be conformed to all points reported in the CONSORT guidelines.

Author response: We are sorry that in the submitted manuscription, the format of the paper does not meet the requirements of the CONSORT guidelines. We have modified it in accordance with the CONSORT guidelines.

2. Comments: Title. I suggest to use a shorter, clearer and objective title, such as: “Opioid-free anesthesia compared to opioid anesthesia for lung cancer patients undergoing video-assisted thoracoscopic surgery: a randomized controlled study”.

Author response: We do think this is a good suggestion, which will make title clearer and well reflect the content of the article. We have rewritten the title with yellow labels.

3. Comments: Abstract. The use of T1-2-3-4-5-6 time points are unclear. Readers should be able to understand the time points when reading the abstract. Please increase clarity. I suggest to avoid the use of these acronyms in the abstract.

Author response: We do think this is a good suggestion, which will make our paper clearer and easier to understand. We have made changes in relevant places in the abstract marked with yellow.

4. Comments: Abstract. You should report that you included only ASA 1-2, 18-65 years old, non-obese, patients.

Author response: We do think this is a good suggestion, which will make our paper clearer and more rigorous. We have made changes in relevant places in the abstract marked with yellow.

5. Comments: Methods. You registered the protocol at the end of the study (Date of Last Refreshed on：2020/7/5 22:02:01). This is a major limitation.

Author response: Thank you for pointing this out. Because the original protocol only said to detect arterial blood gas, we added the protocol on 2020/7/5, specifying that the blood gas detection items are PH, PaO_2_, lactic acid and blood glucose.

6. Comments: Registration. The study is registered on ChiCTR, not on clincialtrial. gov.

Author response: Thank you for pointing this out. For the clinical registration of this study, I consulted with the Scientific Research Department of our hospital and was informed that both https://www.clinicaltrials.gov/ in the United States and http://www.chictr.org.cn/index.aspx in China are both first-level registration platforms. And finally, we chose ChiCTR.

7. Comments: Primary outcomes. In the protocol (ChiCTR ) you reported 3 co-primary endpoints: pain evaluation, MAP, analgesic index, pain index. Please report in Methods why you changed the pre-defined outcome. All these outcome should be reported in the manuscript. This is a limitations to report in the discussion.

Author response: Thank you for pointing this out. In the end, we use the PTI as the only primary outcomes of this article, because this study mainly observes whether OFA can provide equivalent analgesia compared with traditional OA. According to the study protocol, we also recorded the intraoperative MAP and HR at different time points. We added MAP and HR data in the method and the results depending on your suggestions (Tab 3, Fig 2, marked with yellow). Pain index (PI range 0–100) is an objective and quantitative EEG pain assessment indicator developed. Its corresponding relationship with the VAS scale is as follows: (1) PI <10, no pain, corresponding to VAS scale 0–2; (2) PI = 10–15, pain threshold range, due to different individual pain thresholds, some people can feel pain, some people feel no pain, corresponding to VAS scale 3–4; (3) PI = 16–30, moderate pain, corresponding to VAS scale 5–7; (4) PI >30, severe pain, corresponding to VAS scale 8–10. PI can be used to objectively monitor the degree of pain in conscious patients after surgery. So, our paper does not have data on the pain index.

8. Comments: Secondary outcomes. In the protocol you reported as secondary outcomes: exhaust time, PONV, length of stay, PaO2, length of stay, lactic acid, pH, K, SpO2. Please report why you changed this pre-defined outcomes. Multiple outcomes correction should be used.

 Author response: Thank you for pointing this out. According to the study protocol, we recorded the remove drainage tube time, dizzy, PONV, exhaust time, hospital discharge time, pH, SpO_2_ and PaO_2_. We added these items in the method and the results (Tab 2, Tab 4, Fig 3, yellow marked) depending on your suggestions. We found that some patients had hypokalemia before the operation, and this part of the patients received intravenous potassium chloride infusion, so we did not record intraoperative blood potassium value of blood gas.

9. Comments: Outcomes. Time points (T1-6) should be consistent between primary and secondary outcomes. For example, timepoint T6 for primary outcome is equal to T4 for secondary outcome. Unclear, should be fixed.

Author response: Thank you for your suggestion. We have redefined the collection time point of the primary outcomes and the secondary outcomes. The time points of PTI, BWI, MAP and HR were T1-6 (T1: baseline values, T2: 10min after dexmedetomidine infusion (1μg kg^-1^), T3: after intubation, T4: 30min after one lung ventilation, T5: both lungs ventilation, T6: 5 min after extubation). The time points of blood gas were T_1-4_ (T_1_: baseline values, T_2_: 1h after surgery begun, T_3_: 2h after surgery begun, T_4_: 5 min after extubation).

10.Comments：Safety outcomes. Please report some major clinical outcomes. This should include in my opinion: mortality, length of hospital stay, ICU admission, reintubation, and respiratory insufficiency.

Author response: We do think this is a good suggestion, we have added safety results in the results (see Tab 2 and Line272-274, marked with yellow).

11.Comments: Outcomes. Please specify when and where you assessed pain after extubation (in the PACU? 15 minutes after extubation?)

Author response: Sorry for not specifying the time point of T6 and T_6_. The time point of T6 and T_6_ are 5 minutes after extubation in the operation room (Lines: 182, 186; yellow marked).

12.Comments: Sample size. You enrolled 100 patients, not 110 patients. 10 patients refused to participate.

Author response: We are deeply sorry for the error and have made correction marked with yellow (Lines: 204-205).

13. Sample size. The statement “We recruited 55 patients per group to minimize the chance of insufficient power, in case the observed variability was higher than expected” is incorrect since you enrolled 100 patients.

Author response: Thank you for pointing this out. This description is indeed inappropriate, and we have deleted this sentence.

14.Comments: Analysis. An intention to treat analysis (including n=100) should be also performed for at least major clinical outcomes. You should report why you did not use an ITT analysis.

Author response: We do think this is good question. The purpose of this study is to test whether the depth of analgesia without opioid anesthesia is equivalent to that of opioid anesthesia. ITT analysis is more suitable for superiority experiments. Therefore, we excluded three patients in accordance with the experimental protocol, and collected data from 97 patients.

15.Comments: All protocol changes should be reported in the manuscript and in the full form in the supplementary material, with reasons (see CONSORT checklist 3b)

Author response: We do think this is good suggestion. Since midazolam may affect the cognitive function of the elderly after surgery, we changed the experimental plan that all patients did not use midazolam before the induction of anesthesia. Before the patient signs the informed consent form, inform the patient of this change, and sign the informed consent form after the patient agrees (Lines: 97-99, marked with yellow). We have added those in the manuscript and in the protocol.

16.Comments: Methods. Please report which drugs were used or not used, and if allowed in both groups. For example: ketamine, clonidine, dexmedethomidine, magnesium, dexamethasone or corticoids, local anesthetics surgical infiltration, PONV prophylaxis etc.

Author response: We do think this is good suggestion. We add Table 1 according to the anesthesia plan and list the anesthetics involved in this study according to the anesthesia steps.

17.Comments: Figure 1. You screened 110 patients. Is it correct that no one met an exclusion criteria? There are errors?

Author response: We do think this is good question. 110 patients met the inclusion criteria and we have made it clear in the article marked with yellow.

18.Comments: Results. Does transfusion volume indicate blood transfusion or crystalloids? Unclear.

Author response: We do think this is good question. No blood transfusion in all patients during the operation. We have modified transfusion volumes in Table 1 to the fluid infusion volumes marked up yellow.

19.Comments: Discussion and Conclusions. You should report that you included only ASA 1-2, 18-65 years old, non-obese, patients. This do not allow the generalizability of the results.

Author response: We do think this is good suggestion, which will make our paper more rigorous. We have added only ASA 1-2, 18-65 years old, non-obese, patients in the discussion and conclusion section (Lines: 281, 396; marked with yellow).

20.Comments: Study limitations should also include: small sample size, change in primary and secondary outcomes.

Author response: We do think this is good suggestion, which will make our paper more rigorous. We have added small sample size, change in primary and secondary outcomes in the limitations (marked up yellow, Lines: 387-389)

Minor comments:

1.Comments: Abstract, line 3 and 4. In order to improve clarity, it should read: “We assessed whether opioid-free anesthesia would provide effective analgesia-antinociception monitored by analgesia index in video-assisted thoracoscopic surgery.”

Author response: We do think this is good suggestion, which will make our paper more clarity. We have modified it.

2.Comments: Abstract, line 11. It should read: “Secondary outcomes were depth of sedation monitoring by wavelet index and blood glucose concentration.”

Author response: We do think this is good suggestion, we have corrected it.

3.Comments: What does “recovery time” and “extubation time” mean? Please improve clarity.

Author response: We do think this is good question. We have explained the recovery time and extubation time in Table 1 in detail.

4.Comments: Results. Maybe the smoker status should be reported.

Author response: We do think this is good suggestion. Unfortunately, we did not record whether the patient had a history of smoking when collecting the data. When the 5-year follow-up of the patient’s survival, we will include whether the patient has a history of smoking. We appreciate your suggestion.

We should like to express our appreciation to you for suggesting how improve our paper.

List of Responses

Reviewer #6:

##### Dear Reviewer: Thank you for your letter and for the comments concerning our manuscript entitled “Lower sedation index and raised blood glucose with opioid-free anesthesia compared to opioid anesthesia for lung cancer patients undergoing video-assisted thoracoscopic surgery: a prospective, randomized, controlled study” (PONE-D-20-27554). Those comments are all valuable and very helpful for revising and improving our paper, as well as the important guiding significance to our research. We have studied the comments carefully and have made corrections which we hope meet with approval. Revised portion were marked in yellow in the paper. The main corrections in the paper and the responses to the reviewer’s comments are as flowing:

**Responses to the reviewer’s comments:**

1. Comments：In the abstract, and throughout, refrain from using p>0.05 and <0.05 or 0.01. Use precise values to 1 or 2 significant figures.

Author response: We do think this is a good suggestion and have changed to a more precise *p*-value (yellow-marked; Lines: 15, 17, 20, 21).

2. Comments：Abstract: extra space in were.

Author response: We are deeply sorry for our incorrect writing and have made correction.

3. Comments：Line 82: “patients were included after the investigators had explained the deviations from the protocol” surely there were no intended deviations from our original protocol? What is meant by this - the study protocol should have been adhered to?

Author response: We do think this is a good question. To avoid the influence of midazolam on cognitive function of the elderly after surgery, we did not use midazolam before general anesthesia induction. Before the patient signs the informed consent form, inform the patient of this change, and sign the informed consent form after the patient agrees. Sorry for the misspelling and we have made correction “pregnant”.

4. Comments：Line 94: was the study design one of non inferiority? If so, what was the non inferiority margin? It looks like the sample size calculation was actually to detect a difference in groups of 6 units of PtI, but I don’t think that was the intention?

Author response: We do think these are good questions. The statistical consultant determined the non-inferiority margin of this study based on the mean and standard deviation of PTI in the pre-experimental, and the intraoperative range of PTI，so the non-inferiority margin for this study was determined to be 6.

5. Comments：Line 164: dose rather than does

Author response: We are sorry for our incorrect writing and have made correction “dose”.

6. Comments：Line 169: began rather than begun.

Author response: We are very sorry for our incorrect writing and have made correction “began.”

7. Comments：Stats methods: was consideration given to using a mixed model?

Author response: We do think this is a good question, we did not consider applying a mixed effects model. The statistical methods used in this study are based on relevant literature and consulting statistics professors.

1. P. Ziemann-Gimmel, A. A. Goldfarb, J. Koppman and R. T. Marema. Opioid-free total intravenous anaesthesia reduces postoperative nausea and vomiting in bariatric surgery beyond triple prophylaxis. British Journal of Anaesthesia, 2014;112: 906–11
2. Uddalak Chattopadhyay, Suchismita Mallik, Sarmila Ghosh, Susmita Bhattacharya, Subrata Bisai, Hirak Biswas. Comparison between propofol and dexmedetomidine on depth of anesthesia: A prospective randomized trial. Journal of Anaesthesiology Clinical Pharmacology; 2014; 30:550-554
3. Kaushic A. THEERTH , Kamath SRIGANESH, K. Madhusudan REDDY, Dhritiman CHAKRABARTI, Ganne S. UMAMAHESWARARAO. Analgesia Nociception Index-guided intraoperative fentanyl consumption and postoperative analgesia in patients receiving scalp block versus incision-site infiltration for craniotomy. Minerva Anestesiologica 2018; 84:1361-1368

8. Comments：What was the adjustment considered for so many tests done? For multiple testing does an adjustment need to be made?

Author response: We do think these are good questions. The PTI, WLI, MAP and HR measurement time points in this study are related to the surgical procedure, and the blood gas collection time point is related to the operation time. Intraoperative adjustments are all adjusted by the anesthesiologist's judgment.

9. Comments：Line 188: use “.” Before Clinical.
Author response: Thank you for pointing these out, we have corrected it.

10. Comments：Table 1: wedge resection/lobectomy 7/40 is only 47 for OFA. What about the other 2? And 2 people missing for sites of VATS?

Author response: Thank you for pointing these out, it is ours mistake. In the OFA group, one patient underwent left lower lobe resection and upper lobe wedge resection, and one patient underwent right upper lobe resection and lower lobe wedge resection, so they are not counted. We have made revisions and re-statistical analysis (see Tab 2, marked-yellow).

11. Comments： Line 202 talks about repeated measures analysis. This wasn’t mentioned in the stats method section?

Author response: We do think these are good questions. We have marked in yellow in the method section “Within and between-group comparisons of either PTI, WLI, MAP and HR at six time points and PaO2, lactic acid levels and blood glucose concentrations at four time points were performed using repeated measurement analysis of variance.”

##### Once again, Special thanks to you for your good comments and suggestions.
