# Supplementary material for: Opioid-free anesthesia compared to opioid anesthesia for lung cancer patients undergoing video-assisted thoracoscopic surgery: A randomized controlled study
Source: PLoS One. 2021 Sep 23;16(9):e0257279. doi: 10.1371/journal.pone.0257279 (PMC8460000; doi:10.1371/journal.pone.0257279)
Supplement: Attachment — Submitted filename: Rebuttal Letter-3.docx [file pone.0257279.s007.docx]

Dear Dr Alessandro Putzu:

On behalf of my co-authors, we thank you very much for giving us an opportunity to revise our manuscript, we appreciate editor and reviewers very much for their positive and constructive.

Comments and suggestions on our manuscript entitled ‘Lower sedation index and raised blood glucose with opioid-free anesthesia compared to opioid anesthesia for lung cancer patients undergoing video-assisted thoracoscopic surgery: a prospective, randomized, controlled study’(PONE-D-20-27554) are all valuable and immensely helpful for revising and improving our paper, as well as the important guiding significance to our research. We have studied reviewer’s comments carefully and have made revision which marked in yellow in the paper. We have tried our best to revise our manuscript according to the comments.

We would like to express our great appreciation to you and reviewers for comments on our paper. We would be glad to respond to any further questions and comments that you may have. Looking forward to hearing from you.

Thank you and best wishes.

Yours sincerely,

Xuelian Zhao

4/5/2021

Here is our response to academic editors.

Major comments:

1. Comments. Methods, page 5, “Eligible patients were included after investigator had explained the deviations from our original protocol and obtained informed consent form signed by the patient.”. What does this sentence mean? Did you explain which kind of deviations to whom? Please improve clarity.
Author response: Thank you for point this out which will make paper clearer. We have rewritten the participant eligibility and consent of method, see Lines 86-89 with yellow labels.

2. Comments. Methods, page 6, “Before the patient signs the informed consent form, inform the patient of this change, and sign the informed consent form after the patient agrees.”. What does this sentence mean? What is the change you are talking about? Please improve clarity.
Author response: Thank you for point this out which will make paper clearer. We have rewritten the participant eligibility and consent of method, see Lines 86-89 with yellow labels.

3. Comments. Page 6, “Since midazolam may affect the cognitive function of the elderly after surgery, we changed the experimental plan that all patients did not use midazolam before the induction of anesthesia”. This statement is of limited significance and importance since you included patients 18-65 years old
Author response: Thank you for point this out. It has been reported that midazolam is related to postoperative delirium. Our clinical observations showed that even if it is not for elderly patients with delirium after thoracic surgery, the effect of midazolam cannot be ruled out. Therefore, midazolam is no longer used before anesthesia in patients undergoing thoracic surgery in our hospital. We have rewritten the participant eligibility and consent of method, see Lines 86-89 with yellow labels.

4. Comments. Page 6, “To facilitate the emergency treatment of patients during the operation, the anesthesiologists who did not participate in the assessment of the patients at any time were aware of the protocol”. This sentence is unclear and should be improved.
Author response: Thank you for pointing this out. We have rewritten the sentence, see Lines 114 with yellow labels.

5. Comments. Page 6, line 105-106. Details on number of enrolled patients should be reported only in the Results. Please remove this detail from the methods.
Author response: We do think these are excellent suggestions. We have deleted the details on number of enrolled patients (Lines: 109-110).

6. Comments. Table 1. What Namefen is? Was it used only in OA group? Why? Please improve clarity and insert a reference on this drug.
Author response: We do think this is a good question.

Nalmefene is a mu, kappa, alpha opioid receptor blocker, especially has a strong affinity for mu receptors. Nalmefene has a long action time, high bioavailability and few side effects. Clinically, nalmefene can inhibit or reverse the respiratory depression, sedation and hypotension effects of opioids (Chen Yingqi, Yue Yun, Qing Enming, et al. Nalmefene antagonizes the effect of opioid postoperative respiratory depression-a multicenter, randomized, double-blind, positive drug control study. Chinese Journal of Anesthesiology, 2011,31(3):307-309)

Jia reported that low-dose nalmefene prevents remifentanil-induced postoperative hyperalgesia in patients undergoing gynecological laparoscopic surgery under general anesthesia (Jia Zhen, Chen Yi, Zhang Linlin, et al. The effect of different low-dose nalmefene in preventing postoperative hyperalgesia induced by remifentanil. Chinese Journal of Anesthesiology, 2017, 37(10): 1159-1162.)

Yes, only patients in group OA received nalmefene to reverse the respiratory depression of opioids. We have insert reference on nalmefene in table 1.

7. Comments. Primary outcomes. The protocol reported different primary and secondary outcomes. Please report in methods why you changed the pre-defined outcomes. These are limitations to report in the discussion.
Author response: Thank you for pointing this out. We added these items in the method and the discussion (Lines:190-195, 398-401, yellow marked) depending on your suggestions.

8. Comments. Major protocol changes should be reported in the manuscript and in the full form in the supplementary material, with reasons (see CONSORT checklist 3b).
Author response: We do think this is good suggestion. Since midazolam midazolam is related to the occurrence of delirium in patients after surgery, the administration of midazolam before anesthesia was cancelled. The eligible patient was informed that they aren’t received midazolam and signed a written informed consent form after obtaining the consent of the participant (Lines: 86-89, marked with yellow). We have added those in the method.

9. Comments. Outcomes. Time points (T1-6) are unclear, since are similar for DPI and blood gas analyses outcomes. I suggest to use: PTI0 (=baseline), PTI1…PTI5.For blood gas analysis (BGA) I suggest: BGA0 (=baseline), BGA1…BGA4.
Author response: We do think this is good suggestion, which will make our paper clearer and easier to understand. We have made changes in relevant places in the manuscript marked with yellow.

10. Comments. You registered the protocol at the end of the study (Date of Last Refreshed on:2020/7/5 22:02:01; date of first version: 2020/7/1 0:47:36). This is a major limitation and must be reported in the Abstract, Methods and Discussion. In the discussion a paragraph should be dedicated to discuss the limitations on protocol registration and change in primary outcomes. The certainty of evidence should be downgraded due to this major limitations. Results should be discussed as exploratory. Conclusions should be tempered.
Author response: Thank you for pointing this out. Because the original protocol only said to detect arterial blood gas, we added the protocol on 2020/7/5, specifying that the blood gas detection items are PH, PaO_2_, lactic acid and blood glucose.

We only applied to modify the protocol once, that is, we submitted an application to modify the protocol at 0:7 on 1/7/2020, and the webmaster replied to agree to the modification at 0:47 on 1/7/2020. After seeing your question, I checked the record, and indeed there were two revision records on 1/7/2020 and 5/7/2020, which made us very confused. After we contacted the webmaster, he replied that it might be a mistake in the website record, because the contents of the two records are exactly the same and the reason maybe the website passed our revision on 5/7/2020.

From: "frog" <393642308@qq.com>;

Delivery time: July 1, 2020 (Wednesday) 0:07 AM

Recipient: "China Clinical Trial Registration Center" <1193142709@qq.com>;

Subject: Application for Modification of Trial Scheme

Dear experts from China Clinical Trial Center:

Hello, now we need your help. We have two clinical studies, the registration topics are

1. The clinical study of opioid-free anesthesia for thoracoscopic lung surgery (registration number ChiCTR1800019479)

2. Clinical study of opioid-free anesthesia for laparoscopic surgery (ChiCTR1900021223) During the pre-registration of these two clinical trials in November 2018 and February 2019, we did not specify in detail what kind of monitoring the blood samples collected.

According to the latest requirements, we need to clearly indicate that the blood samples should be monitored. Detection of blood sugar and lactic acid.

We have added change in primary and secondary outcomes in the limitations (marked up yellow, Lines: 398-403)

From: "China Clinical Trial Registration Center" <1193142709@qq.com>;

Delivery time: July 1, 2020 (Wednesday) 0:47 AM

Recipient: "frog"<393642308@qq.com>;

Subject: Reply: Application for Modification of Trial Scheme Hello. Both trials are open, please update as soon as possible and submit for review.

China Clinical Trial Registry

World Health Organization International Clinical Trial Platform Level 1 Registration Agency

11. Comments. Authors decided not to employ a ITT analysis. I suggest to insert ITT data on safety (mortality, postoperative complications). The lack of an ITT analysis is a major limitation that should be reported in the discussion.
Author response: We do think this is good suggestion. The ITT analysis included all randomized patients to fully observe the effect of the intervention. Unfortunately, we did not follow up the excluded patients. We have added the limitation of lack of the ITT analysis in the discussion (Lines: 401-403, marked with yellow).

12. Comments. Statistical analysis. What was the adjustment considered for so many tests done? For multiple testing does an adjustment need to be made?
Author response: We do think this is good question. In our study, we have just compared the PTI value at different time points (PTI1-PTI5) to the PTI basic value at PTI0 with ANOVA, and the PTI values in OFA group to those in OA group value at the same time point with *t*-test, we did not make adjustment. As multiple testing, we only focus on the difference between the two groups, so we did not perform adjustment such as Bonferroni correction or FDR correction.

13. Comments. Results and CONSORT flow diagram. You said that 110 patients met inclusion criteria, but you assessed for eligibility much more than 110 patients. How many patients met exclusion criteria? This data should be reported (Not meeting inclusion criteria n=X, Declined to participate n=X, Other reasons n=X ).
Author response: We do think this is good suggestion. From November 2018 to March 2019, we included 222 patients who underwent thoracoscopic lobectomy. Among them, 35 were due to age > 65 years, 10 were due to BMI > 30%, and 5 had history of alcohol, 62 people had cardiopulmonary insufficiency, and 10 people refused to participate in the experiment. Eventually 100 participants entered the experiment. We have modified the relevant content, see Fig 1 and Lines 216.

14. Comments. What is exhaust time? Please improve clarity.
Author response: We do think this is good question. In the ward, the patient was asked the time from returning to the ward to the first fart. If the duration is less than 24h, it is recorded 1 day; 24h-48h is 2 days, 48h-72h is 3 days, and so on. We have added an explanation of the exhaust time (Lines: 235-237).

15. Comments. The English must be improved. I suggest to ask for professional help.
Author response: We do think this is good suggestion. We have found a professional editing agency to modify the abstract, introduction and discussion parts of the article, and submit the receipt as an attachment. The methods and results of this article have been modified by high-level English users.

Minor comments:

1. Comments. Page 5, “The study was registered with ClinicalTrials.gov (ChiCTR 1800019479).”. It should read: “The study was registered on Chinese Clinical Trial Registry (ChiCTR 1800019479).”.
Author response: We do think this is good suggestion, which will make our paper more clarity. We have modified it.

2. Comments. Please expand TPVB the first time you used it.
Author response: We do think this is good suggestion, we have corrected it at Page 2 and Liens 9 .

3. Comments. Methods. The “Power analysis and sample size calculation” paragraph should be moved below, before statistical analysis paragraph.
Author response: We do think this is good suggestion, we have corrected it.

4. Comments. “dizzy” should probably reads “dizziness”
Author response: We do think this is good suggestion, we agree that the expression of dizziness is more professional, and we have corrected it (Table 2).

5. Comments. CONSORT diagram. You should report reason of discontinuation of intervention.
Author response: We do think this is good suggestion, we have corrected it.

We should like to express our appreciation to you for suggesting how improve our paper.

##### 6. Review Comments to the Author Dear Reviewer: Thank you for your letter and for the comments concerning our manuscript entitled “Lower sedation index and raised blood glucose with opioid-free anesthesia compared to opioid anesthesia for lung cancer patients undergoing video-assisted thoracoscopic surgery: a prospective, randomized, controlled study” (PONE-D-20-27554). Those comments are all valuable and very helpful for revising and improving our paper, as well as the important guiding significance to our research. We have studied the comments carefully and have made corrections which we hope meet with approval. Revised portion were marked in yellow in the paper. The main corrections in the paper and the responses to the reviewer’s comments are as flowing:

**Responses to the reviewer’s comments:**

Reviewer #6: Thanks for the revisions to the paper. Many improvements were made, but there are still some points to clarify.

1. Comments: Pg 6. Line 100 (track changed version) I feel the statistical sample size calculation text in the manuscript does not adequately match with the words in the response to reviewers.

Author response: Thank you for pointing this out. This description is indeed inappropriate, and we have made changes (Lines 201-202, marked with yellow).

2. Comments:There is now more of a mismatch between 88 pts and 100 pts. Please explain.
Author response: We do think this is a good question. With a level of significance 0.05, a power 0.9, true difference 6 and SD 10, each group should include 44 patients. Considering that the postoperative follow-up was in the ward before discharge, the dropout rate was determined to be 10%-20%. The statistical instructor of this experiment considered that a sample size of 100 is acceptable.

3. Comments:The 10 patients who refused to participate - did they do so before or after randomisation? If so, say so, and specify the usual time frame between registration/ agreeing to participate and randomisation. This process needs further clarity.

Author response: We do think this is a good question. From November 2018 to March 2019, we included 222 patients who underwent thoracoscopic lobectomy. Among them, 35 were due to age > 65 years, 10 were due to BMI > 30%, and 5 had history of alcohol, 62 people had cardiopulmonary insufficiency, and 10 people refused to participate in the experiment. Eventually 100 participants entered the experiment. We have modified the relevant content, see Fig 1 and Lines 216.

Therefore, 110 patients refer to the composite inclusion criteria, and there are no exclusion factors. Patients who refused to participate in the experiment were not included in the experiment. Eligible patients who agree to participate in the experiment will receive a sequentially numbered, opaque, sealed envelopes (Lines:111-112).

##### Once again, Special thanks to you for your good comments and suggestions.
