# Supplementary material for: Opioid-free anesthesia compared to opioid anesthesia for lung cancer patients undergoing video-assisted thoracoscopic surgery: A randomized controlled study
Source: PLoS One. 2021 Sep 23;16(9):e0257279. doi: 10.1371/journal.pone.0257279 (PMC8460000; doi:10.1371/journal.pone.0257279)
Supplement: Attachment — Submitted filename: Rebuttal Letter-4.docx [file pone.0257279.s008.docx]

Dear Dr Alessandro Putzu:

On behalf of my co-authors, we thank you very much for giving us an opportunity to revise our manuscript, we appreciate editor and reviewers very much for their precious comments and advice.

Comments and suggestions on our manuscript entitled ‘Lower sedation index and raised blood glucose with opioid-free anesthesia compared to opioid anesthesia for lung cancer patients undergoing video-assisted thoracoscopic surgery: a prospective, randomized, controlled study’(PONE-D-20-27554) are all valuable and immensely helpful for revising and improving our paper, as well as the important guiding significance to our research. We have studied comments carefully and have made correction which we hope meet with approval. Revised portions are marked in yellow in the paper.

We would like to express our great appreciation to you and reviewers for comments on our paper. We would be glad to respond to any further questions and comments that you may have. Looking forward to hearing from you.

Thank you and best wishes.

Yours sincerely,

Xuelian Zhao

9/6/2021

Here is our response to academic editors.

Major comments:

1. Comments. You should insert authors contributions (see PLOS One guidelines)
Author response: Thank you for your suggestion. As suggested by reviewer, we have inserted author contributions in title page and submitted online.

2. Comments. I suggest to use a structured abstract (not exceeding 300 words)
Author response: We are grateful for the suggestion. To be more clearly and in accordance with the comment, we have converted the abstract into a structured abstract, see abstract with yellow labels.

3. Comments. Abstract. You should cite the EEG monitoring system in the abstract.
Author response: We agree with your comment. We have added the EEG monitoring system in the abstract, see Lines 14-15 with yellow labels.

4. Comments. Abstract. The TPVB abbreviation should be removed from the abstract.
Author response: Thank you for pointing this out. We have removed the TPVB abbreviation from the abstract, see Lines 11 with yellow labels.

5. Comments. Abstract, line 20-24. It should read: “This study showed that our OFA regimen achieved equally effective intraoperative pain threshold index compared to OA in video-assisted thoracoscopic surgery. Depth of sedation was significantly lower and blood glucose levels were higher with OFA.”
Author response: We do think these are excellent suggestions which makes the manuscript more rigorous and concise (Lines: 27-31, marked with yellow).

6. Comments. Introduction, line 39. It should read: “…respiratory depression [3], postoperative nausea and vomiting (PONV) [4] as well as postoperative delirium…”
Author response: Thank you for pointing this out. We have added ‘postoperative’ in lines 47 and marked with yellow.

7. Comments. Methods, trial design, line 85-88. This paragraph should be removed: “Because midazolam is associated with postoperative delirium, midazolam is no longer used before anesthesia in patients undergoing thoracic surgery in our hospital. The eligible patient was informed that they are not received midazolam and signed a written informed consent form after obtaining the consent of the participant.”
Author response: We deeply appreciate the reviewer’s suggestion.  We have deleted the paragraph (Lines:95-98, yellow marked), and added the sentence “The eligible patient signed a written informed consent form after obtaining the consent of the participant.” (Lines:106-107, yellow marked).

8. Comments.  Methods, allocation and blinding, line 97. You should not report results in the methods. It should read: “Patients were randomized in two groups (group OA and group OFA) using computer-generated random numbers (Excel® version 16)”
Author response: We do think this is good suggestion. We have corrected it, see line 110.

9. Comments. Methods, Anesthetic management protocol, page 8, line 140-141. It should read: “Sevoflurane was adjusted to maintain anesthesia. The use of midazolam was avoided. The patients were maintained on mechanical ventilation to keep the ….”
Author response: We do think this is excellent suggestion. We have made changes in relevant places in the manuscript marked with yellow, line 156-157.

10. Comments. Methods, outcomes, page 10, line 183. Exhaust time should be defined

Author response: Thank you for pointing this out. We have added the definition of exhaust time (Lines: 191, marked in yellow).

11. Comments. Methods, outcomes, page 10, line 185-190. The English of the paragraph should be improved.
Author response: We apologize for the language problems in the manuscript. Language presentation was improved with assistance from a native English speaker with appropriate research background (Lines: 193-203, marked with yellow).

12. Comments. Results, page 14, line 283-284. This sentence should be improved “No patient died, was transferred to ICU and respiratory dysfunction, and no patient was intubated again after the tracheal intubation was removed”. If judged correct, it should read: “No patient was transferred to the ICU, had respiratory insufficiency, need reintubation, or died during the follow-up”.
Author response: Our deepest gratitude goes to you for your careful work and thoughtful suggestions that have helped improve this paper substantially (Lines: 303-307, marked with yellow).

13. Comments. Discussion, first paragraph, line 290-295. It should read: “This prospective randomized study showed that our OFA regimen could provide equally adequate intraoperative analgesia-nociception balance in non-obese, 18-65 years old, ASA I-II patients compared to opioid based anesthesia guided by PTI monitoring in patients undergoing VATS. However, in group OFA, the intraoperative depth of sedation was deeper, and the blood glucose levels were higher, than those levels in patients with OA.”
Author response: Thank you for your precious comments and advice. The comment is all valuable and extremely helpful for revising and improving our paper. We have revised the manuscript accordingly, see line 313-318and yellow-marked.

14. Comments. Discussion, line 312-313. Ij judged correct, it should read: “…sevoflurane and TPVB were used in our study to replace opioids[26].”
Author response: Thank you for pointing this out. We have re-written this sentence (Lines: 340, yellow-marked).

15. Comments. Discussion, page 16, line 332. It should read: “…postoperatively improved pain relief (especially pain on coughing) up to 48 h….”
Author response: Thank you for pointing this out. We have rewritten this sentence (Lines: 359-361, marked with yellow).

16. Comments. Discussion, page 17, line 358. It should read: “Our results suggested that, in patients under non-opioid anesthesia…”

Author response: We do think this is good suggestion, which will make our paper more clarity. We have modified it (Lines: 389, marked with yellow).

17. Comments. Discussion, page 19, limitations paragraph, line 391. It should read: “Fourth, our study was a single-institution study, small sample size, and some protocol amendments were performed. More in details, no data on PI were reported since PI can be only used in conscious patients and the serum potassium concentration was not recorded, because some patients were given potassium supplementation, which potentially results in a selection bias. Fifth, we cannot perform an intention-to-treat analysis including the 3 patients excluded after randomization since we did not follow up these patients. Further studies will be needed to assess the long-term benefits of the avoidance of opioid use in lung cancer patients, as well as to assess the benefits of increasing survival.”

Author response: Thank you very much for revising this part. You have given me a more rigorous understanding of English wording and sentence making. According to your comment, we have rewritten this part, see line 425-437 and marked with yellow.

18. Comments. Conclusions, line 402-405. It should read: “OFA with dexmedetomidine and sevoflurane plus TPVB appears to be feasible for the management of intraoperative nociception in ASA I-II, 18-65 years old, non-obese patients undergoing VATS for lung cancer. However, a deeper sedation level was produced in OFA than in OA for the achievement of an optimal balance of analgesia-nociception.”
Author response: Thank you for underlining this deficiency. This section was revised and modified according to the suggestion (Lines:440-442, marked with yellow).

19. Comments. Manuscript. The language quality must be improved. We advise that you seek assistance from a colleague or have a professional editing service.
Author response: Thank you for your careful review. We are deeply sorry for the inconvenience they caused in your reading. The manuscript has been thoroughly revised and rewritten by a native English speaker, so we hope it can meet the journal’s standard. The modified details have been marked in yellow.

20. Comments. Please review your reference list to ensure that it is complete and correct. If you have cited papers that have been retracted, please include the rationale for doing so in the manuscript text, or remove these references and replace them with relevant current references. Any changes to the reference list should be mentioned in the rebuttal letter that accompanies your revised manuscript. If you need to cite a retracted article, indicate the article’s retracted status in the References list and also include a citation and full reference for the retraction notice.

Author response: Thank you for your reminder, we have re-checked the references, and the changes are marked in yellow.

Note: The authors of this article, Nuoya Chen and Bingsha Zhao, have moved to Hengshui People’s Hospital and Tianjin Chest Hospital due to their job changes. We have revised their current addresses (see Title page),

Thank you for your careful review. We really appreciate your efforts in reviewing our manuscript. We wish good health to you, your family, and community. Your careful review has helped to make our study clearer and more comprehensive.
