# Supplementary material for: Opioid-free anesthesia compared to opioid anesthesia for lung cancer patients undergoing video-assisted thoracoscopic surgery: A randomized controlled study
Source: PLoS One. 2021 Sep 23;16(9):e0257279. doi: 10.1371/journal.pone.0257279 (PMC8460000; doi:10.1371/journal.pone.0257279)
Supplement: Attachment — Submitted filename: Rebuttal Letter-5.docx [file pone.0257279.s010.docx]

Dear Dr Alessandro Putzu:

On behalf of my co-authors, we thank you very much for giving us an opportunity to revise our manuscript, we appreciate editor and reviewers very much for their precious comments and advice.

Comments and suggestions on our manuscript entitled ‘Lower sedation index and raised blood glucose with opioid-free anesthesia compared to opioid anesthesia for lung cancer patients undergoing video-assisted thoracoscopic surgery: a prospective, randomized, controlled study’(PONE-D-20-27554) are all valuable and immensely helpful for revising and improving our paper, as well as the important guiding significance to our research. We have studied comments carefully and have made correction which we hope meet with approval. Revised portions are marked in yellow in the paper.

We would like to express our great appreciation to you and reviewers for comments on our paper. We would be glad to respond to any further questions and comments that you may have. Looking forward to hearing from you.

Thank you and best wishes.

Yours sincerely,

Xuelian Zhao

9/7/2021

Here is our response to academic editors.

Major comments:

1. Comments. Please add a reference. A good quality review on the topic (analgesia monitoring) would be of the great interest for the reader (Lines 69-71).
Author response: Thank you for your suggestion. As suggested by reviewer, we have inserted a review [17] in line 71 on analgesic index monitoring (Thomas Ledowski. Objective monitoring of nociception: a review of current commercial solutions. British Journal of Anaesthesia, 2019;123 (2): e312ee321).

2. Comments. This is unclear (Lines 177). Clarity should be improved. "First exhaust" should be replaced.
Author response: We are grateful for the suggestion. To be more clearly and in accordance with the comment, we have improved the exhaust time which is the time from returning to the ward to the patient complaining of the first breaking wind (Lines: 179-180).

3. Comments. The reason is different that reasin in Figure 1 caption. Please modify (Lines 211-212).
Author response: Sorry for our negligence. We have modified the reasons for exclusion from the analysis in Figure 1and marked with yellow.

4. Comments. It is unclear (Lines: 288-292). English and clarity should be improved.
Author response: Thank you for your careful review. The manuscript has been thoroughly revised and rewritten by Elsevier Language Editing, we hope it can meet the journal’s standard. The modified details have been marked in yellow (Lines:290-295).

5. Comments. This is a repetition of the first paragraph (Lines: 293-295)?
Author response: We do think these are excellent suggestions which makes the manuscript more rigorous and concise. We have improved this paragraph (Lines: 296-297, marked with yellow).

6. Comments. This long paragraph should be shortened. Clarity should be improved (Lines: 354-378).
Author response: We do think these are excellent suggestions which makes the manuscript more rigorous and concise. We have abbreviated this part in lines 360-386 and marked with yellow.

7. Comments. The strict inclusion criteria and study's limitations limit may limit the external validity of the study (Lines: 404).
Author response: We deeply appreciate the reviewer’s suggestion.  We have rewritten the sentence as We pay more attention to the changes in blood glucose levels of diabetic patients with OFA (Lines:413-414, yellow marked).

Thank you for your careful review. We really appreciate your efforts in reviewing our manuscript. We wish good health to you, your family, and community. Your careful review has helped to make our study clearer and more comprehensive.
