# Supplementary material for: Opioid-free anesthesia compared to opioid anesthesia for lung cancer patients undergoing video-assisted thoracoscopic surgery: A randomized controlled study
Source: PLoS One. 2021 Sep 23;16(9):e0257279. doi: 10.1371/journal.pone.0257279 (PMC8460000; doi:10.1371/journal.pone.0257279)
Supplement: Attachment — Submitted filename: Rebuttal Letter-6.docx [file pone.0257279.s011.docx]

Dear Dr Alessandro Putzu:

On behalf of my co-authors, we thank you very much for giving us an opportunity to revise our manuscript, we appreciate editor very much for their precious comments and advice.

Comments and suggestions on our manuscript entitled ‘Lower sedation index and raised blood glucose with opioid-free anesthesia compared to opioid anesthesia for lung cancer patients undergoing video-assisted thoracoscopic surgery: a prospective, randomized, controlled study’(PONE-D-20-27554) are all valuable and immensely helpful for revising and improving our paper, as well as the important guiding significance to our research. We have studied comments carefully and have made correction which we hope meet with approval. Revised portions are marked in yellow in the paper.

We would like to express our great appreciation to you for comments on our paper. We would be glad to respond to any further questions and comments that you may have. Looking forward to hearing from you.

Thank you and best wishes.

Yours sincerely,

Xuelian Zhao

22/7/2021

Here is our response to academic editors.

Major comments:
1. Comments. Abstract, page 2, line 18-19. It should read: “One hundred patients were randomized: 3 patients were excluded due to discontinued intervention and 97 included in the final analysis”.
Author response: Thank you for your suggestion. As suggested by reviewer, we have rewritten this sentence, line 17-19 and marked with yellow.

2. Comments. Abstract, page 3, line 23. It should read: “… the blood glucose levels in group OFA increased by 20% compared”
Author response: We are grateful for the suggestion. To be more clearly and in accordance with the comment, we have changed ascend to increased (Lines: 24, marked with yellow).

3. Comments. Abstract, conclusions. There is an error and information on limitations should be reported. It should read: “This study suggested that an OFA regimen achieved equally effective intraoperative pain threshold index compared to OA in video-assisted thoracoscopic surgery. Depth of sedation and blood glucose levels were higher with OFA. Study's limitations and strict inclusion criteria may limit the external validity of the study, suggesting the need of further randomized trials on the topic.”

Author response: We do think these are excellent suggestions which makes the manuscript more rigorous and concise. We have added “Study's limitations and strict inclusion criteria may limit the external validity of the study, suggesting the need of further randomized trials on the topic” (Lines 30-32 and marked with yellow).

4. Comments. Page 5, line 71. It should read: “A variety of analgesia monitors have been developed to determine the depth of pain during surgery [17].”

Author response: Thank you for your suggestion. The modified details have been marked in yellow (Lines:67).

5. Comments. Page 6, line 92-99. It should read: “We included patients with ASA physical status I or II, aged 18-65 years who were scheduled for elective thoracoscopic radical resection of lung cancer. Exclusion criteria were: pregnancy; breastfeeding; allergy to any experimental drug or its excipients; β-blockers therapy and HR <50 bpm; body mass index (BMI) more than 30 kg m−2; central nervous system diseases (such as epilepsy, cerebral infarction, or cerebral hemorrhage history); and history of chronic pain, alcohol, or drug abuse. The eligible patients signed a written informed consent form after obtaining the consent of the participant.”
Author response: We do think these are excellent suggestions which makes the manuscript more rigorous and concise. We have improved this paragraph (Lines: 89-97, marked with yellow).

6. Comments. Figure 1. The name of each group is missing in the figure. Please report OFA et OA labels.
Author response: We do think these are excellent suggestions. We have added label of OFA and label of OA (Lines 222-223 and marked with yellow).

7. Comments. Figure 1. Reasons for exclusions are reported twice (in the figure and in the caption). Please remove reasons for exclusion from figure caption. It should read: “Fig. 1 – Study flow diagram. OFA, opioid-free anesthesia; OA, opioid anesthesia”
Author response: We deeply appreciate the reviewer’s suggestion.  We have rewritten the sentence (Lines:219-223, yellow marked).

8. Comments: Page 11, line 213-216. It should read: “Three patients were excluded due to discontinued intervention: 1 case turned to open surgery, 1 peripheral venous catheter slid out during surgery, 1 patient needed re-exploration for postoperative intra-thoracic bleeding on postoperative day 1. Outcomes of these patients were not recorded in the postoperative period. The remaining 97 patients were included in the final analysis (Fig 1).”

Author response: We deeply appreciate the reviewer’s suggestion.  We have rewritten the sentence (Lines:212-216, yellow marked).

9. Comments: Exhaust time definition is still unclear. “The time from returning to the ward to the patient complaining of the first breaking wind” is totally unclear. Clarity should be improved.

Author response: We are grateful for the suggestion. Since the patients in this study were sent back to the ward about 10min-20min after the tracheal tube was removed, the exhaust time was recorded from returning to the ward to the first breaking wind.

To be more clearly and in accordance with the comment, we have changed the exhaust time from the end of the operation to the first breaking wind (Lines: 179, 232 and marked with yellow).

10. Comments: Page 15, line 303-304. It should read: “…of OFA for patients under general anesthesia.”

Author response: We deeply appreciate the reviewer’s suggestion.  We have corrected the sentence (Lines:299, yellow marked).

11. Comments: Page 20, line 396-397. It should read: “Fourth, our research is a study a single-institution study, with a small sample size”.

Author response: We deeply appreciate the reviewer’s suggestion.  We have corrected the sentence (Lines:383, yellow marked).

12. Comments: 11- Page 20, conclusions. It should read: “OFA with dexmedetomidine and sevoflurane plus TPVB seems to be feasible for the management of intraoperative nociception in ASA I-II, 18-65-years old, non-obese patients undergoing VATS for lung cancer. However, a deeper sedation level was produced in OFA than in OA for the achievement of an optimal balance of analgesia-nociception. A significant elevation in blood glucose levels in OFA group was also found. Study's limitations and strict inclusion criteria may limit the external validity of the study, suggesting the need of further randomized trials on the topic.”

Author response: We do think these are excellent suggestions which makes the manuscript more rigorous and concise. We have improved this paragraph (Lines: 396-404, marked with yellow).

Thank you for your careful review. We really appreciate your efforts in reviewing our manuscript. We wish good health to you, your family, and community. Your careful review has helped to make our study clearer and more comprehensive.
