# Supplementary material for: Opioid-free anesthesia compared to opioid anesthesia for lung cancer patients undergoing video-assisted thoracoscopic surgery: A randomized controlled study
Source: PLoS One. 2021 Sep 23;16(9):e0257279. doi: 10.1371/journal.pone.0257279 (PMC8460000; doi:10.1371/journal.pone.0257279)
Supplement: Attachment — Submitted filename: Rebuttal Letter-7.docx [file pone.0257279.s013.docx]

Dear Dr Alessandro Putzu:

On behalf of my co-authors, we thank you very much for giving us an opportunity to revise our manuscript, we appreciate editor very much for their precious comments and advice.

Comments and suggestions on our manuscript entitled ‘Lower sedation index and raised blood glucose with opioid-free anesthesia compared to opioid anesthesia for lung cancer patients undergoing video-assisted thoracoscopic surgery: a prospective, randomized, controlled study’(PONE-D-20-27554) are all valuable and immensely helpful for revising and improving our paper, as well as the important guiding significance to our research. We have studied comments carefully and have made correction which we hope meet with approval. Revised portions are marked in yellow in the paper.

We would like to express our great appreciation to you for comments on our paper. We would be glad to respond to any further questions and comments that you may have. Looking forward to hearing from you.

Thank you and best wishes.

Yours sincerely,

Xuelian Zhao

1/8/2021

Here is our response to academic editors.

Major comments:
1. Comments. Exhaust time is not an appropriate english medical term; you should change it. What does it mean? Do you mean " Time to passage of flatus and stool"? "Time to passage of flatus"? " Time to passage of stool"? Please modify.

Author response: We do think these are excellent suggestions which makes the manuscript more rigorous and concise. Anal exhaust is one of the indications of postoperative intestinal function recovery. The anal exhaust time was defined from the end of operation to the first spontaneous breaking wind event (Lines: 176-177, 228-229, Table 2, marked with yellow).

2. Comments. The details in Figure 1 are still missing (see PDF). You should include the denomination of each group.

Author response: Sorry for the omission in Figure 1, we have added denomination of each group (Figure 1).

3. Comments. Some other modifications are reported in the PDF attached.

Author response: We deeply appreciate the reviewer’s suggestion. We have made modifications according to the annotations in the PDF and marked with yellow.

Thank you for your considerate review. We really appreciate your efforts in reviewing our manuscript. We wish good health to you, your family, and community. Your attentive review has helped to make our study clearer and more comprehensive.
