# Supplementary material for: Opioid-free anesthesia compared to opioid anesthesia for lung cancer patients undergoing video-assisted thoracoscopic surgery: A randomized controlled study
Source: PLoS One. 2021 Sep 23;16(9):e0257279. doi: 10.1371/journal.pone.0257279 (PMC8460000; doi:10.1371/journal.pone.0257279)
Supplement: Attachment — Submitted filename: Rebuttal Letter-8.docx [file pone.0257279.s014.docx]

Dear Dr Alessandro Putzu:

On behalf of my co-authors, we thank you very much for giving us an opportunity to revise our manuscript, we appreciate editor very much for their precious comments and advice.

Comments and suggestions on our manuscript entitled ‘Lower sedation index and raised blood glucose with opioid-free anesthesia compared to opioid anesthesia for lung cancer patients undergoing video-assisted thoracoscopic surgery: a prospective, randomized, controlled study’(PONE-D-20-27554) are all valuable and immensely helpful for revising and improving our paper, as well as the important guiding significance to our research. We have studied comments carefully and have made correction which we hope meet with approval. Revised portions are marked in yellow in the paper.

We would like to express our great appreciation to you for comments on our paper. We would be glad to respond to any further questions and comments that you may have. Looking forward to hearing from you.

Thank you and best wishes.

Yours sincerely,

Xuelian Zhao

21/8/2021

Here is our response to academic editors.

Major comments:
1. Comments. “Anal exhaust time” is not a current appropriate English medical term. Please use "Time to passage of flatus".
Author response: We do think this is excellent suggestion which makes the manuscript more rigorous and concise. Thank you very much for pointing out our unprofessional use of words. The time to passage of flatus you provided is a standard expression. (Lines: 176-177, 229-230, Table 2, marked with yellow).

2. Comments. I received a comment from Staff Editor. There are some deviations between the protocol, registry entry, and the manuscript. This it should be further explained by the authors. You can add the detailed list of protocol changes in the supplementary material. Most important changes should be reported in the methods. If you have any queries or need any help, please contact plosone@plos.org.
Author response: Sorry for the inconvenience caused by the protocol changes. We summarize the changes in this research protocol in a table and submit it as a supplement (S File 9). The intraoperative PI and serum potassium are important changes that are not recorded. We explain the reasons and write them in the method and discussion section of the manuscript (Lines: 180-186, 385-387 and marked with yellow).

3. Comments. You should review your reference list to ensure that it is complete and correct, as reported above. No retracted manuscript should be cited.

Author response: We deeply appreciate the reviewer’s suggestion. We have made modifications according to Plos One's reference requirements and marked with yellow.

Thank you for your considerate review. We really appreciate your efforts in reviewing our manuscript. We wish good health to you, your family, and community. Your attentive review has helped to make our study clearer and more comprehensive.
